# Criteria to evaluate unmet health-related needs of persons living with rare diseases and their caregivers: rapid literature review and stakeholder consultations

**DOI:** 10.1186/s13023-025-03838-6

**Published:** 2025-07-01

**Authors:** Zilke Claessens, Alice Vanneste, Charlotte Van Isterdael, Charlotte Verbeke, Io Wens, Isabelle Huys

**Affiliations:** Department of Pharmaceutical and Pharmacological Sciences, KU Leuven, Leuven, Belgium

**Keywords:** Rare diseases, Unmet health-related needs, Patient-centred healthcare framework

## Abstract

**Background:**

Rare diseases affect small populations but present unique challenges in access to healthcare and social support. The needs of patients and their caregivers extend beyond medical treatments, impacting various aspects of their lives. This study provides a narrative overview of these diverse needs experienced by patients and caregivers.

**Methods:**

A rapid literature review was conducted in PubMed and Embase, including studies assessing needs in rare diseases. Following Cochrane guidelines, two researchers screened 1.419 articles (74%) double-blinded, followed by a single researcher screening the remaining 509 articles (26%). Two researchers collaboratively extracted data into an extraction table. To validate and complement these findings, two stakeholder consultations were held with representatives from patient organisations, healthcare providers, the pharmaceutical industry, and policymakers.

**Results:**

A total of 272 articles were included in the review, and respectively 25 and 33 participants participated in the consultations. The identified needs were categorized into two levels: (i) patient needs, and (ii) caregiver needs, along with one overarching transversal need: (iii) information needs. Patient needs spanned health, healthcare, and social dimensions. Psychological, mental, and emotional health were frequently highlighted, but also autonomy emerged as a significant need. Healthcare needs included gaps in timely and accurate diagnoses, underscoring the need for more awareness among healthcare providers and appropriate diagnostic tools. Coordinated multidisciplinary care and accessibility to care and treatments were also identified as essential, yet lacking. Socially, patients experienced unmet needs in support networks, workplace inclusion, education, and financial stability. Caregivers’ needs were related to physical and mental health, social connection, and financial support. Information needs, affecting both levels and even extending to healthcare providers, underscored the demand for more comprehensive, accessible information on rare diseases, treatment options, healthcare services, and available social support.

**Conclusion:**

This study underscores the complex needs of persons living with rare diseases and their caregivers, advocating for a holistic approach in healthcare policy. Beyond medical interventions, addressing timely diagnosis, coordinated care, and psychological support are essential. Policymakers must consider these multifaceted needs to enhance patient outcomes and foster an inclusive, patient-centred healthcare framework.

**Supplementary Information:**

The online version contains supplementary material available at 10.1186/s13023-025-03838-6.

## Introduction

Good health is essential, not only for individual well-being but also for the overall functioning of society [1]. While modern healthcare systems aim to provide access to high-quality care, they often struggle to address the full spectrum of patients' needs [1]. A key challenge lies in the strong product-driven focus of healthcare decision-making, where healthcare systems evaluate and adopt products that enter the market, prioritizing treatments based on their perceived value for specific disease conditions. However, this supply-driven approach may overlook broader health-related needs that receive less attention from pharmaceutical developers, leaving many patients with unmet health-related needs.

The current proposal for the reform of EU pharmaceutical legislation aims to establish a more patient-centred regulatory framework by introducing new incentives that reward research and development (R&D) focused on targeting unmet medical needs, aiming to shift towards a more needs-driven healthcare system [2, 3]. This legislative package includes updated definitions of unmet medical needs, along incentives for developers addressing these needs [3]. However, accurately defining and prioritizing unmet medical needs remains a challenge, as highlighted in recent debates around the reform [3]. Furthermore, it remains primarily focused on pharmaceutical interventions, overlooking other critical aspects, such as access to healthcare services, social support, and financial assistance for patients and their caregivers.

To complement these legislative changes, a broader, more holistic framework that captures the wider spectrum of patients’ needs could enhance needs-driven decision-making at both European and national levels. An example of such an approach is the Need Examination, Evaluation, and Dissemination (NEED) assessment framework, developed by the Belgian Health Care Knowledge Centre (KCE) [4]. The NEED framework aims to identify unmet health-related needs across various conditions. Health-related needs are defined for the purpose of this study as “*the specific needs related to a particular health condition, which encompasses the needs of individuals affected by the condition, as well as broader societal needs that are the consequences of the condition on segments of society not directly afflicted by the condition (i.e. externalities). The health-related needs cover the entire health condition trajectory, from prevention to diagnosis, treatment, and rehabilitation or palliative care*” [4].

The KCE NEED framework consists of three domains (i.e. health, healthcare, social) and four dimensions (i.e. patients, society, future, and equity). Every dimension consists of criteria (e.g. impact of physical health, burden of treatment, impact on social life) described by indicators (e.g. pain/discomfort before vs today, experienced burden of treatment, social support needs) to be measured by one or a combination of the included data collection methods (i.e. literature reviews, qualitative research, expert consultations, patient surveys, and database analyses). Once the data is gathered, it is provided on the publicly available NEED database, where quantified data per condition can be found, intended to inform healthcare decision-making [4].

In the context of rare diseases, addressing unmet health-related needs is especially challenging due to small patient populations, complex, heterogeneous pathologies, and limited experience in managing these conditions [5]. Critical gaps such as accurate diagnosis, access to reliable information, and appropriate care are often unaddressed, while R&D is hindered by high costs and lower returns on investment [6]. Consequently, patients frequently face limited treatment options, with available therapies being costly and accompanied by uncertainties regarding their effectiveness. Given these challenges, addressing the high variability in unmet health-related needs of persons living with rare diseases remains critical [7].

This explorative study aims to identify and map criteria for unmet health-related needs that are specific to rare diseases. It focuses on holistic needs that extend beyond medical treatments, encompassing health, healthcare, and social dimensions. By gaining a deeper understanding of these multifaceted unmet health-related needs, the research seeks to offer a foundation for developing frameworks to identify needs, such as the KCE NEED framework, and inform decision-making processes that prioritize resources toward areas that effectively address patient and caregiver needs.

## Methods

### Rapid literature review

A rapid literature review was conducted in accordance with the Cochrane guidelines [8]. The protocol of this study has been published before data extraction in the Open Science Framework [9].

#### Search strategy design

A comprehensive search was conducted in PubMed and Embase, supplemented with backward citation screening by reviewing the bibliographies of included publications. The search string (supplementary material 1), developed with the research team under guidance of a biomedical information specialist, consisted of concepts related to: (i) rare diseases, (ii) research methods, and (iii) health-related needs. This string incorporated relevant synonyms and MeSH or Emtree terms for PubMed or Embase, respectively. Inclusion and exclusion criteria are provided in Table 1, and publications have to meet all inclusion criteria.

**Table 1:** Inclusion and exclusion criteria applied in the rapid literature review

Criteria	Inclusion	Exclusion
Publication type	All publication types except conference abstracts, preprints, clinical trials, editorials	Conference abstracts, preprints, clinical trials, editorials
Study design	Focus on • studies that conduct a primary needs assessment • studies that perform a secondary analysis, review or opinion of needs assessment data or literature, which could be in the form of a literature review, meta-analysis, database analysis, and opinion paper	Research that does not include the primary or secondary analysis of needs assessment data
Population	Focus of the publication is on rare diseases in general or on one specific rare disease	Focus of the publication is not on rare diseases or a specific rare disease
Outcome	The study conducts a needs assessment or discusses the conduct of needs assessment in the publication	No health/care/pharmaceutical needs assessment was conducted or discussed in the publication
Geographical region	High- and middle-income countries	Low-income countries

#### Screening and selection

Articles published before December 2023 were retrieved and manually deduplicated by one researcher (ZC) using Rayyan Software. Two researchers (ZC and AV) screened the selected articles (n = 1.419) based on the defined inclusion and exclusion criteria (Table 1). A pilot screening was performed to ensure consistency, where two researchers (ZC and AV) collaboratively screened 40 publications, followed by a double-blinded, parallel screening of 10% of the initially selected articles (n = 140), achieving a concordance rate of over 90%. After, all initially retrieved articles were screened double-blinded (ZC and AV) with disagreement resolved by consensus among the research team. After an update of the search string, the additionally retrieved 509 articles were screened by one researcher (ZC). The screening and selection process is reported according to the PRISMA guidelines [10].

#### Data extraction, analysis and synthesis

Data were extracted using a predefined Microsoft Excel extraction form (Supplementary material 2), which was updated throughout the extraction process to include new emerging topics not previously mentioned (deductive-inductive approach). The data extraction form contained descriptive parameters (authors, title, disease, year of publication), methodological details (primary or meta-research, methodological challenges), and criteria for identifying unmet health-related needs based on the pre-existing KCE NEED framework (e.g., autonomy, accessibility to care, impact on work) [4]. Diseases were classified using the WHO ICD-11 system [11]. A pilot extraction of 10 articles was conducted by two researchers (ZC and AV) and reviewed by the full team. Final extraction was performed by two researchers (ZC and AV). Data were analysed using a narrative analysis, with findings aggregated, grouped, and visualized.

### Stakeholder consultations and qualitative insights

In addition to the primary literature review, two multi-stakeholder consultations were organized to validate the literature findings. Additionally, one-on-one discussions (n = 3) were held for clarification when statements during consultations were found unclear or more in-depth insights were required according to the research team. The protocol for these stakeholder consultations was approved by the Ethics Committee Research UZ/KU Leuven in Belgium (S67909). Participants were invited using purposive and convenience sampling based on their expertise, experience, and availability [12]. The sampling strategy ensured a balanced representation of patient organisations, the pharmaceutical industry, regulatory authorities, policymakers, health technology assessment bodies, payers, and healthcare providers (HCPs), aimed at 20–30 participants per consultation.

Prior to the consultations, participants completed an introductory survey (Supplementary Material 3) related the consultations’ content to guide the discussion. Each consultation lasted two and a half hours and followed a predefined topic guide (Supplementary Material 4). The consultations covered the criteria to assess unmet health-related needs in rare diseases and related (methodological) challenges. Consultations were transcribed ad verbatim and thematically analysed using NVivo by two researchers (ZC and CVI). Insights were incorporated into the analysis for validation of the literature findings.

## Results

**Fig. 1:**
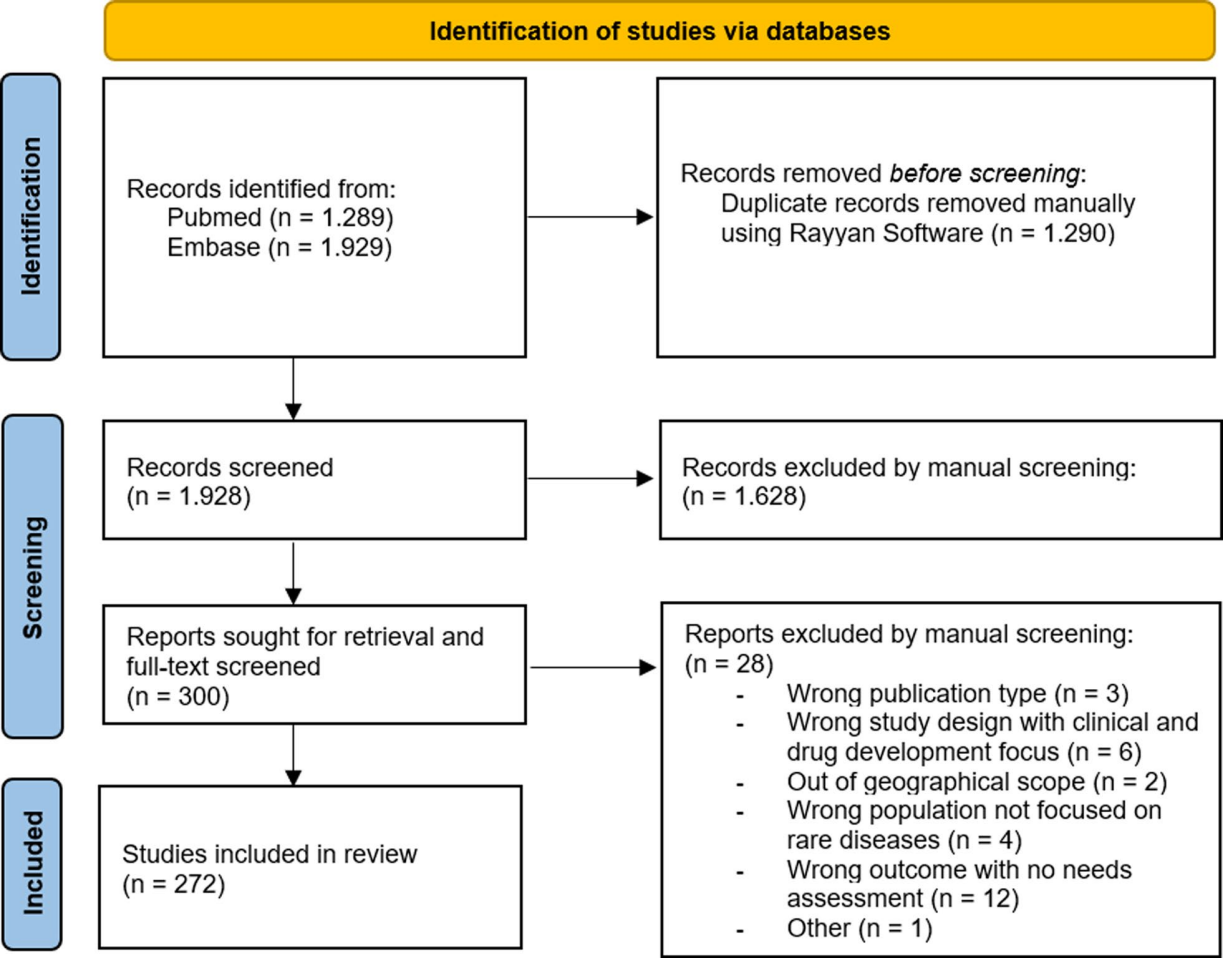
PRISMA flow diagram of the study screening and selection process for scientific literature included in the rapid review [10]

In total, 272 articles met the inclusion criteria and were included in the rapid review (Fig. 1).

**Fig. 2:**
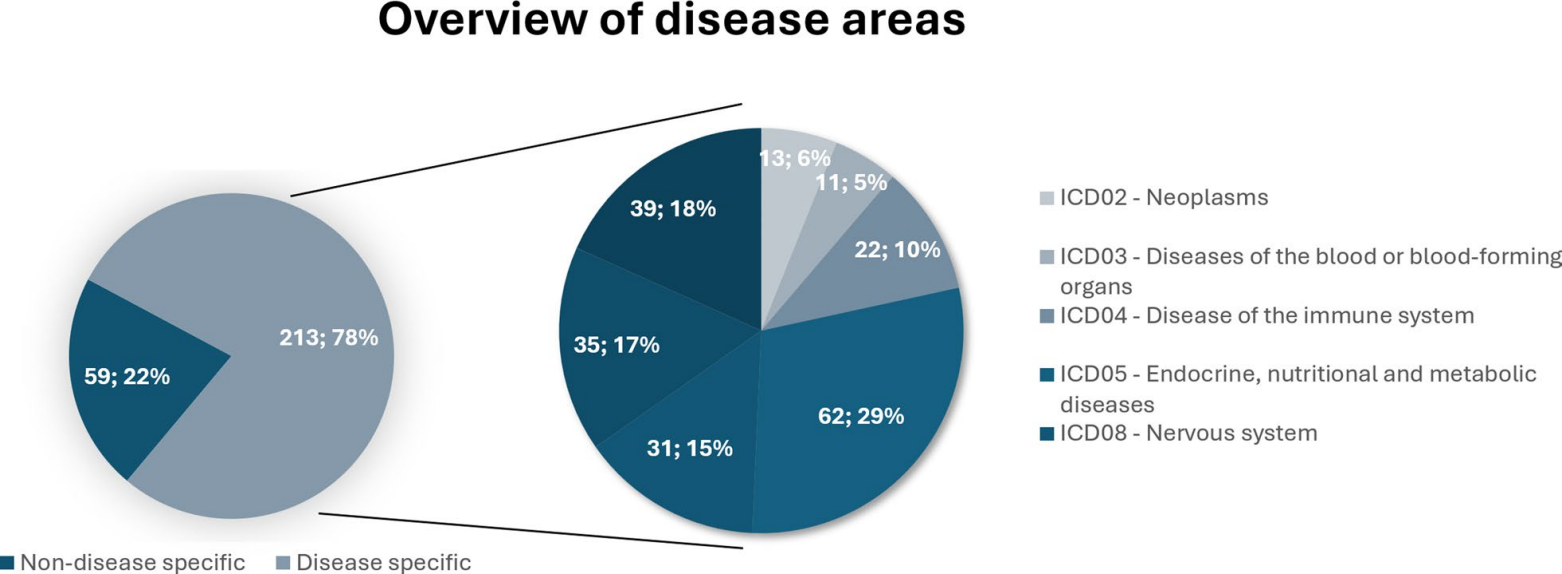
Rare disease area representation in the reviewed literature. Diseases were classified using the WHO ICD-11 system [11]

**Fig. 3:**
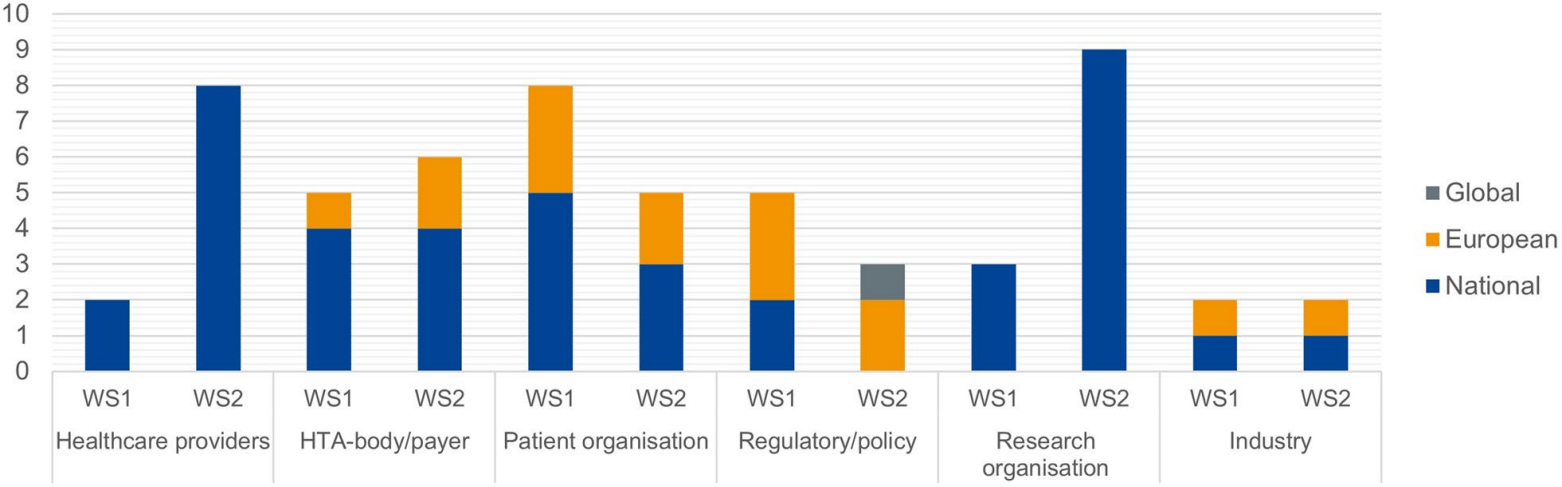
Characteristics of stakeholder representatives participating in the stakeholder workshop. *WS* Workshop, *HTA* Health technology assessment

Of the 272 included articles, most were published after 2014. Rare disease areas specified by the studies are presented in Fig. 2, with a summary of article characteristics detailed in Supplementary Material 5. Furthermore, 72 stakeholder representatives were invited to take part in the consultations, of which 25 participated in the first and 33 in the second consultation, as presented in Fig. 3.

**Fig. 4:**
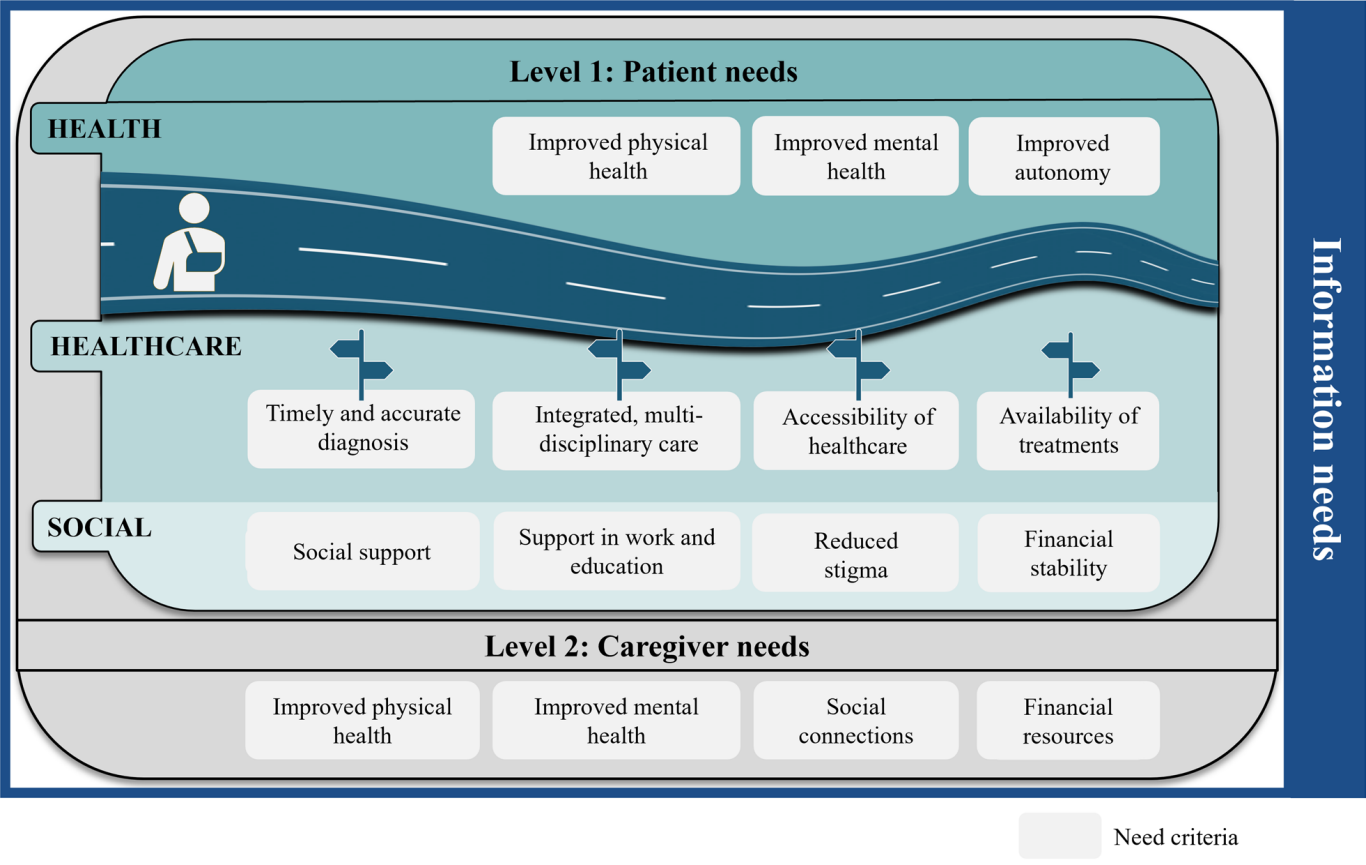
Patient journey with health, healthcare, and social needs, along with caregiver needs relevant in the context of rare diseases, as well as overarching information needs relevant across both levels

The literature review resulted in the identification of 15 need criteria relevant in the context of rare diseases, which could be divided into various levels, as shown in Fig. 4. The stakeholder consultations confirmed the relevance of all 15 criteria by emphasizing some criteria and provided more depth to their understanding, as explained below.

The first level addresses patient needs, as patients experience a range of unmet needs throughout their patient journey (Level 1: patient needs). These needs are further divided into three domains based on the type of need: (i) health, (ii) healthcare, and (iii) social. Within each domain, between three and four need criteria were identified. The second level focuses on caregiver needs and the significant challenges faced by informal caregivers, including family members or friends. These individuals play a crucial role in providing health and daily life support to patients, yet their needs often remain unmet (Level 2: caregiver needs). Within this level, four need criteria were identified. Lastly, an overarching transversal need was identified as relevant across both levels: information needs. Patients, caregivers, and HCPs frequently lack adequate information regarding rare diseases, treatment options, healthcare services, and available social support.

### Level 1: patient needs

At the patient level, the needs are categorized into three distinct domains, each addressing different aspects of the entire patient’s journey. These domains are: (i) health-related needs, relating to the direct impact of rare diseases on patients’ health status, (ii) healthcare-related needs, covering challenges patients encounter throughout their healthcare, and (iii) social needs, related to challenges on the social relationships of the patients (Fig. 4).

#### Health

Health-related needs in persons with rare diseases can be further categorized into three need criteria: (i) improved physical health, (ii) improved mental health, and (iii) improved autonomy. These aspects were frequently mentioned in literature and were reported as often being overlooked despite their significant impact on patients’ well-being.

(i) *Improved physical health* The literature highlights a wide range of physical health impacts experienced by persons living with rare diseases due to diverse symptoms like pain, fatigue, itching, and swelling. Fatigue, in particular, was common and debilitating across various disease areas as it often disrupted daily functioning [13–18].
(ii) *Improved mental health, including psychological aspects, cognitive functioning, and emotional well-being* Anxiety and depression were frequently reported psychological challenges, often driven by fears of disease progression, hopelessness about the future, and loss of control over their lives [13, 14, 16, 19–31]. This has also been emphasized during the consultations: “*Unpredictability of the way the disease will manifest throughout days, life, weeks is a very big burden and sort of a terrorist on your life that you cannot say when you will have good days or bad days because you cannot plan properly*”. Other emotional challenges and concerns included loneliness, cognitive impairments, aggression, irritability, and decreased sexual satisfaction [20, 22, 28, 31–33]. Furthermore, low self-esteem, particularly related to aesthetic concerns, was emphasized in both the literature and during the stakeholder consultations [34]. Mental health was often reported as a primary concern by patients. For example, in one study, 83% (n = 87) of respondents with Ehlers-Danlos syndrome identified mental health as an unmet need [28]. Another study of patients with Locked-in syndrome in Sweden showed a higher impact on quality-of-life scores in cognitive and mental domains than those in physical domains [35].
(iii) *Improved autonomy* The autonomy of persons living with rare diseases is often severely compromised due to the complex and persistent symptoms and the need for ongoing medical interventions. Frequent and lengthy medical appointments, combined with the limited availability of HCPs, force many patients to adapt their work and personal schedules which disrupts their sense of independence [36, 37]. Mobility impairments, including difficulties with walking, dressing, eating, and personal hygiene, further limit autonomy [30, 32, 38–42]. Many patients become reliant on others for supporting basic and professional daily activities, such as going to work or school, which can be particularly disempowering [43].

#### Healthcare

Regarding the healthcare of patients with rare diseases, many unmet needs have been identified in rare disease literature. The most recurring healthcare-related needs relate to four need criteria concerning: (i) timely and correct diagnosis, (ii) accessibility of healthcare, (iii) integrated, multi-disciplinary care, and (iv) availability of effective treatments.

(i) *Timely and correct diagnosis* Rare diseases are challenging to diagnose due to various factors such as non-specific symptoms, insidious onset, and multisystem involvement [44–46]. Some patients may never receive a definitive diagnosis, as highlighted in the stakeholder consultations. Diagnostic tests and screening methods are also often inadequate [47, 48]. For instance, a European survey with HCPs reported that 40% to 60% of respondents experienced unavailability of certain genetic diagnostic tests [47]. Another survey further emphasized the uneven availability of screening methods across European countries, further exacerbating delays and missed diagnoses [48].
(ii) *Accessibility of healthcare* The two frequently reported major barriers to healthcare accessibility for persons living with rare diseases include long waiting times for appointments and travel distances to specialized healthcare centers due to a scarcity of specialists and specialized centers in many regions [49–52]. A participant of the stakeholder consultations explained, “*For the access to care, it would be useful to question the accessibility to disease experts and the existence of expertise centers in Belgium or not. For some diseases, patients need to go to other European countries, which can represent a huge burden*”. Psychological care is particularly difficult to access due to a shortage of professionals and delays [53]. Geographic disparities further hinder timely access to specialized care, especially between urban and rural areas, where infrastructure and specialist availability vary widely [54]. One survey study found that 47% of persons living with rare diseases reported traveling over 100 km for care, illustrating the burden of accessing specialized services [54]. These challenges significantly hinder access to receiving necessary treatment and support [23, 55, 56].
(iii) *Integrated, multi-disciplinary care* Persons living with rare diseases often require integrated, multi-disciplinary care to address their complex needs. However, the literature indicates a gap in collaboration among specialists of different disciplines, resulting in fragmented care [21, 57–62]. In addition to medical treatment, these patients require holistic care that includes therapies such as physiotherapy, occupational therapy, speech therapy, and rehabilitation [37, 49]. Psychological and social support, such as emotional care for patients and families, are also often unmet [16, 24, 63–65]. The transition from pediatric to adult care is particularly challenging, disrupting continuity in care when adult services lack rare disease expertise [58, 66, 67]. The absence of well-coordinated clinics forces patients to visit multiple specialists, creating inefficiencies and reducing the quality of care and reducing the quality of care [34].
(iv) *Availability of effective treatments* The literature indicates that many rare diseases lack curative treatments, leaving patients reliant on symptomatic management [33, 40]. Even when treatments are available, high prices and inadequate reimbursement often hinder accessibility [28, 68–70]. For example, a focus group study on hyperemesis gravidarum in Ireland highlighted difficulties in obtaining prescriptions from local general practitioners and limited medication stocks in pharmacies [71]. Stakeholders in the consultations also noted the use of off-label medications, vitamins, and mineral supplementation, as well as patients sometimes seeking treatments in other EU countries when local options are insufficient.

#### Social

Persons living with rare diseases face a range of needs related to social aspects. Social related needs were extensively discussed during the consultations, where participants further explained: *“Also, these very rare diseases, that are very important are impatient with or try to have normal lives and to function in daily situation. So, we should not forget this and not limit or focus on the so-called wonder drugs and top doctors and whatever*”. Four need criteria were identified, including (i) social support, (ii) support in work and education, (iii) financial stability, and (iv) reducing stigma.

(i) *Social support* Persons living with rare diseases often experience isolation due to geographic dispersion and limited opportunities to connect with others in similar circumstances. Physical symptoms, such as visible signs of the disease or mobility limitations, can discourage social engagement, while feelings of stigma and emotional strain further complicate social interactions. Additionally, the disease’s impact on daily functioning often disrupts romantic relationships, friendships, and family dynamics, reducing their social networks [15, 42, 72–74]. Moreover, the need for guidance, on for intance mental healthcare, physical activity, and social care, is reported, leaving patients reliant on healthcare services without comprehensive resources for independent self-management [52, 75–79].
(ii) *Support in work and education* Rare diseases often affect children and young adults, disrupting educational progress. Cognitive, physical, and emotional challenges can impair concentration, while frequent hospital visits lead to school absenteeism and hinder academic continuity and social development, as seen in children with hemophilia and tuberous sclerosis [80–84]. Adults in the workplace frequently experience career disruptions, with many reporting reduced hours, altered responsibilities, or (in)voluntary job termination [82, 84–87]. For example, 25% of primary hyperoxaluria patients and 75% of Cushing’s syndrome patients report significant work impairments [61, 80]. Physical restrictions and the need for flexible or lower-stress roles limit career advancement, often leading to underemployment [64, 82, 88–91].
(iii) *Financial stability* Literature shows that persons living with rare diseases face substantial financial burdens that can significantly affect their quality of life. Studies found that 37% (n = 326) of patients with Cushing syndrome and 55% (n = 146) of adults with hemophilia reported a negative impact on their economic situation [61, 84]. While medication costs represent a large part of expenditures, non-medical therapies, diagnostic tests, and specific nutritional requirements are often not fully covered by insurance, adding to patients' financial strain [39, 49, 51, 59, 63, 92, 93]. For instance, a study in Spain indicated that over 22% (n = 163) of families caring for persons living with rare diseases could not afford psychological care [94]. Additional non-medical costs, including specialized clothing, mobility aids, home modifications, and long-distance travel for treatment, compound the financial burden [5, 19, 52, 83, 88, 95–97]. Informal and professional caregiving costs further exacerbate economic challenges [98–101].
(iv) *Reducing stigma* Stigma remains a significant burden for patients with rare diseases, as the literature and stakeholder consultations highlight its profound social impact [54, 79, 93, 102–107]. A lack of awareness, understanding, and familiarity with rare diseases lays at the basis of this stigmatisation, resulting from less attention for particular rare diseases and insufficient information due to limited research in this disease area [54, 87, 108].

### Level 2: caregiver needs

Informal caregivers play a pivotal role in providing essential support and care for patients with rare diseases, but this indispensable role often imposes a substantial, yet under recognized burden on caregivers’ well-being. Needs for caregivers can be further categorized into four need criteria: (i) improved physical health, (ii) improved mental health, (ii) social connections, and (iv) financial resources.

#### Improved physical health

Several studies demonstrated that caregiving for persons living with rare diseases can impose substantial physical demands, including lifting and transferring patients resulting in pain and fatigue [88, 109]. For example, 73.5% (n = 36) of caregivers for children with rare chronic kidney diseases reported changes in their physical health due to the caregiving duties [110]. Sleep deprivation and exhaustion, caused by limited rest and leisure opportunities, further strain caregivers [110, 111].

#### Improved mental health

Research indicates that the psychological burden on caregivers is equally, if not more, profound than the impact on their physical health [88, 112]. Caregivers experience anxiety from clinical uncertainties, fear about the future, self-blame, and guilt linked to the genetic transmission of the defective gene that causes the rare disease [20, 88, 112–114]. Depression is also common, with caregivers reporting moderate to severe symptoms [41, 91, 115, 116].

#### Social connections

The demanding nature of caregiving severely disrupts caregivers’ social lives, leaving limited time for personal activities or self-care and a perceived sense of isolation [117]. For example, 77% (n = 450) of caregivers for patients with Dravet syndrome reported having less than one hour daily for personal respite [118]. Caregiving strains also extend to personal relationships and may lead to separations or even marital breakdowns, particularly in cases involving untreatable diseases with no prognosis [81, 96, 112, 119]. Stakeholder consultations underscored the long-term social repercussions, which reverberate across generations.

#### Financial resources

Literature indicates a significant financial burden on caregivers and their families, including direct healthcare-related costs and indirect costs such as loss of income [120]. For instance, 65% of caregivers in one study reported taking time off from work for the healthcare needs of their children, while 56% of caregivers for patients with hereditary transthyretin amyloidosis reported changes in their employment status [41, 118]. Long-term caregiving can deplete family savings, as seen in caregivers of patients with Erdheim-Chester disease [121]. Participants in the consultations emphasised the need to consider this multifaceted financial impact on the broader family, extending beyond immediate costs [81]. Also during the consultation the financial impact of rare diseases was discussed: “*I just wanted to say that due to impact on work, there is a huge impact on parents, carers, informal carers. It also impacts the finances of the family. It impacts the whole family. It impacts the siblings. There are also import healthcare costs that are not covered. The financial consequences are not just non-healthcare costs, there are also healthcare costs that are not covered. That are quite substantial often, like for certain devices, for medical foods et cetera, that are not sufficiently covered*”.

### Transversal: information needs

Information needs represent a critical and overarching transversal need affecting diverse individuals, including (i) patients, (ii) caregivers, and (iii) HCPs. Both literature and expert stakeholders consistently highlight these needs as some of the most significant challenges faced in this field.

#### Patients’ perspective

As indicated throughout, persons living with rare diseases encounter substantial barriers in obtaining accurate and comprehensive information on various aspects of their lives and disease [20, 21, 43, 44, 122–124]. The literature reports on four key areas of information needs, including: (i) general knowledge about the disease, prognosis, and management; (ii) information about social security systems, including legal, financial, and social support; (iii) guidance on family planning, and (iv) details on research opportunities for potential participation [21, 29, 40, 43, 103, 109, 125, 126]. Moreover, during the consultations the availability of research on the disease was mentioned several times as an important need: “*When you are diagnosed with a disease, it might be completely different in terms of psychological impact for the patients if there is a lot of research on your disease or there is no research at all*”. Peer support networks and patient organisations play an important role for providing reliable and comprehensive information, yet studies report that these infrastructures are often insufficiently developed and should be strengthened to empower patients and foster connections among those with similar conditions [29, 43, 103, 125]. The latter was also confirmed during the consultations: “*Today, very few patients are properly guided throughout their disease. There are many gaps that patient organisations try to cover for free and without funding*”.

#### Caregivers’ perspective

Caregivers also face significant information gaps. They struggle in understanding the patients’ condition, managing daily care needs, and navigating the complexities of the healthcare system, especially when it comes to very rare and poorly understood diseases. Caregivers need clear and practical information to support patients, who often feel overwhelmed by the high amount of medical information and numerous decisions they must face [101]. The caregiver’s role becomes even more pivotal when patients are too fatigued, stressed, or experiencing a relapse of the disease, making it essential for caregivers to have reliable and accessible information at every stage of the patient journey [127].

#### HCP’s perspective

HCPs play an important role as primary information sources, but systemic challenges can impede effective communication. Research indicates that patient experiences with HCP-provided information vary greatly depending on the healthcare setting. While specialized centres often deliver high-quality information, non-specialized settings may provide inadequate or overly general information, leaving patients with an incomplete understanding of their condition [34, 122]. Some literature reports that patients sometimes feel more knowledgeable about their conditions than their HCPs, leading them to navigate their healthcare independently and seek supplementary information from online resources, including search engines, communities, and forums [34, 45, 56, 116, 128]. Challenges contributing to HCPs’ knowledge gaps include insufficient documentation on rare diseases (e.g., brochures and information sources), limited medical training (e.g., conferences and webinars), and a lack of hands-on experience with rare disease cases [16, 44–47, 64, 123, 129]. Addressing this information deficit requires increased research and reliable evidence, as well as access to up-to-date clinical practice guidelines tailored to rare diseases to aid in diagnosis, treatment, and integrated healthcare [33, 42, 95, 123, 130].

## Discussion

The insights from this rapid literature review and two stakeholder consultations identified a complex, multifaceted landscape of need criteria to evaluate unmet health-related needs in rare diseases. These criteria span across (i) patient, and (ii) caregiver levels, with (iii) information needs as an overarching transversal need affecting both levels. This study emphasizes the importance of considering health-related needs beyond pharmaceutical needs, including but not limited to the psychological and social burden of the disease, the impact on caregivers, and the lack of information available on many rare diseases, posing challenges for clinicians, researchers, and policymakers. This comprehensive overview provides a foundation for future research aimed at identifying unmet health-related needs within specific disease contexts. Additionally, it offers valuable guidance for decision-makers seeking to optimize healthcare systems, particularly in the context of rare diseases.

While this study does not focus on weighting and prioritizing certain criteria over others, the included criteria encompass the most pressing needs identified in other significant projects and frameworks. For instance, the AWaRDS study in the US, which found that the highest-priority needs for adults with rare disorders included symptom management (e.g. addressing pain, fatigue, and physical changes), daily activity limitations, and treatment complexity and uncertainties [131]. Similarly, Tumiene et al.'s conceptual framework on care pathways in rare diseases also reports prolonged diagnostic odysseys, limited treatment access, and the burden of care coordination, which resonates with the challenges highlighted in our study [132]. The Rare Barometer patient survey by EURORDIS-Rare Diseases Europe further highlights the long and difficult diagnostic journey for many persons living with rare diseases, with 56% receiving a diagnosis over six months after initial medical contact and an average confirmed diagnosis time of nearly five years [133]. Moreover, the Rare2030 foresight study further supports our findings, emphasizing the need for long-term, integrated strategies that prioritize faster, more accurate diagnoses, equitable access to high-quality healthcare, affordable treatments, and multi-stakeholder collaboration [134].

In response to these identified needs, rare disease patient organisations, such as EURORDIS at the European level and RadiOrg (Belgium), VSOP (The Netherlands), ACHSE (Germany), and UNIAMO (Italy) at the national level, play a crucial role in addressing the needs of persons living with rare diseases. These organisations provide peer networks, information resources, and support materials. Furthermore, most disease-specific patient organisations offer specialized services, such as physiotherapy programs, support materials, or assistance with ergonomic home adaptations. However, these resources might not be universally available or accessible to all persons living with rare diseases, resulting in gaps in awareness and support. Additionally, these patient organisations often face limited resources themselves, which restricts their capacity to provide assistance. More and systematic governmental support for patient organisations could address this challenge.

Besides patient organisations, registries could significantly enhance the understanding of patients' needs. However, many existing registries primarily focus on data from clinical trials, often overlooking patient-relevant outcomes such as quality of life, psychological and social health, or caregiver-related data [135–137]. Expanding registries to include these dimensions, while protecting patients’ identities, could better capture the holistic needs of persons living with rare diseases and support decision-making across various levels accordingly [138, 139]. For instance, the Central Registry for Rare Diseases (CRRD), managed by Sciensano in Belgium, is a national initiative that systematically collects data to improve care pathways and better capture patient experiences [140]. Given the small number of patients typically present in one country, it is imperative to gather larger-scale data. Therefore, a database at the EU level could contribute to a better understanding of needs and regional differences, enabling more targeted actions to meet those needs.

While a robust EU-level database for all rare diseases is still lacking, several initiatives have been established to improve diagnostics, treatments, and data sharing for individuals living with rare diseases, indicating a strong opportunity and willingness for collaboration. For instance, projects like the solve-RD initiative aim to advance rare disease diagnostics by promoting technologies such as Whole Exome Sequencing, building on the infrastructure of European Reference Networks (ERNs) and the European Platform on Rare Disease Registration [141, 142]. These networks and infrastructures foster cross-border collaborations, enabling HCPs to share expertise and resources to tackle diagnostic challenges as well as finding the best available treatments [143]. At the European level, various projects focus on enhancing data collection, data sharing, and fostering collaboration across member states to develop innovative solutions for persons living with rare diseases [144–147]. Global platforms, as demonstrated by the Undiagnosed Diseases Network International and European Registries for Rare Endocrine Conditions (EuRRECa), emphasize the importance of standardized data collection to enhance collaborative research efforts [148]. These efforts should leverage commonly adopted codification systems, such as Orphanet nomenclature, to ensure consistency and facilitate collaboration [142].

At the legislative level, the Orphan Drug Regulation of 2000 has accelerated research by incentivizing the development of orphan medicinal products, but its pharmaceutical focus often overlooks broader health, healthcare, and social needs [149]. Similarly, the EU's proposed unmet medical needs definition excludes non-medical needs critical to persons living with rare diseases [150]. The findings of this study advocate for a more holistic approach in health policy, particularly for rare diseases, where accurate diagnosis, multidisciplinary, and non-pharmaceutical interventions are critical to better address the complex and varied needs of persons living with rare diseases. A clear unmet medical needs definition, complemented by a comprehensive framework for systematically identifying broader needs, remains essential to align healthcare with patients’ true needs and priorities and ensure that the diverse challenges faced by persons living with rare diseases are adequately met.

This study systematically identified criteria covering health-related needs in rare diseases, with some criteria specific to rare diseases and others potentially applicable to broader disease contexts. These insights facilitated the evaluation of the KCE NEED framework and its applicability to rare diseases. While many identified needs align with the NEED framework, certain needs are more pronounced in rare diseases, such as the diagnostic and therapeutic odyssey and the lack of expertise, training, and awareness among professionals. The comparison of the insights from this study and the KCE NEED framework is detailed in KCE report 377C4 [9]. Although the framework is currently applied only at the Belgian level, it represents a preliminary effort towards a more holistic identification of health-related needs at the EU level.

### Strengths, limitations, and future recommendations

This study’s strengths lie in its comprehensive approach, integrating both a literature review and stakeholder consultations to capture a nuanced understanding of patients’ and caregivers’ perspectives. The triangulation of methods enhanced the study’s rigor, and the collaborative involvement of multiple researchers across various stages, from literature data screening and extraction to analysis of the consultations, provided diverse interpretations. Research team discussions further enriched the integration of varied viewpoints, reinforcing the study’s robustness.

However, limitations of this study include potential biases in both the literature review and stakeholder consultations. The reliance on published literature may overlook relevant unpublished data on rare diseases, a known issue in rare disease research given small sample sizes and publication bias. The reliance on the pre-existing KCE NEED framework for data extraction may have overly emphasized predefined criteria within the framework, potentially limiting the identification of other relevant criteria that could have emerged independently. Stakeholder input was incorporated to help mitigate this gap, offering additional perspectives that complemented the literature review. Although no patients were included as authors in this study, we conducted stakeholder consultations to validate the findings of the literature. Stakeholder consultations employed convenience sampling; however, expert validation of participant lists ensured the inclusion of key organizations and participants. Nevertheless, this approach may have missed less visible or represented stakeholders such as smaller patient organisations or professional caregivers, limiting inclusivity and diversity. Furthermore, participants’ responses could have been influenced by perceived researcher expectations or the focus on developing a generic framework, potentially introducing response and confirmation biases.

This study did not include a weighting exercise, and the identified needs were not compared or ranked based on their perceived importance. Such an exercise could, however, guide decision-making. The authors believe that patients are best suited to perform this prioritization effort. Future research should focus on conducting this prioritization exercise to better understand the relative importance of different needs. Additionally, the availability of medicines, diagnostics, and expertise centres varies significantly between member states. Consequently, the needs of rare disease patients are expected to vary between EU member states and across continents. This study did not include a comparative assessment, but future research could benefit from such an analysis to better differentiate and address regional needs specifically.

## Conclusion

This study highlights the multifaceted need criteria to evaluate unmet health-related needs of persons living with rare diseases and their caregivers, emphasizing that these needs extend beyond medical interventions to include health, healthcare, and social domains. It emphasizes the importance for healthcare policies to adapt a holistic, patient-centred approach that prioritizes improved psychological health, autonomy, timely diagnosis, accessibility to care, and financial support for patients and their caregivers. The findings call for awareness and policy decisions that recognize the complexity of rare disease challenges, urging investments in diagnostic tools, multidisciplinary care, support systems, and more comprehensive information on rare diseases. By addressing these diverse needs, policymakers can enhance the quality of life for persons living with rare diseases and their caregivers and foster a more inclusive healthcare framework.

## Supplementary Information

Additional file 1.

Additional file 2.

Additional file 3.

Additional file 4.

Additional file 5.

## Data Availability

This study is based on publicly available scientific literature obtained through established online databases. The stakeholder consultations conducted as part of this research involved the collection of personal data, which cannot be shared publicly to ensure compliance with data protection regulations and to safeguard the privacy of participants.

## Abbreviations

EU: European Union
HCP: Healthcare providers
KCE: Belgian Health Care Knowledge Centre
NEED: Need Examination, Evaluation, and Dissemination
R&D: Research & Development
