# Supplementary material for: Criteria to evaluate unmet health-related needs of persons living with rare diseases and their caregivers: rapid literature review and stakeholder consultations
Source: Orphanet J Rare Dis. 2025 Jul 1;20:321. doi: 10.1186/s13023-025-03838-6 (PMC12211369; doi:10.1186/s13023-025-03838-6)
Supplement: Supplementary file 1 — Additional file 1. [file 13023_2025_3838_MOESM1_ESM.pdf]

Supplementary material 1: Search string

Database	Search string	Results
**PubMed**	("Rare Diseases"[Mesh]) OR ("Rare disease*"[Title/Abstract]) OR ("Rare disease"[Tiab:~10]) OR ("Rare diseases"[Tiab:~10]) OR ("Orphan disease*"[Title/Abstract]) OR ("Orphan disease"[Tiab:~10]) OR ("Orphan diseases"[Tiab:~10]) OR ("Rare disorder*"[Title/Abstract]) OR ("Rare disorder"[Tiab:~10]) OR ("Rare disorders"[Tiab:~10]) OR ("Rare condition*"[Title/Abstract]) OR ("Rare condition"[Tiab:~10]) OR ("Rare conditions"[Tiab:~10]) OR ("Orphan condition*"[Title/Abstract]) OR ("Orphan conditions"[Tiab:~10]) OR ("Orphan condition"[Tiab:~10]) OR ("Orphan designation*"[Title/Abstract]) OR ("Orphan designation"[Tiab:~10]) OR ("Orphan designations"[Tiab:~10]) OR (“Orphan disorder”[Tiab:~10]) OR (“orphan disorders”[tiab:~10]) OR (“orphan disorder*”[Tiab])<br>AND<br>("Surveys and Questionnaires"[Mesh]) OR ("survey*"[Tiab]) OR ("questionnaire*"[Tiab]) OR ("Self Report"[Mesh]) OR ("self report”[Tiab:~3]) OR ("self reports”[Tiab:~3]) OR ("Qualitative Research"[Mesh]) OR ("Qualitative research”[Tiab:~3]) OR ("Qualitative study”[Tiab:~3]) OR ("Qualitative studies”[Tiab:~3]) OR ("Quantitative research”[Tiab:~3]) OR ("Quantitative study”[Tiab:~3]) OR ("Quantitative studies”[Tiab:~3]) OR ("Interviews as Topic"[Mesh]) OR ("Interview*"[Tiab]) OR ("Focus Groups"[Mesh]) OR ("Focus group*"[Tiab]) OR ("Focus discussion*"[Tiab]) OR ("Multi criteria decision analysis"[Tiab]) OR ("Multiple criteria decision analysis"[Tiab]) OR ("Multicriteria decision analysis"[Tiab]) OR ("Multi criteria decision making"[Tiab]) OR ("Multiple criteria decision making"[Tiab]) OR ("Multicriteria decision making"[Tiab]) OR ("Multi criteria decision tool*"[Tiab]) OR ("MCDA"[Tiab]) OR ("Registries"[Mesh]) OR ("Registries"[Tiab]) OR ("Registry"[Tiab]) OR ("Register*"[Tiab]) OR ("Workshop*"[Tiab]) OR ("Expert panel”[Tiab:~7]) OR ("Expert panels”[Tiab:~7]) OR ("Stakeholder panel”[Tiab:~7]) OR ("Stakeholder panels”[Tiab:~7]) OR ("Forum*"[Tiab]) OR ("Stakeholder consultation”[Tiab:~7]) OR ("Stakeholder consultations”[Tiab:~7]) OR ("Expert consultation”[Tiab:~7]) OR ("Expert consultations”[Tiab:~7]) OR ("Patient Participation"[Mesh]) OR ("Patient Participation”[Tiab:~7]) OR ("Patient involvement”[Tiab:~7]) OR ("Patient empowerment”[Tiab:~7]) OR ("Patient engagement”[Tiab:~7]) OR ("Patient activation”[Tiab:~7]) OR ("Social network*"[Tiab]) OR ("Engagement method"[Tiab:~7]) OR ("Engagement methods"[Tiab:~7]) OR ("Needs Assessment"[Mesh]) OR ("Needs Assessment”[Tiab:~7]) OR (“assessing needs”[Tiab:~7]) OR ("Need determination”[Tiab:~7]) OR (“determining need”[Tiab:~7]) OR ("Needs determination”[Tiab:~7]) OR (“determining needs”[Tiab:~7]) OR ("Patient Outcome Assessment"[Mesh]) OR ("Patient Outcome Assessment”[Tiab:~7]) OR ("Patient Outcome Assessments”[Tiab:~7]) OR "Epidemiologic Methods"[Mesh] OR ("Epidemiologic Method*"[Tiab]) OR ("Epidemiologic research"[Tiab:~7]) OR ("Epidemiological research"[Tiab:~7]) OR ("Health Services Research"[Mesh]) OR ("Health Services Research”[Tiab:~7]) OR ("Health Services Evaluation”[Tiab:~7]) OR ("Health Services Evaluations”[Tiab:~7]) OR ("Health care research”[Tiab:~7]) OR ("Healthcare research”[Tiab:~7]) OR ("Observational research”[Tiab:~7]) OR ("Observational studies”[Tiab:~7]) OR ("Observational study”[Tiab:~7]) OR ("Cohort research”[Tiab:~7]) OR ("Cohort study”[Tiab:~7]) OR ("Cohort studies”[Tiab:~7]) OR ("case-control study”[Tiab:~7]) OR ("case-control studies”[Tiab:~7]) OR ("healthcare evaluation”[Tiab:~7]) OR ("health care evaluation”[Tiab:~7]) OR ("health-care evaluation”[Tiab:~7]) OR ("visual analogue scale*”[Tiab]) OR ("EQ-5D”[Tiab])<br>AND	1 289

	(("Unmet need"[Tiab:~6]) OR ("Unmet needs"[Tiab:~6]) OR ("Unmet demand"[Tiab:~6]) OR ("Unmet demands"[Tiab:~6]) OR ("Societal need"[Tiab:~6]) OR ("Societal needs"[Tiab:~6]) OR ("Societal demand"[Tiab:~6]) OR ("Societal demands"[Tiab:~6]) OR ("Community need"[Tiab:~6]) OR ("Community needs"[Tiab:~6]) OR ("Community demand"[Tiab:~6]) OR ("Community demands"[Tiab:~6]) OR ("Health need"[Tiab:~6]) OR ("Health needs"[Tiab:~6]) OR ("Health demand"[Tiab:~6]) OR ("Health demands"[Tiab:~6]) OR ("Healthcare need"[Tiab:~6]) OR ("Healthcare needs"[Tiab:~6]) OR ("Healthcare demand"[Tiab:~6]) OR ("Healthcare demands"[Tiab:~6]) OR (“care need”[Tiab:~6]) OR ("care needs"[Tiab:~6]) OR (“care demand”[Tiab:~6]) OR (“care demands”[Tiab:~6]) OR ("Medical need"[Tiab:~6]) OR ("Medical needs"[Tiab:~6]) OR ("Medical demand"[Tiab:~6]) OR ("Medical demands"[Tiab:~6]) OR ("Therapeutic need"[Tiab:~6]) OR ("Therapeutic needs"[Tiab:~6]) OR ("Therapeutic demand"[Tiab:~6]) OR ("Therapeutic demands"[Tiab:~6]) OR ("Therapeutical need"[Tiab:~6]) OR ("Therapeutical needs"[Tiab:~6]) OR ("Therapeutical demand"[Tiab:~6]) OR ("Therapeutical demands"[Tiab:~6]) OR ("Pharmaceutic need"[Tiab:~6]) OR ("Pharmaceutic needs"[Tiab:~6]) OR ("Pharmaceutic demand"[Tiab:~6]) OR ("Pharmaceutic demands"[Tiab:~6]) OR ("Pharmaceutical need"[Tiab:~6]) OR ("Pharmaceutical needs"[Tiab:~6]) OR ("Pharmaceutical demand"[Tiab:~6]) OR ("Pharmaceutical demands"[Tiab:~6]) OR ("Patient need"[Tiab:~6]) OR ("Patient needs"[Tiab:~6]) OR ("Patient demand"[Tiab:~6]) OR ("Patient demands"[Tiab:~6]) OR ("Population need"[Tiab:~6]) OR ("Population needs"[Tiab:~6]) OR ("Population demand"[Tiab:~6]) OR ("Population demands"[Tiab:~6]) OR ("Therapy need"[Tiab:~6]) OR ("Therapy needs"[Tiab:~6]) OR ("Therapy demand"[Tiab:~6]) OR ("Therapy demands"[Tiab:~6]))	
**Embase NOT abstracts**	'rare disease'/exp OR ((‘rare’ OR ‘orphan’) NEAR/11 (‘disease*’ OR ‘disorder*’ OR ‘condition*’ OR ‘designation*’)):ti,ab,kw<br>AND<br>'questionnaire'/exp OR 'questionnaire*':ti,ab,kw OR 'survey*':ti,ab,kw OR 'self report'/exp OR (self NEAR/4 report*):ti,ab,kw OR 'qualitative research'/exp OR (qualitative NEAR/4 research*):ti,ab,kw OR (qualitative NEAR/4 stud*):ti,ab,kw OR 'quantitative study'/exp OR (quantitative NEAR/4 stud*):ti,ab,kw OR (quantitative NEAR/4 research*):ti,ab,kw OR 'interview'/exp OR 'interview*':ti,ab,kw OR 'focus group*':ti,ab,kw OR 'focus discussion*':ti,ab,kw OR 'multicriteria decision analysis'/exp OR 'multicriteria decision analys?s':ti,ab,kw OR 'multi criteria decision analys?s':ti,ab,kw OR 'multiple criteria decision analys?s':ti,ab,kw OR 'multi criteria decision making':ti,ab,kw OR 'multiple criteria decision making':ti,ab,kw OR 'multicriteria decision making':ti,ab,kw OR 'multi criteria decision tool*':ti,ab,kw OR 'MCDA':ti,ab,kw OR 'register'/exp OR 'register':ti,ab,kw OR 'registr*':ti,ab,kw OR 'workshop'/exp OR 'workshop*':ti,ab,kw OR 'patient participation'/exp OR 'patient empowerment'/exp OR 'patient engagement'/exp OR 'social network'/exp OR 'needs assessment'/exp OR 'health services research'/exp OR ((‘Expert*’ OR ‘patient*’ OR ‘expert*’ OR ‘stakeholder*’ OR ‘social’ OR ‘societal’) NEAR/7 (‘panel*’ OR ‘forum*’ OR ‘consult*’ OR ‘participat*’ OR ‘involv*’ OR ‘engag*’ OR ‘network*’ OR ‘activ*’)):ti,ab,kw OR 'needs assessment'/exp OR ((‘need*’ OR ‘patient NEAR/4 outcome’) NEAR/7 (‘assess*’ OR ‘determinat*’)):ti,ab,kw OR ((‘engagement’ OR ‘epidemiologic*’ OR ‘health NEAR/4 service*’ OR ‘health care’ OR ‘healthcare’ OR ‘health-care’ OR ‘observational’ OR ‘cohort*’ OR ‘case-control*’) NEAR/7 (‘method*’ OR ‘research’ OR ‘evaluation*’ OR ‘stud*’)):ti,ab,kw OR ‘visual analogue scale*’:ti,ab,kw OR ‘EQ-5D’:ti,ab,kw<br>AND<br>‘unmet medical need’/exp OR ((‘medical’ OR ‘unmet’ OR ‘societ*’ OR ‘communit*’ OR ‘health*’ OR ‘health-care’ OR ‘care’ OR ‘therapeutic*’ OR ‘Pharmaceutic*’ OR ‘patient*’ OR ‘population*’) NEAR/7 (‘need*’ OR ‘demand*’)):ti,ab,kw	1 929

	NOT<br>‘conference abstract’:it
