# Supplementary material for: Criteria to evaluate unmet health-related needs of persons living with rare diseases and their caregivers: rapid literature review and stakeholder consultations
Source: Orphanet J Rare Dis. 2025 Jul 1;20:321. doi: 10.1186/s13023-025-03838-6 (PMC12211369; doi:10.1186/s13023-025-03838-6)
Supplement: Supplementary file 2 — Additional file 2. [file 13023_2025_3838_MOESM2_ESM.pdf]

Supplementary material 2: Extraction framework

Overarching topics	Identified parameters
**Publication specifications**	• Article title<br>• Author name<br>• Author stakeholder group<br>• Industry funded (yes/no)<br>• Publication type<br>• Geographic region<br>• Overarching geographic region (continental)<br>• Year of publication
**Methodological aspects**	• Primary/meta research<br>• Method category<br>• Broad method category (literature review, qualitative study, quantitative study, or mix-method study)<br>• Method details<br>• Recruitment strategy<br>• Measures/tools<br>• Number of participants<br>• Study participant details<br>• Research stakeholder group (who are the findings about?)<br>• Study participant group (who is being included as participants in the study?)
**Disease specifications**	• Disease category (disease specific or non-disease specific)<br>• Disease name<br>• General disease category<br>• WHO ICD-11 code<br>• Prevalence<br>• Incidence
**Methodological challenges**	**Challenges related to the study design**<br>• Estimation of patients' health status<br>• Profile of the included participant (sample bias)<br>• Challenges related to the method used<br>• Limited clinical knowledge<br>• Duration of the study<br>**Challenges related to recruitment and study sample**<br>• Sample size<br>• Sample variability<br>• Generalisability/representativeness<br>• Underdiagnosis<br>**Challenges related to the data source**<br>• Limited (comparable) data sources and limited infrastructure<br>• Completeness of the data<br>• Validity of the data<br>• Challenges in healthcare systems regarding rare diseases<br>**Other**<br>• Limitations related to clinical trials<br>• Limitations related to differences in healthcare systems<br>• Other
**NEED criteria - Patient needs**	**Health**<br>• Physical<br>• Psychological

	• Autonomy<br>• Mortality
	**Healthcare**
	• Treatment<br>• Patient experience with healthcare professional<br>• Information needs<br>• Accessibility to care<br>• Diagnosis
	**Social**
	• Social support<br>• Impact on work<br>• Financial consequences
**NEED criteria – Societal needs**	**Health**
	• Transmissibility<br>• Preventability<br>• Burden of disease
	**Healthcare**
	• Economic burden
	**Social**
	• Environmental impact
**NEED criteria – Future needs**	**Health**
	• Future public health trends<br>• Equity
**Other**	• Other<br>• Author suggestions/recommendations<br>• Reviewer comments
