# Supplementary material for: Criteria to evaluate unmet health-related needs of persons living with rare diseases and their caregivers: rapid literature review and stakeholder consultations
Source: Orphanet J Rare Dis. 2025 Jul 1;20:321. doi: 10.1186/s13023-025-03838-6 (PMC12211369; doi:10.1186/s13023-025-03838-6)
Supplement: Supplementary file 3 — Additional file 3. [file 13023_2025_3838_MOESM3_ESM.pdf]

Default Question Block

Dear participant,

Thank you for taking part in the **first workshop** organised by the Belgian Healthcare Knowledge Centre (KCE) and KU Leuven as part of a study titled: "Applicability of the NEED framework to assess patient and societal needs in patients suffering from rare diseases." This study is being conducted in the context of the NEED (Needs Examination, Evaluation and Dissemination) project.

The main aims of this project are to:

1. Understand your perspectives on the proposed need criteria and the associated measurement methods and its applicability to rare diseases
2. Assess how the evidence on rare diseases collected according to the NEED framework can be used by decision-makers (is there an added value compared to the current approach?)
3. Assess whether/how the framework and/or procedures need to be adapted to be applicable to rare diseases

In preparation of the first workshop, concerning the first aim of this project, you are kindly invited to participate in this online survey.

What is asked from me and how long does it take to complete the survey?
We would like to have your input and opinion about the following aspects:

- In part 1, we would like to collect some background information about yourself: your name and organisation (optional) and which stakeholder group you represent.
- In part 2, we would like to ask your personal opinion on: each criterion and sub-criterion of the NEED framework, whether it fits for needs assessment in rare diseases and whether there is a need to add or adapt certain (sub-)criteria for this specific context. Besides that, we would like your personal opinion on the

proposed methodology to collect evidence for each criterion in case of rare diseases.

This survey will take approximately 20 minutes to complete.

How will my data be handled?
The data collected in this survey will be analysed and used to inform subsequent multistakeholder workshops. We present the aggregated data in the workshops anonymously. However, we may contact you personally following the survey for further information.

Informed consent

To participate in this survey, you must provide your informed consent. Therefore, it is important that you understand and agree with the statements below:

- I have had the opportunity to read the information sheet of the study which aims to determine the applicability of the framework, i.e. the criteria and methods, to assess patient and societal needs in patients suffering from rare diseases;
- I have had the opportunity to ask questions or discuss any concerns regarding the information of this study;
- I was given sufficient time to decide whether I was willing to participate in this study;
- I am aware that participation in this study is completely voluntary;
- I am aware that this questionnaire has been prepared in collaboration with the Belgian Healthcare Knowledge Centre (KCE), as stated in the information sheet;
- I am aware that I can withdraw from this study at any time;
- I understand that the data resulting from this research will be kept for a maximum of 25 years after the end of the research;
- I understand that my data may be used for future research;
- I am aware that the results of this research will be published in scientific journals and disseminated through presentations at conferences or meetings. I understand that these results are based on the analysis of anonymous data and cannot identify me in any way.
- I understand that my data will be processed in accordance with the European General Data Protection Regulation (GDPR).

Do you agree with the above statements?

- ◯ I agree to all the statements and confirm my participation
- ◯ I do not agree to all the statements and do not confirm my participation

In case something is unclear, or you would like additional information, please feel free to contact Zilke Claessens (zilke.claessens@kuleuven.be)

Background information

BACKGROUND INFORMATION

What is the NEED framework and criteria?

The KCE NEED ('Needs Examination, Evaluation and Dissemination') project aims to develop a **NEED framework** (conceptual approach) to collect evidence for particular **health issues** or conditions on unmet health-related needs. Health-related needs can include "***health needs***", "***healthcare needs***", and "***social needs***" ("non-health(care) needs"). The evidence will be collected from a **patient**, **societal** and **future generations perspective**, in a database accessible to all relevant stakeholders.

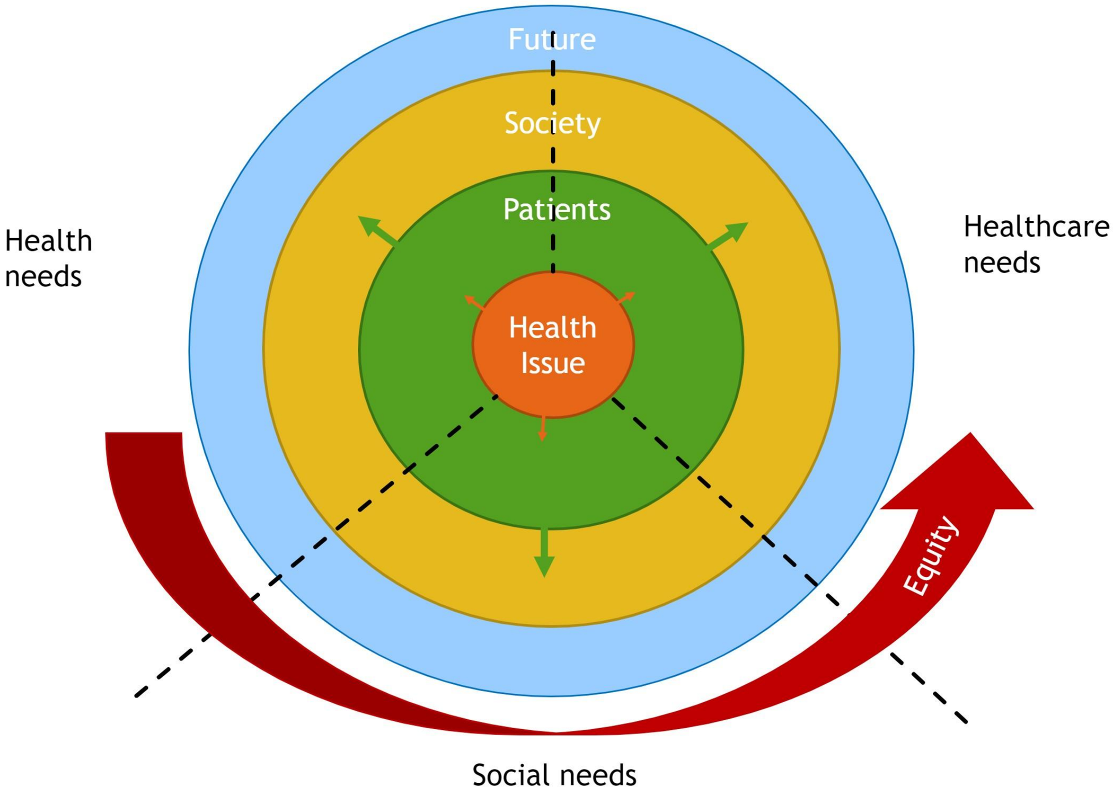

The NEED framework consists of three main **dimension** levels, being the **impact on patients**, **impact on society** and **future impact**. Every dimension consists of explicit health-related need **criteria** to collect evidence on the health issue. These criteria are factors that are most responsible for the high burden of the health issue and that are linked to the impact of a health issue on patients and on the broader society, both in the present and in the future. Every criterion consists of at least one but more often several **sub-criteria**.

- (Sub-)criteria linked to the dimension **impact on patients** can be found here (flowchart).
- (Sub-)criteria linked to the dimension **impact on society** can be found here (flowchart).
- (Sub-)criteria linked to the dimension **future impact** can be found here (flowchart).

The evidence on unmet health-related needs will be collected based on the criteria, making use of particular **methods**. Several types of methods are used in the NEED project, such as qualitative methods (interviews, expert panels), quantitative methods (patient surveys), database sources (BeBoD, Farmanet,...) or literature reviews.

In case something is unclear, or you would like additional information, please feel free

to contact Zilke Claessens (zilke.claessens@kuleuven.be).

Your answers are saved automatically by clicking on the blue arrow at the bottom of each page and submitted automatically when closing your browser.

Important: when a word is underlined with a dotted line, you can hover over the word with your mouse to get additional information or definitions.

Survey

SURVEY

Part 1: General information

Name (optional):

[ ]

Organisation (optional):

[ ]

Which stakeholder category do you represent?

- ○ Regulator
- ○ Policymaker
- ○ HTA body / payer
- ○ Pharmaceutical industry / trade organisation
- ○ Research organisation
- ○ Healthcare professional
- ○ Patient organisation
- ○ Other: [ ]

In case something is unclear, or you would like additional information, please feel free to contact Zilke Claessens (zilke.claessens@kuleuven.be)

Considerations for the application of the NEED framework and its criteria

Part 2: Considerations for the application of the NEED framework and its criteria in the context of rare diseases - IMPACT ON PATIENTS

As mentioned before, the NEEDs framework consists of three dimensions from which a health issue can be approached: the impact it has on the **patients**, the **society** and the **future**. Each of these dimensions consists of specific criteria that can characterize the needs and can be used to measure them. The criteria are also distributed over 3 levels: criteria generating **health needs**, criteria generating **healthcare needs** and criteria generating **social needs**.

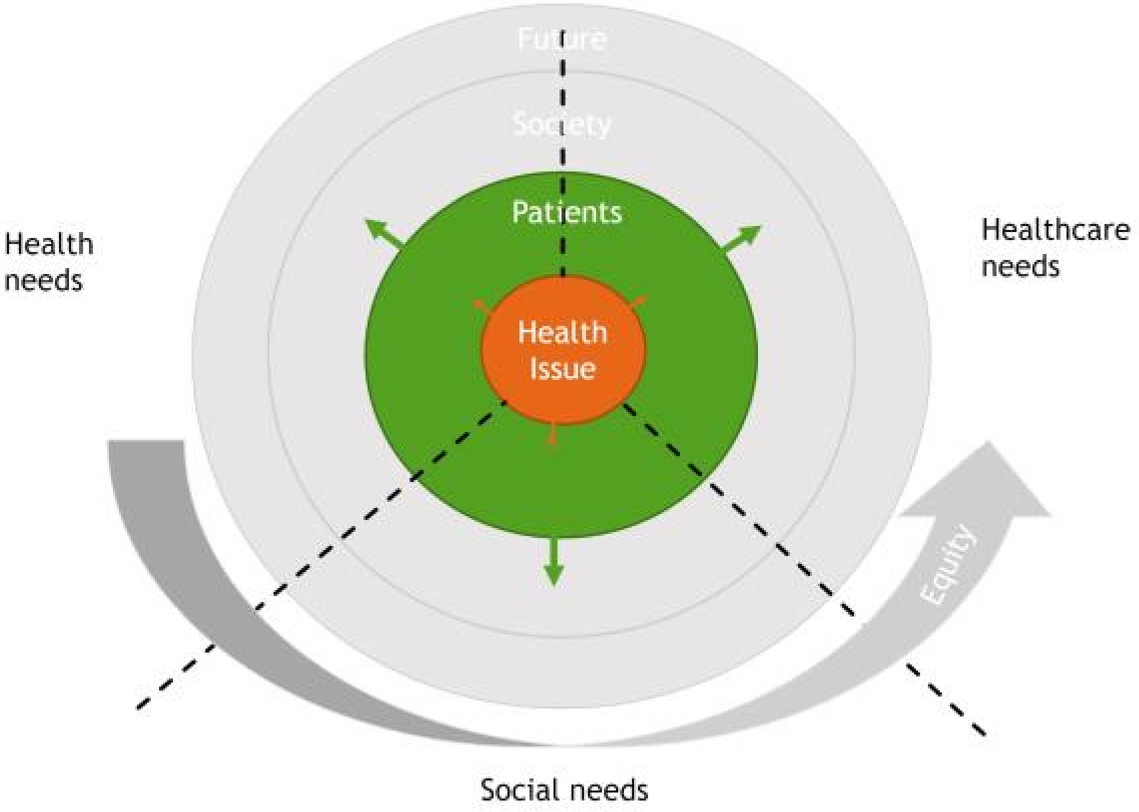

The patient needs criteria are initially measured at the individual patient level and then aggregated over the entire patient population, by taking into account a measure of

disease frequency (i.e. prevalence or incidence).

For the first cluster of questions, we will be focussing on the dimension: **impact on patients**. We kindly invite you to take a look at the different criteria within this first dimension, layed out in the flow chart below. At a later point in this survey we will further zoom in on the sub-criteria and the methods used to measure them.

The **criteria** used to measure the **impact of a health issue on patients** are shown in the figure below:

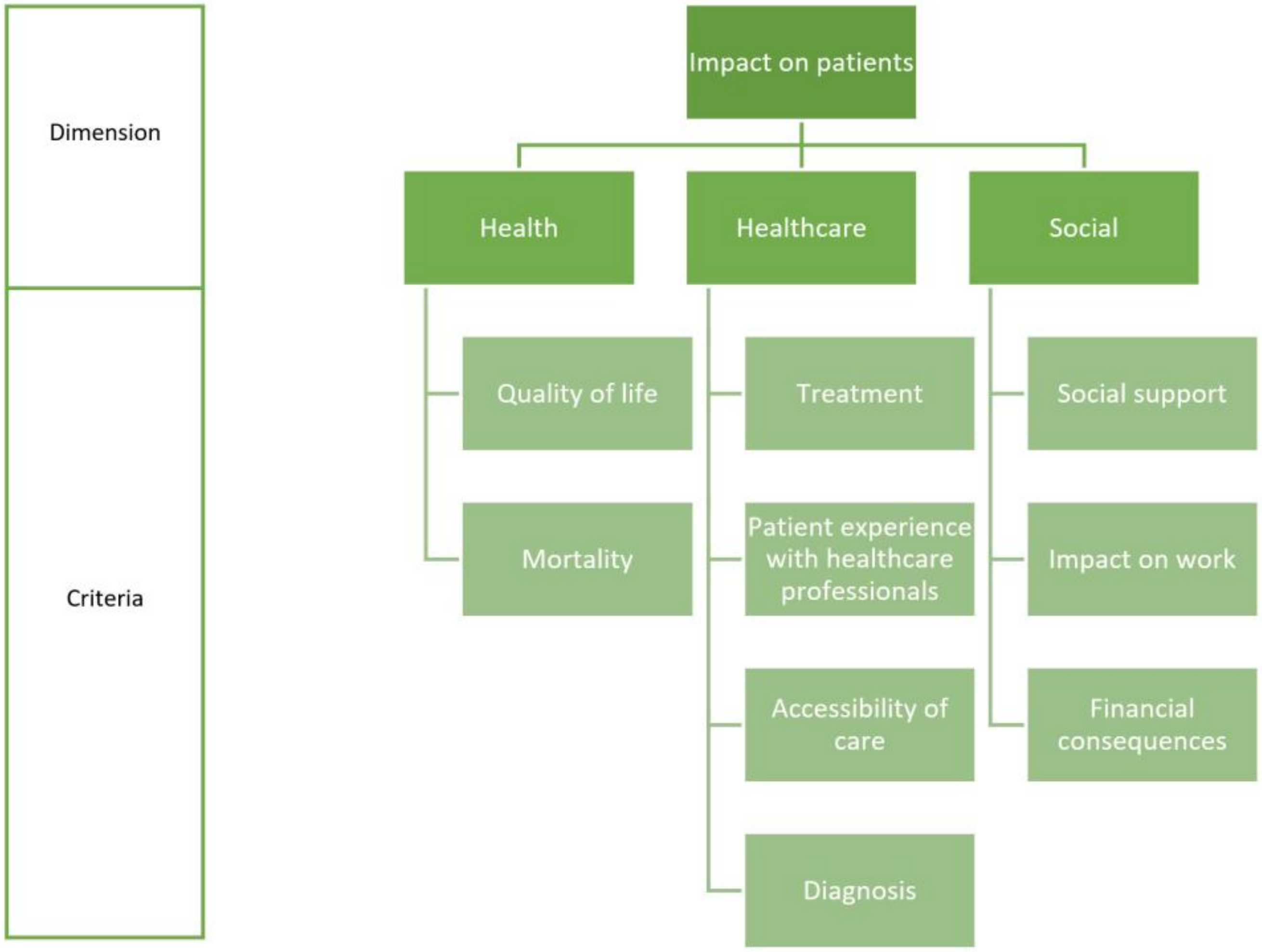

Do you have any comments or suggestions related to the criteria of the dimension 'Impact on patients' in the context of rare diseases?

Do you believe that one or more criteria should be added, adapted or removed in the context of rare diseases?

- Yes, the criteria that should be added/adapted/removed and the reasons why are the following:
- No
- I do not know / I am not competent to answer this question

In case something is unclear, or you would like additional information, please feel free to contact Zilke Claessens (zilke.claessens@kuleuven.be)

Part 2: Considerations for the application of the NEED framework and its criteria in the context of rare diseases - IMPACT ON PATIENTS

We will now look further into the **subcriteria** associated with each criterion. We will first focus on the sub-criteria used to measure **patient HEALTH needs**.

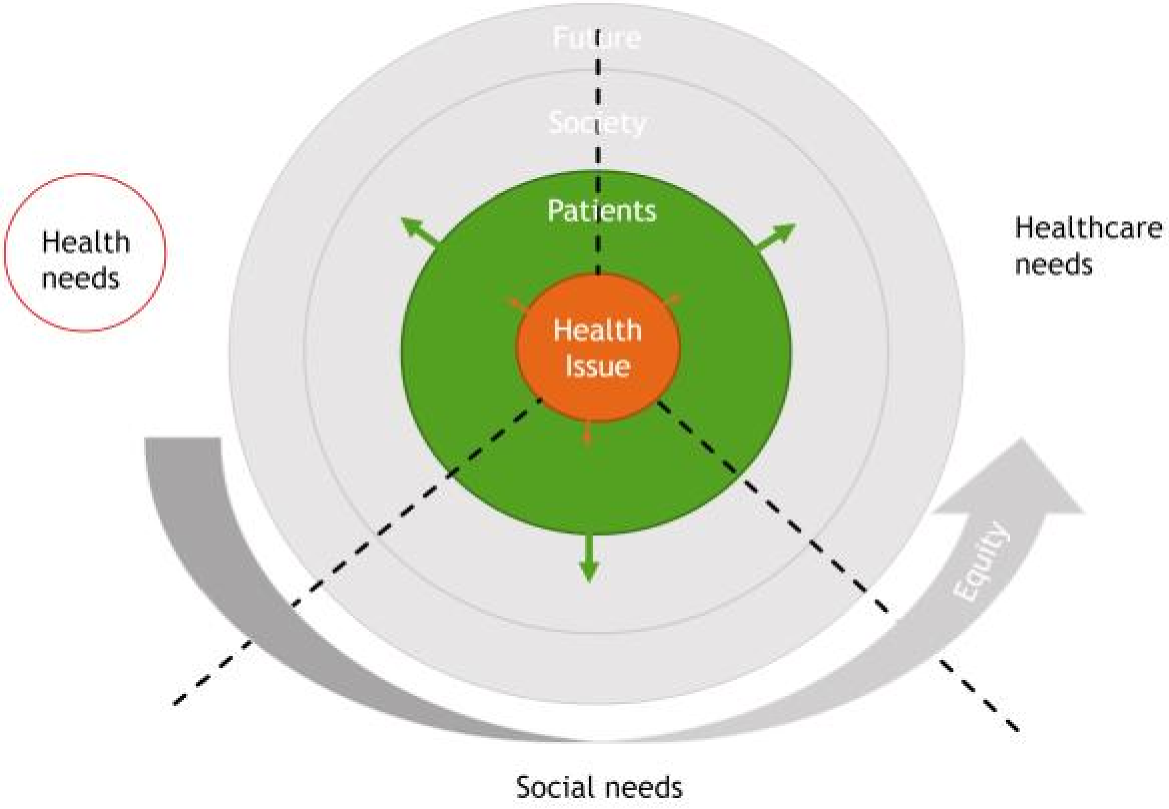

For the following set of questions, we kindly invite you to take a look at the sub-criteria, layed out in the flowchart below.

The **(sub-)criteria** are represented in the figure below:

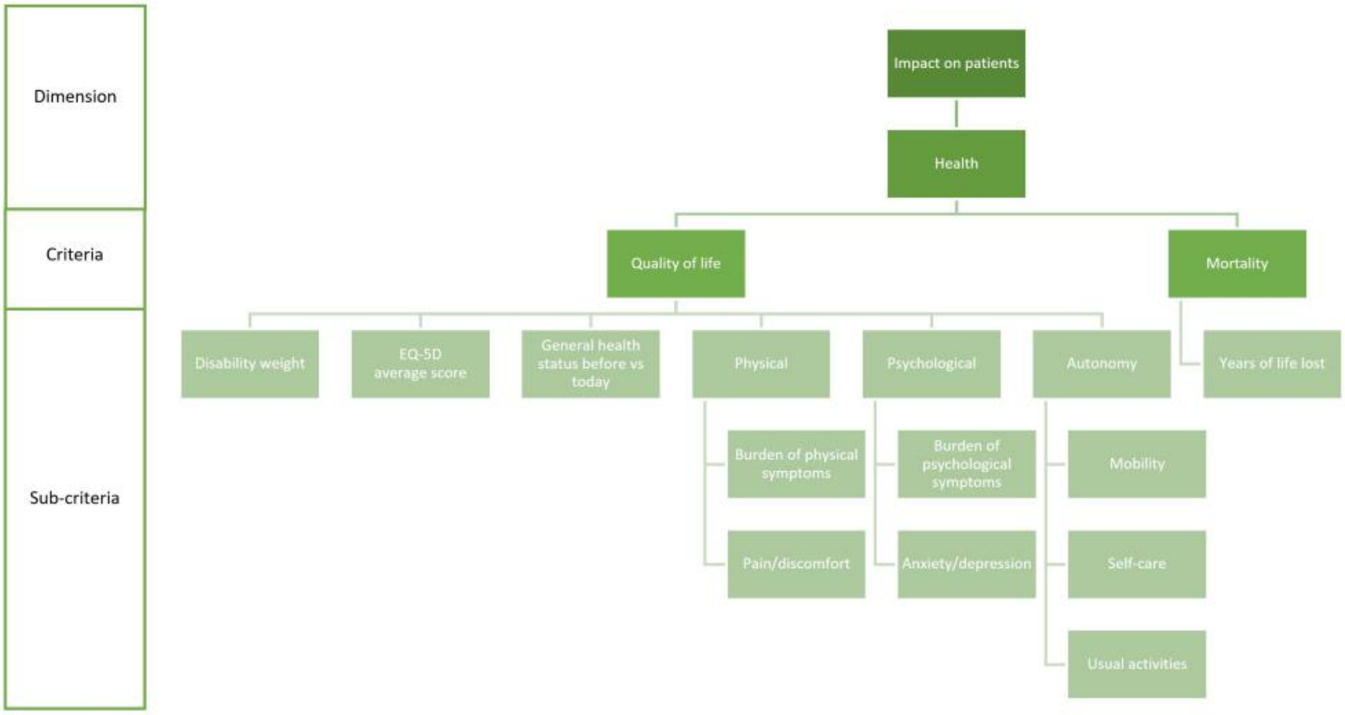

Quality of life (QoL)

The first criterion, within the dimension 'impact on patients, generating health needs' is Quality of Life. The criterion 'Quality of Life' measures the extent to which a health issue impacts the general health, physical health, psychological health, and autonomy of patients.

Do you believe that one or more sub-criteria should be added, adapted or removed to assess the needs related to Quality of Life in the context of rare diseases?

- ◯ Yes, the sub-criteria that should be added/adapted/removed and the reasons why are the following: [ ]
- ◯ No
- ◯ I do not know / I am not competent to answer this question

Do you have any additional concerns or suggestions related to this criterion or sub-criteria in relation to rare diseases?

- ◯ Yes [ ]
- ◯ No

In this table all the sub-criteria of Quality of Life (QoL) are listed. Please indicate to what extent you agree with the proposed method(s) being used to measure the sub-criterion.

	Strongly disagree	Disagree	Slightly disagree	Slightly agree	Agree	Strongly agree	I have insufficient knowledge
**Disability weight**							
Belgian Burden of Disease Study (BeBoD)	◯	◯	◯	◯	◯	◯	◯
**Average score of EQ-5D**							
Survey: Visual Analog Scale (VAS) of EQ-5D	◯	◯	◯	◯	◯	◯	◯
**General health status before vs**							

	Strongly disagree	Disagree	Slightly disagree	Slightly agree	Agree	Strongly agree	I have insufficient knowledge
today							
Survey: Visual Analog Scale (VAS) of EQ-5D	○	○	○	○	○	○	○
Physical health status							
Survey question for experienced burden of physical symptoms related to the health issue	○	○	○	○	○	○	○
Survey: EQ-5D question on pain/discomfort before vs today	○	○	○	○	○	○	○
Psychological health status							
Survey question for experienced burden of psychological symptoms related to the health issue	○	○	○	○	○	○	○
Survey: EQ-5D question on anxiety/depression before vs today	○	○	○	○	○	○	○
Autonomy							
Survey: EQ-5D question on mobility, self-care and usual activities before vs today	○	○	○	○	○	○	○

Do you have any suggestions, alternatives or challenges regarding the method(s) used to measure the sub-criteria that you want to share? (If you propose an alternative method, please provide also a reference)

Mortality

The second criterion, within the dimension 'impact on patients, generating health needs' is Mortality. The criterion "Mortality" measures the impact a health issue has on the life expectancy of patients.
The only **sub-criterion** of mortality is 'Years of life lost'.

Do you believe that one or more sub-criteria should be added, adapted or removed to assess the needs related to mortality in the context of rare diseases?

- Yes, the sub-criteria that should be added/adapted/removed and the reasons why are the following:
- No
- I do not know / I am not competent to answer this question

Do you have any additional concerns or suggestions related to this criterion or sub-criterion in relation to rare diseases?

- Yes
- No

In this table the sub-criterion of mortality is represented. Please indicate to what extent you agree with the proposed method being used to measure the sub-criterion.

	Strongly disagree	Disagree	Slightly disagree	Slightly agree	Agree	Strongly agree	I have insufficient knowledge
Years of life lost<br>Belgian Burden of Disease Study	○	○	○	○	○	○	○

	Strongly disagree	Disagree	Slightly disagree	Slightly agree	Agree	Strongly agree	I have insufficient knowledge
(BeBoD)							

Do you have any suggestions, alternatives or challenges regarding the method(s) used to measure the sub-criterion that you want to share? (If you propose an alternative method, please provide also a reference)

For most sub-criteria, a literature review is performed alongside the other methods (e.g. survey). Do you agree with this? If not, please specify why.

- Yes
- No
- I do not know

In case something is unclear, or you would like additional information, please feel free to contact Zilke Claessens (zilke.claessens@kuleuven.be)

Part 2: Considerations for the application of the NEED framework and its criteria in the context of rare diseases - IMPACT ON PATIENTS

Secondly, the focus is on the sub-criteria used to measure **patient** **HEALTHCARE needs:**

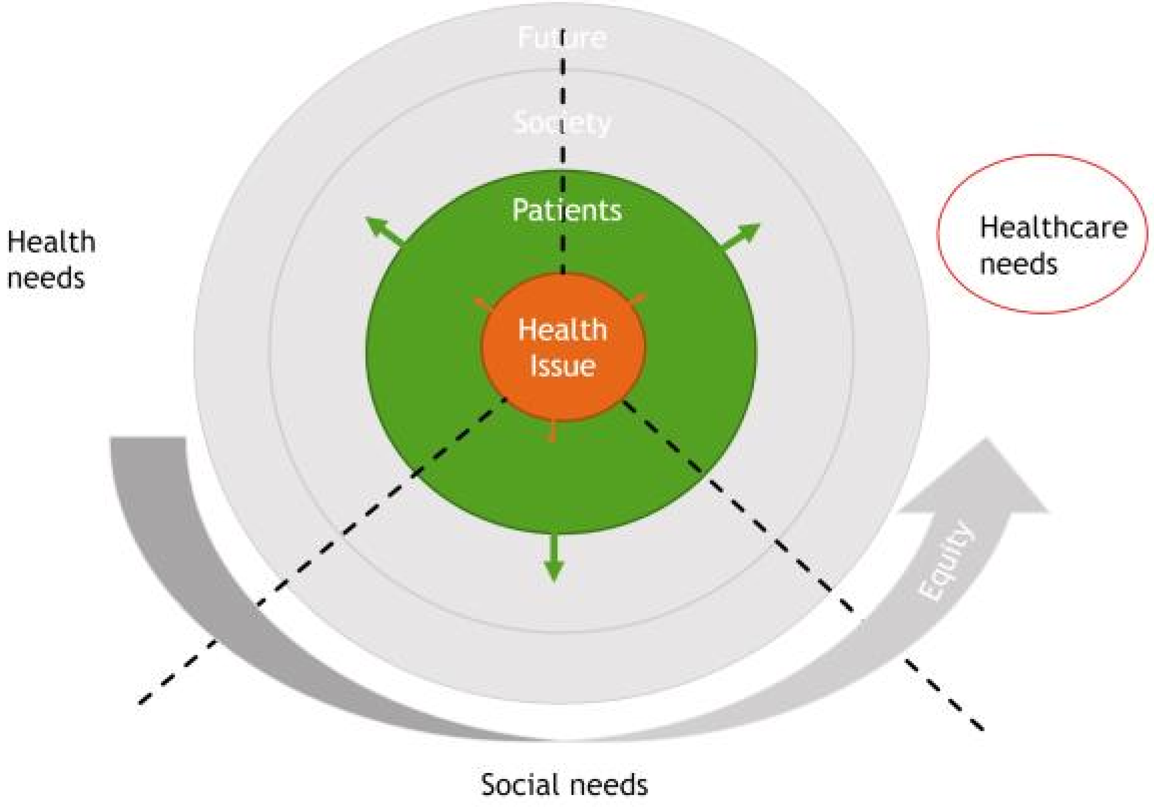

For the following set of questions, we kindly invite you to take a look at the sub-criteria within the dimension **impact on patients**, generating **healthcare needs**, presented in the flowchart below.

The (sub-)criteria are represented in the figure below:

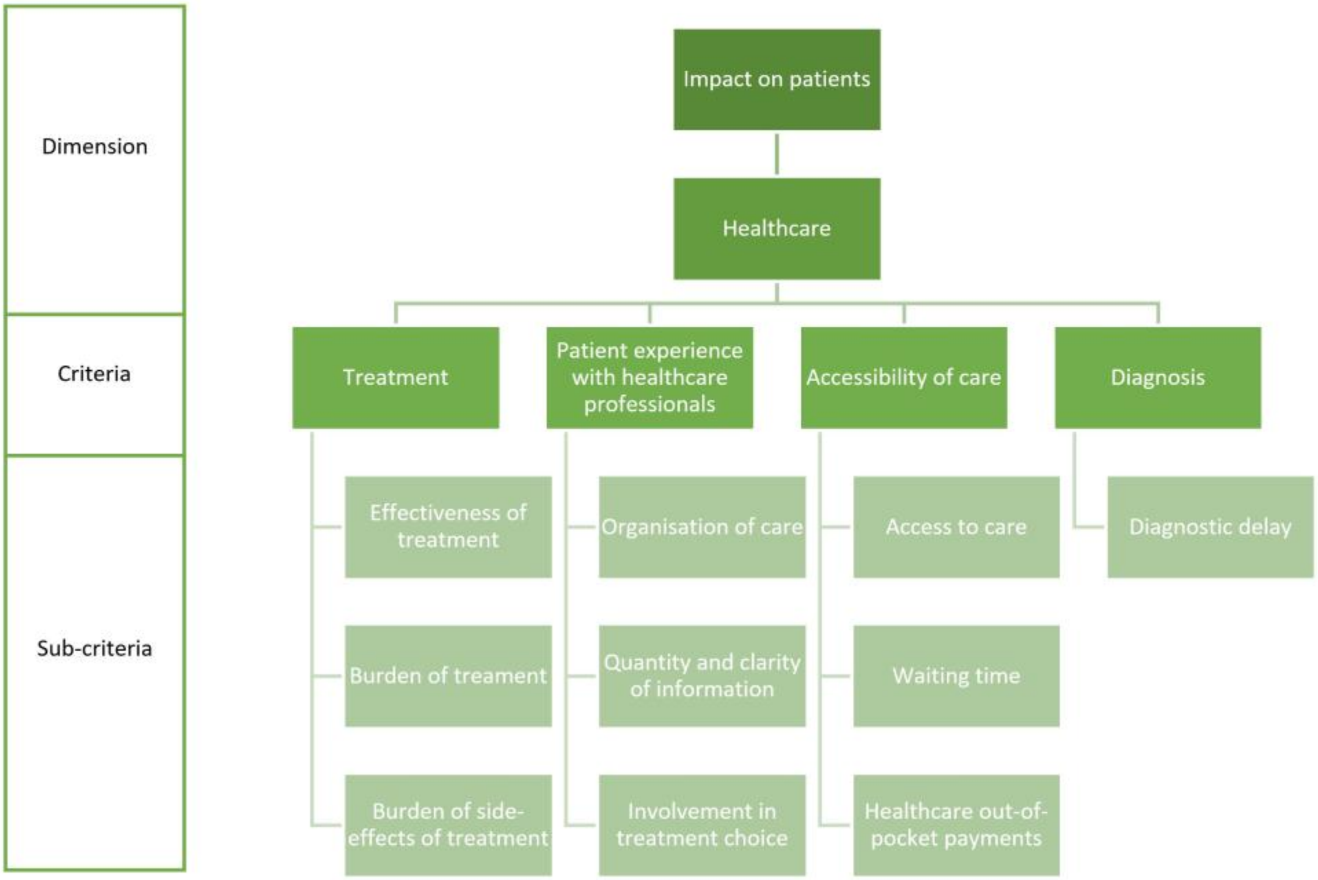

Treatment

The first criterion, within the dimension 'impact on patients, generating healthcare needs' is Treatment. The criterion 'Treatment' measures the extent to which the currently available treatment(s) is/are effective and relieving for patients.

Do you believe that one or more sub-criteria should be added, adapted or removed to assess the needs related to treatment in the context of rare diseases?

- ◯ Yes, the sub-criteria that should be added/adapted/removed and the reasons why are the following: _____
- ◯ No
- ◯ I do not know / I am not competent to answer this question

Do you have any additional concerns or suggestions related to this criterion or sub-criteria in relation to rare diseases?

- ◯ Yes _____

○ No

In this table all the sub-criteria of treatment are listed. Please indicate to what extent you agree with the proposed method(s) being used to measure the sub-criterion.

	Strongly disagree	Disagree	Slightly disagree	Slightly agree	Agree	Strongly agree	I have insufficient knowledge
Effectiveness of each treatment							
Literature review	○	○	○	○	○	○	○
Experienced burden of each treatment							
Survey question	○	○	○	○	○	○	○
Experienced burden of side-effects of each treatment							
Survey question	○	○	○	○	○	○	○

Do you have any suggestions, alternatives or challenges regarding the method(s) used to measure the sub-criteria that you want to share? (If you propose an alternative method, please provide also a reference)

Patient experience with healthcare professionals

The second criterion, within the dimension 'impact on patients, generating healthcare needs' is Patient experience with healthcare professionals. The criterion 'Patient experience with healthcare professionals' measures the extent to which patients' experience with health professionals is appropriate.

Do you believe that one or more sub-criteria should be added, adapted or removed to assess the needs related to patient experience with healthcare professionals in the

context of rare diseases?

- Yes, the sub-criteria that should be added/adapted/removed and the reasons why are the following: ______
- No
- I do not know / I am not competent to answer this question

Do you have any additional concerns or suggestions related to this criterion or sub-criteria in relation to rare diseases?

- Yes ______
- No

In this table all the sub-criteria of patient experience with healthcare professionals are listed. Please indicate to what extent you agree with the proposed method(s) being used to measure the sub-criterion.

	Strongly disagree	Disagree	Slightly disagree	Slightly agree	Agree	Strongly agree	I have insufficient knowledge
**Experience with the organisation of care**							
**Survey question**	O	O	O	O	O	O	O
**Quantity and clarity of the information received**							
**Survey question**	O	O	O	O	O	O	O
**Involvement in treatment choice**							
**Survey question**	O	O	O	O	O	O	O

Do you have any suggestions, alternatives or challenges regarding the method(s) used to measure the sub-criteria that you want to share? (If you propose an alternative method, please provide also a reference)

Accessibility of care

The third criterion, within the dimension 'impact on patients, generating healthcare needs' is Accessibility of care. The criterion 'Accessibility of care' measures the ease with which patients accessed the care they needed, including waiting times and costs.

Do you believe that one or more sub-criteria should be added, adapted or removed to assess the needs related to accessibility of care in the context of rare diseases?

- ○ Yes, the sub-criteria that should be added/adapted/removed and the reasons why are the following: ______
- ○ No
- ○ I do not know / I am not competent to answer this question

Do you have any additional concerns or suggestions related to this criterion or sub-criteria in relation to rare diseases?

- ○ Yes ______
- ○ No

In this table all the sub-criteria of accessibility of care are listed. Please indicate to what extent you agree with the proposed method(s) being used to measure the sub-criterion.

	Strongly disagree	Disagree	Slightly disagree	Slightly agree	Agree	Strongly agree	I have insufficient knowledge
**Access to care**							
**Survey question**	○	○	○	○	○	○	○
**Waiting time**							
**Survey question**	○	○	○	○	○	○	○
**Healthcare out-of-pocket**							

	Strongly disagree	Disagree	Slightly disagree	Slightly agree	Agree	Strongly agree	I have insufficient knowledge
payments							
Different databases	○	○	○	○	○	○	○

Do you have any suggestions, alternatives or challenges regarding the method(s) used to measure the sub-criteria that you want to share? (If you propose an alternative method, please provide also a reference)

Diagnosis

The fourth criterion, within the dimension 'impact on patients, generating healthcare needs' is Diagnosis. The criterion 'Diagnosis' measures the extent to which the diagnosis of the health issue is timely.

Do you believe that one or more sub-criteria should be added, adapted or removed to assess the needs related to diagnosis in the context of rare diseases?

- ○ Yes, the sub-criteria that should be added/adapted/removed and the reasons why are the following:
- ○ No
- ○ I do not know / I am not competent to answer this question

Do you have any additional concerns or suggestions related to this criterion or sub-criteria in relation to rare diseases?

- ○ Yes
- ○ No

In this table the sub-criterion of diagnosis is represented. Please indicate to what extent you agree with the proposed method being used to measure the sub-criterion.

	Strongly disagree	Disagree	Slightly disagree	Slightly agree	Agree	Strongly agree	I have insufficient knowledge
Diagnostic delay							
Survey question	○	○	○	○	○	○	○

Do you have any suggestions, alternatives or challenges regarding the method(s) used to measure the sub-criteria that you want to share? (If you propose an alternative method, please provide also a reference)

For most sub-criteria, a literature review is performed alongside the other methods (e.g. survey). Do you agree with this? If not, please specify why.

- ○ Yes
- ○ No
- ○ I do not know

In case something is unclear, or you would like additional information, please feel free to contact Zilke Claessens (zilke.claessens@kuleuven.be)

Part 2: Considerations for the application of the NEED framework and its criteria in the context of rare diseases - IMPACT ON PATIENTS

Thirdly, the focus is on the sub-criteria of the criteria used to measure **patient SOCIAL needs:**

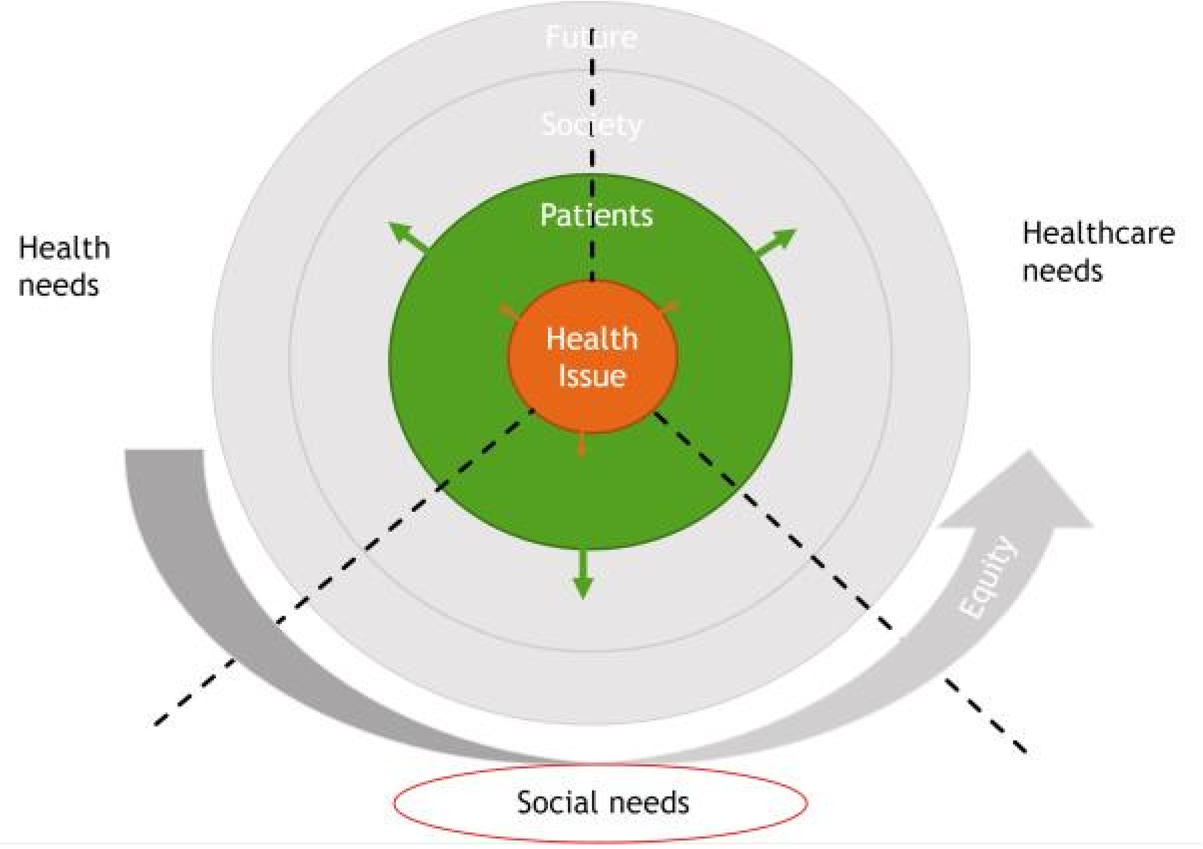

For the following set of questions, we kindly invite you to take a look at the sub-criteria, layed out in the flowchart below:

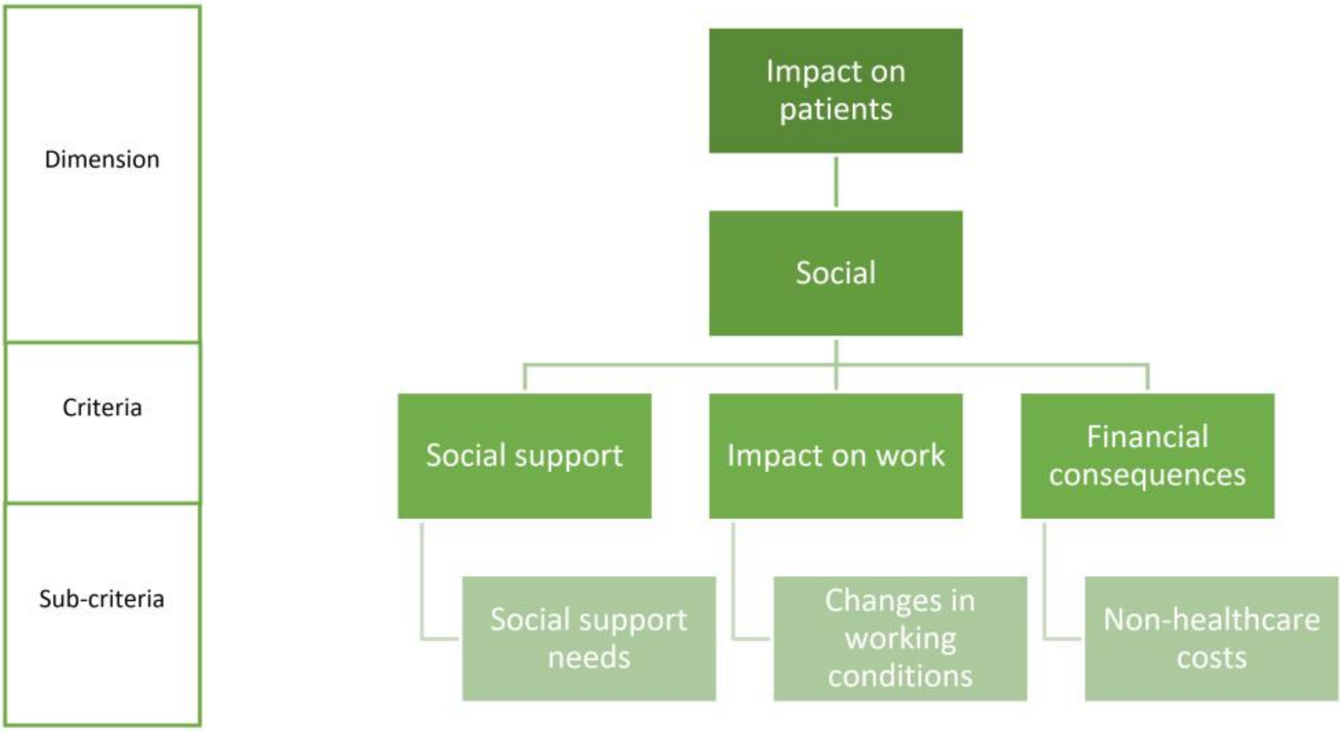

Social support

The first criterion, within the dimension 'impact on patients, generating social needs' is Social support. The criterion 'Social support' measures the needs related to social interactions with the community or family and building relationships.

Do you believe that one or more sub-criteria should be added, adapted or removed to assess the needs related to social support in the context of rare diseases?

- Yes, the sub-criteria that should be added/adapted/removed and the reasons why are the following: ______
- No
- I do not know / I am not competent to answer this question

Do you have any additional concerns or suggestions related to this criterion or sub-criteria in relation to rare diseases?

- Yes ______
- No

In this table the sub-criterion of social support is represented. Please indicate to what extent you agree with the proposed method being used to measure the sub-criterion.

	Strongly disagree	Disagree	Slightly disagree	Slightly agree	Agree	Strongly agree	I have insufficient knowledge
**Social support needs (incl. spiritual needs)**							
**Survey question**	○	○	○	○	○	○	○

Do you have any suggestions, alternatives or challenges regarding the method(s) used to measure the sub-criteria that you want to share? (If you propose an alternative method, please provide also a reference)

Impact on work

The second criterion, within the dimension 'impact on patients, generating social needs' is Impact on work. The criterion 'Impact on work' measures the impact of the health issue on patients' working conditions.

Do you believe that one or more sub-criteria should be added, adapted or removed to assess the needs related to impact on work in the context of rare diseases?

- ○ Yes, the sub-criteria that should be added/adapted/removed and the reasons why are the following: ______
- ○ No
- ○ I do not know / I am not competent to answer this question

Do you have any additional concerns or suggestions related to this criterion or sub-criteria in relation to rare diseases?

- ○ Yes ______
- ○ No

In this table the sub-criterion of impact on work is represented. Please indicate to what extent you agree with the proposed methods being used to measure the sub-criterion.

	Strongly disagree	Disagree	Slightly disagree	Slightly agree	Agree	Strongly agree	I have insufficient knowledge
**Changes in working conditions**							
**Survey question**	○	○	○	○	○	○	○
**Interview**	○	○	○	○	○	○	○

Do you have any suggestions, alternatives or challenges regarding the method(s) used to measure the sub-criteria that you want to share? (If you propose an alternative method, please provide also a reference)

Financial consequences

The third criterion, within the dimension 'impact on patients, generating social needs' is Financial consequences. The criterion 'Financial consequences' measures the additional costs for patients in accessing treatment and care for their health issue.

Do you believe that one or more sub-criteria should be added, adapted or removed to assess the needs related to financial consequences in the context of rare diseases?

- ○ Yes, the sub-criteria that should be added/adapted/removed and the reasons why are the following: ______
- ○ No
- ○ I do not know / I am not competent to answer this question

Do you have any additional concerns or suggestions related to this criterion or sub-criteria in relation to rare diseases?

- ○ Yes ______
- ○ No

In this table the sub-criterion of financial consequences is represented. Please indicate to what extent you agree with the proposed method being used to measure the sub-criterion.

	Strongly disagree	Disagree	Slightly disagree	Slightly agree	Agree	Strongly agree	I have insufficient knowledge
Non-healthcare costs							
Interview	○	○	○	○	○	○	○

Do you have any suggestions, alternatives or challenges regarding the method(s) used to measure the sub-criteria that you want to share? (If you propose an

alternative method, please provide also a reference)

For most sub-criteria, a literature review is performed alongside the other methods (e.g. survey). Do you agree with this? If not, please specify why.

- Yes
- No
- I do not know

Do you have any **other suggestions** regarding certain **patient needs criteria or sub-criteria** that should be **added, removed or adapted** to make the framework fit for the assessment of needs in **rare diseases**?

In case something is unclear, or you would like additional information, please feel free to contact Zilke Claessens (zilke.claessens@kuleuven.be)

Part 2: Considerations for the application of the NEED framework and its criteria in the context of rare diseases - IMPACT ON SOCIETY

Now, we will zoom in on the **second dimension** and its criteria: **impact on society**

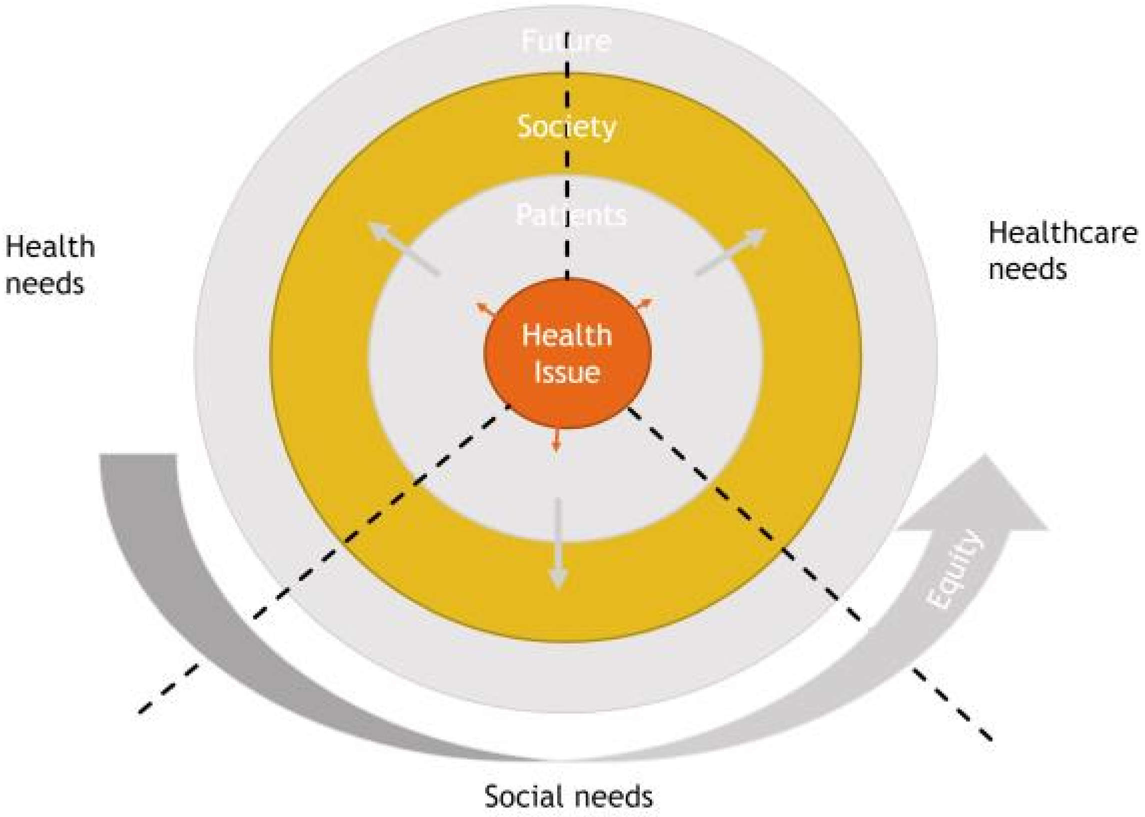

The **criteria** used to measure the **impact of a health issue on society** are shown in the figure below:

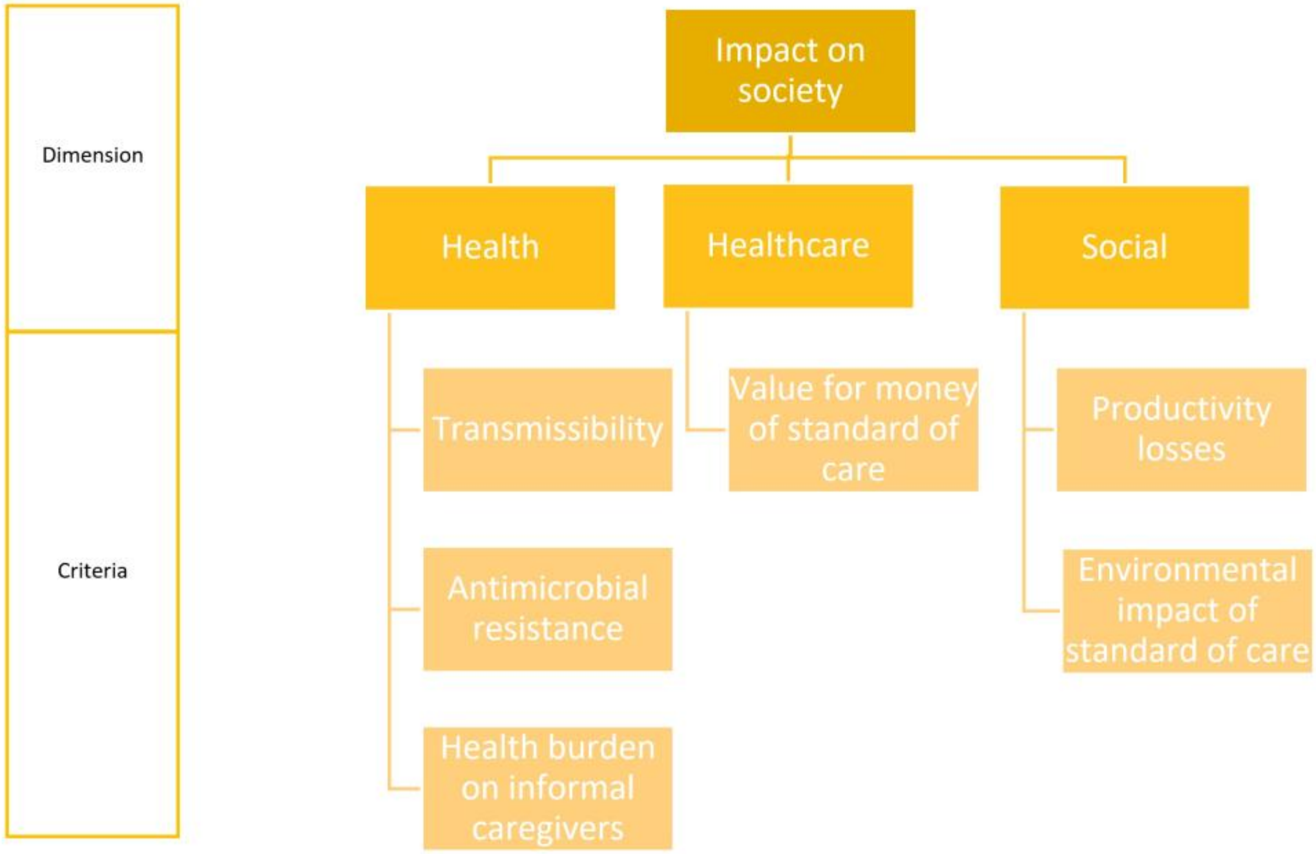

Do you have any comments related to the criteria of the dimension 'Impact on society' in the context of rare diseases?

Do you believe that one or more criteria should be added, adapted or removed in the context of rare diseases?

- Yes, the criteria that should be added/adapted/removed and the reasons why are the following:
- No
- I do not know / I am not competent to answer this question

In case something is unclear, or you would like additional information, please feel free to contact Zilke Claessens (zilke.claessens@kuleuven.be)

Part 2: Considerations for the application of the NEED framework and its criteria in the context of rare diseases - IMPACT ON SOCIETY

We will now look further into the **subcriteria** associated with each criterion. We will first focus on the sub-criteria used to measure **societal** **HEALTH needs**.

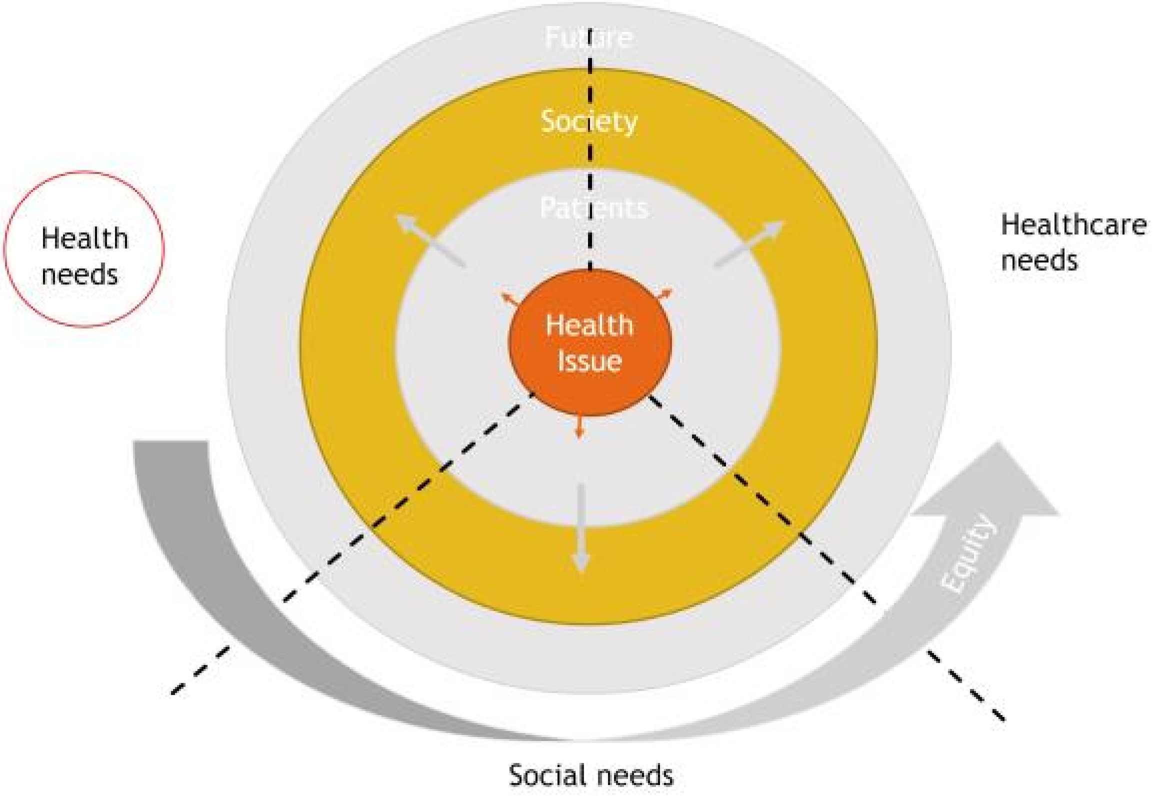

For the following set of questions, we kindly invite you to take a look at the sub-criteria, layed out in the flowchart below:

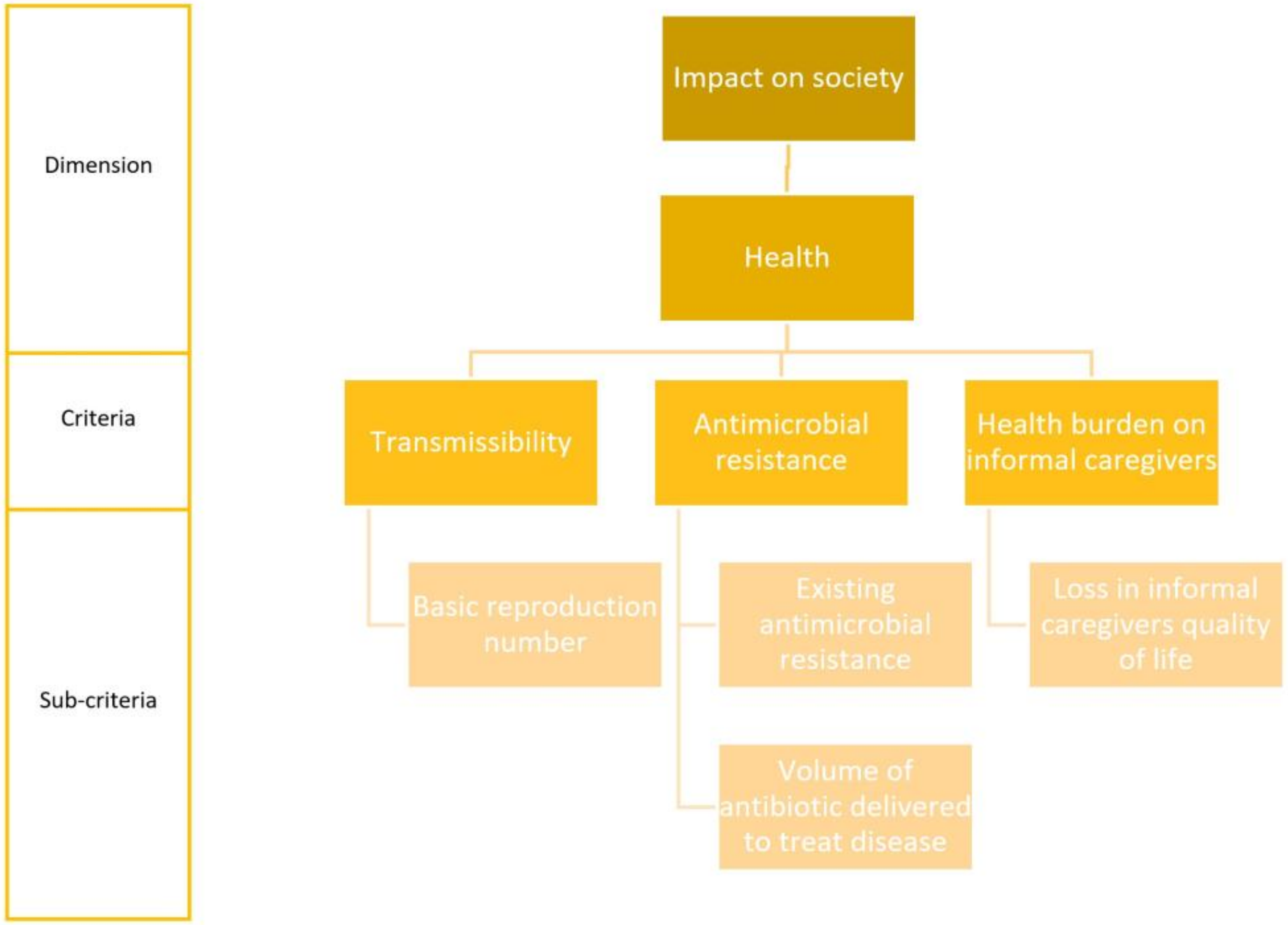

Transmissibility

The first criterion, within the dimension 'impact on society, generating health needs' is Transmissibility. The criterion 'Transmissibility' measures the extent to which a health issue can be transmitted from one organism to another.

Do you believe that one or more sub-criteria should be added, adapted or removed to assess the needs related to transmissibility in the context of rare diseases?

- ◯ Yes, the sub-criteria that should be added/adapted/removed and the reasons why are the following: ________
- ◯ No
- ◯ I do not know / I am not competent to answer this question

Do you have any additional concerns or suggestions related to this criterion or sub-criteria in relation to rare diseases?

- Yes
- No

In this table the sub-criterion of transmissibility is represented. Please indicate to what extent you agree with the proposed method being used to measure the sub-criterion.

	Strongly disagree	Disagree	Slightly disagree	Slightly agree	Agree	Strongly agree	I have insufficient knowledge
Basic reproduction number							
Data from Sciensano	○	○	○	○	○	○	○

Do you have any suggestions, alternatives or challenges regarding the method(s) used to measure the sub-criteria that you want to share? (If you propose an alternative method, please provide also a reference)

Antimicrobial resistance

The second criterion, within the dimension 'impact on society, generating health needs' is Antimicrobial resistance. The criterion 'Antimicrobial resistance' measures the extent to which antibiotics and other antimicrobial medicines used to treat a health issue are ineffective or infections related to a health issue become increasingly difficult or impossible to treat due to drug resistance.

Do you believe that one or more sub-criteria should be added, adapted or removed to assess the needs related to antimicrobial resistance in the context of rare diseases?

- Yes, the sub-criteria that should be added/adapted/removed and the reasons why are the following:
- No
- I do not know / I am not competent to answer this question

Do you have any additional concerns or suggestions related to this criterion or sub-criteria in relation to rare diseases?

- ○ Yes
- ○ No

In this table the sub-criteria of antimicrobial resistance are listed. Please indicate to what extent you agree with the proposed method(s) being used to measure the sub-criteria.

	Strongly disagree	Disagree	Slightly disagree	Slightly agree	Agree	Strongly agree	I have insufficient knowledge
**Existing antimicrobial resistance to treatments used**							
**Expert opinion**	○	○	○	○	○	○	○
**Volume of antibiotic delivered to treat disease (proxy)**							
**The RIZIV-INAMI Pharmanet database**	○	○	○	○	○	○	○

Do you have any suggestions, alternatives or challenges regarding the method(s) used to measure the sub-criteria that you want to share? (If you propose an alternative method, please provide also a reference)

Health burden on informal caregivers

The third criterion, within the dimension 'impact on society, generating health needs' is Health burden on caregivers. The criterion 'Health burden on caregivers' measures the extent to which the quality of life of informal caregivers is affected by their caregiving activities.

Do you believe that one or more sub-criteria should be added, adapted or removed to assess the needs related to health burden on informal caregivers in the context of rare diseases?

- ○ Yes, the sub-criteria that should be added/adapted/removed and the reasons why are the following: ________
- ○ No
- ○ I do not know / I am not competent to answer this question

Do you have any additional concerns or suggestions related to this criterion or sub-criteria in relation to rare diseases?

- ○ Yes ________
- ○ No

In this table the sub-criterion of health burden on informal caregivers is represented. Please indicate to what extent you agree with the proposed method being used to measure the sub-criterion.

	Strongly disagree	Disagree	Slightly disagree	Slightly agree	Agree	Strongly agree	I have insufficient knowledge
Loss in informal caregiver's quality of life							
**Literature review**	○	○	○	○	○	○	○

Do you have any suggestions, alternatives or challenges regarding the method(s) used to measure the sub-criteria that you want to share? (If you propose an alternative method, please provide also a reference)

For most sub-criteria, a literature review is performed alongside the other methods (e.g. survey). Do you agree with this? If not, please specify why.

- Yes
- No
- I do not know

In case something is unclear, or you would like additional information, please feel free to contact Zilke Claessens (zilke.claessens@kuleuven.be)

Part 2: Considerations for the application of the NEED framework and its criteria in the context of rare diseases - IMPACT ON SOCIETY

Secondly, the focus is on the sub-criteria used to measure **societal HEALTHCARE needs**:

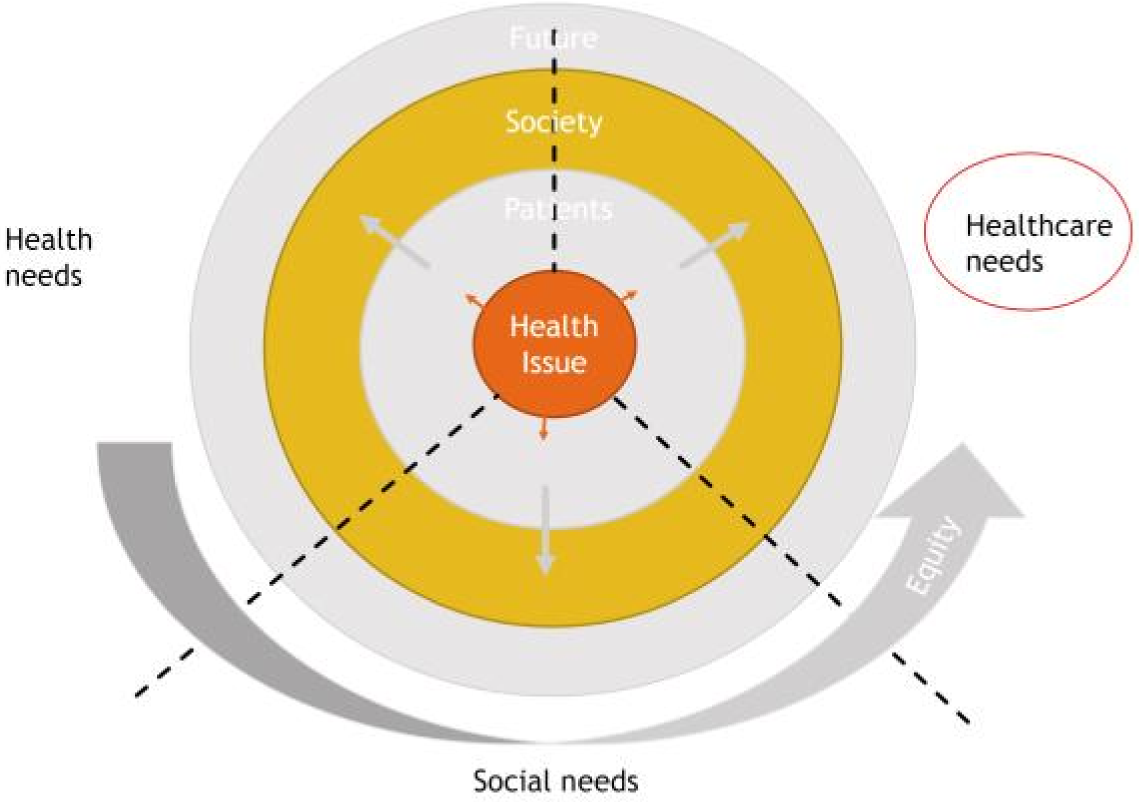

For the following set of questions, we kindly invite you to take a look at the sub-criteria, layed out in the flowchart below:

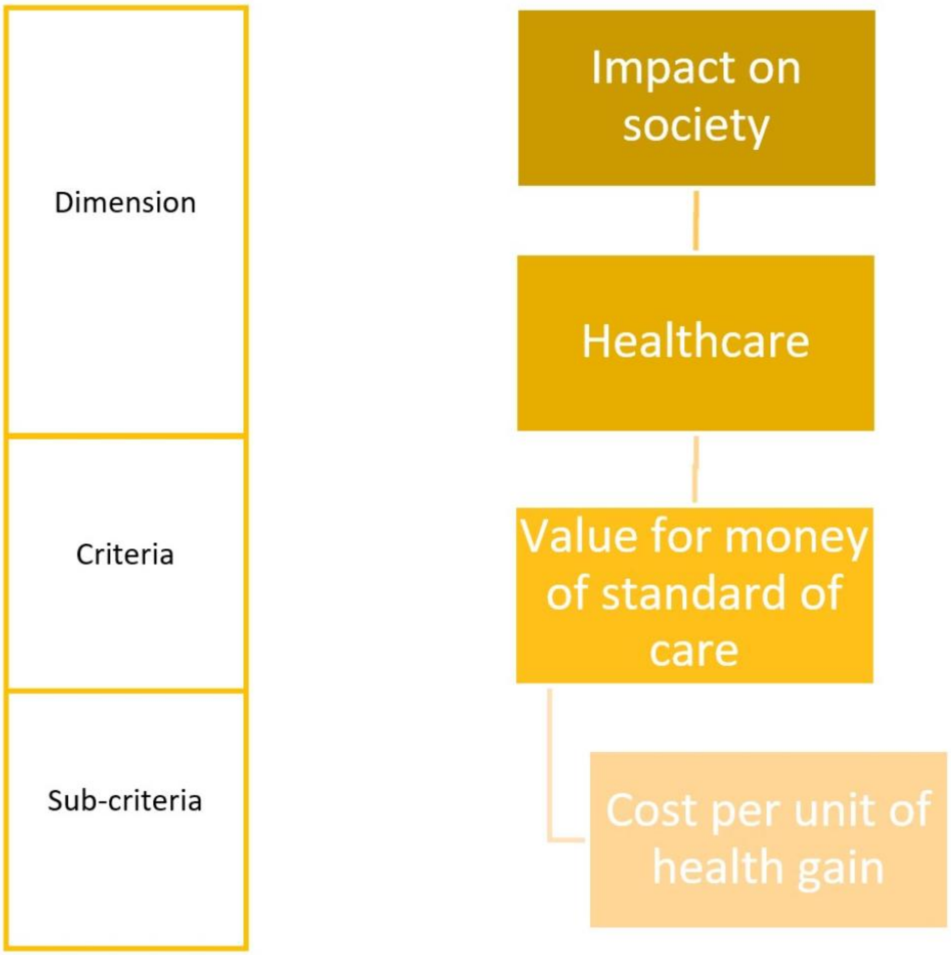

Value for money of standard of care

The only criterion, within the dimension 'impact on society, generating healthcare needs' is Value for money of standard of care. The criterion 'Value for money of standard of care' measures the health gain achieved for the level of healthcare spending associated with the standard of care for a health issue. It facilitates the identification of inefficiencies in the healthcare system.

Do you believe that one or more sub-criteria should be added, adapted or removed to assess the needs related to value for money of standard of care in the context of rare diseases?

- Yes, the sub-criteria that should be added/adapted/removed and the reasons why are the following: ______
- No
- I do not know / I am not competent to answer this question

Do you have any additional concerns or suggestions related to this criterion or sub-criteria in relation to rare diseases?

- ○ Yes [ ]
- ○ No

In this table the sub-criterion of value for money of standard of care is represented. Please indicate to what extent you agree with the proposed method being used to measure the sub-criterion.

	Strongly disagree	Disagree	Slightly disagree	Slightly agree	Agree	Strongly agree	I have insufficient knowledge
Healthcare costs per unit of health gain (disaggregated presentation of total healthcare expenditures and total health effects of standard of care)							
Different databases	○	○	○	○	○	○	○

Do you have any suggestions, alternatives or challenges regarding the method(s) used to measure the sub-criteria that you want to share? (If you propose an alternative method, please provide also a reference)

[ ]

In case something is unclear, or you would like additional information, please feel free to contact Zilke Claessens (zilke.claessens@kuleuven.be)

Part 2: Considerations for the application of the NEED framework and its criteria in the context of rare diseases - IMPACT ON SOCIETY

Thirdly, the focus is on the sub-criteria used to measure **SOCIAL needs of the society**:

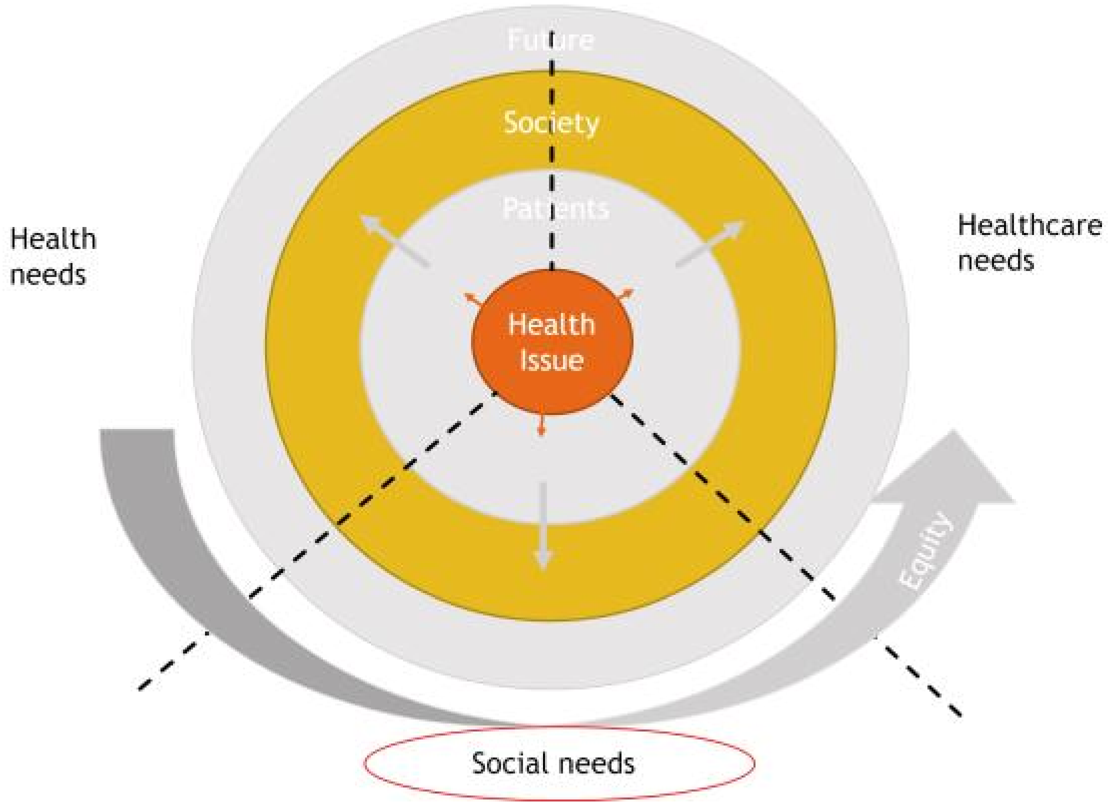

For the following set of questions, we kindly invite you to take a look at the sub-criteria, layed out in the flowchart below:

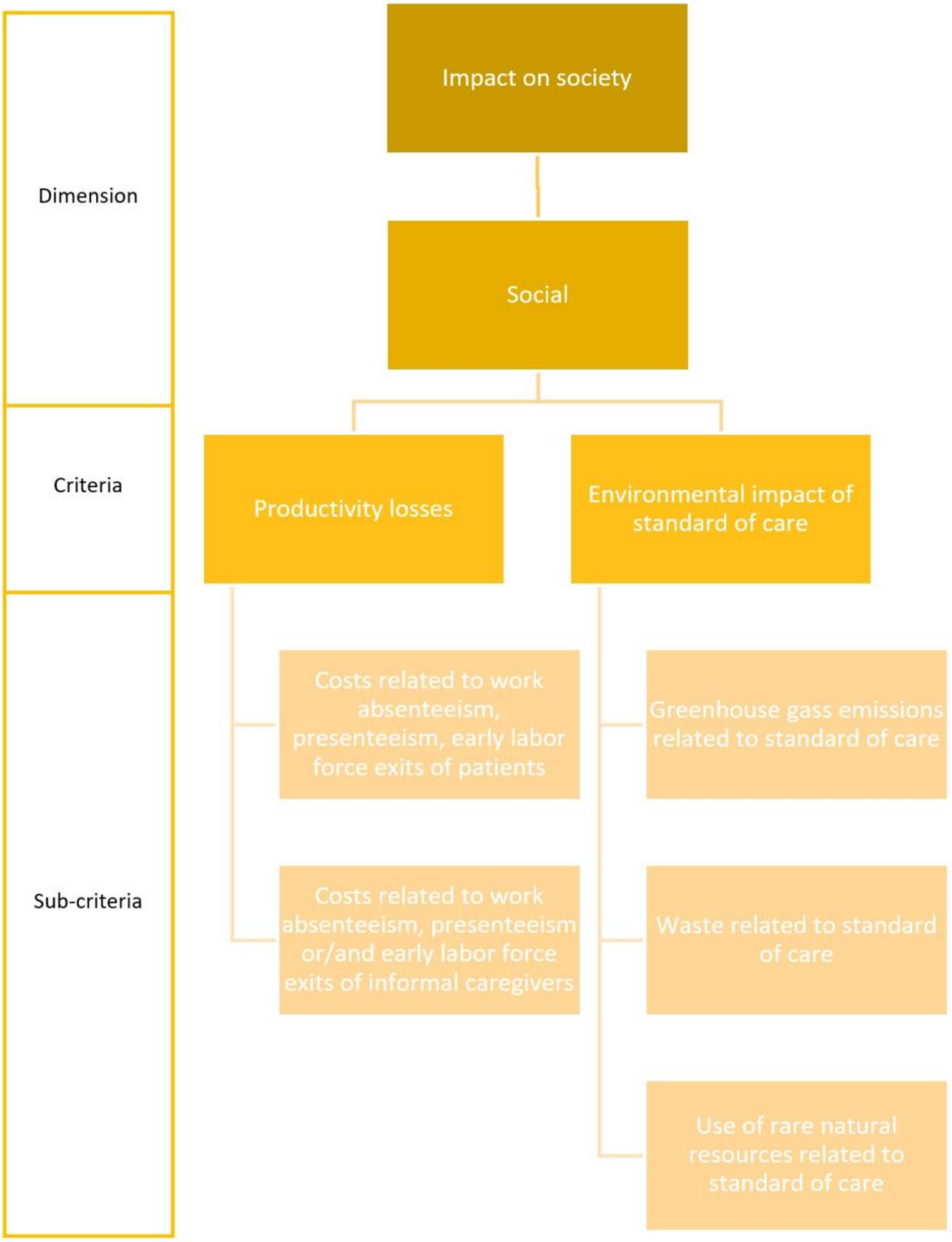

Productivity losses

The first criterion, within the dimension 'impact on society, generating social needs' is Productivity losses. The criterion 'Productivity losses' measures the costs related to

work absenteeism, presenteeism, early labor force exits of patients with the health issue and their caregivers.

Do you believe that one or more sub-criteria should be added, adapted or removed to assess the needs related to productivity losses in the context of rare diseases?

- ◯ Yes, the sub-criteria that should be added/adapted/removed and the reasons why are the following: ____
- ◯ No
- ◯ I do not know / I am not competent to answer this question

Do you have any additional concerns or suggestions related to this criterion or sub-criteria in relation to rare diseases?

- ◯ Yes ____
- ◯ No

In this table the sub-criteria of productivity losses are listed. Please indicate to what extent you agree with the proposed method(s) being used to measure the sub-criteria.

	Strongly disagree	Disagree	Slightly disagree	Slightly agree	Agree	Strongly agree	I have insufficient knowledge
**Costs related to work absenteeism, presenteeism, early labor force exits of patients**							
**Different databases**	◯	◯	◯	◯	◯	◯	◯
**Costs related to work absenteeism, presenteeism or/and early labor force exits of caregivers**							
**Literature review**	◯	◯	◯	◯	◯	◯	◯

Do you have any suggestions, alternatives or challenges regarding the method(s) used to measure the sub-criteria that you want to share? (If you propose an alternative method, please provide also a reference)

Environmental impact of standard of care

The second criterion, within the dimension 'impact on society, generating social needs' is Environmental impact of standard of care. The criterion 'Environmental impact of standard of care' measures the impact of the management of a health issue on the natural environment.

Do you believe that one or more sub-criteria should be added, adapted or removed to assess the needs related to environmental impact of standard of care in the context of rare diseases?

- ○ Yes, the sub-criteria that should be added/adapted/removed and the reasons why are the following: ______
- ○ No
- ○ I do not know / I am not competent to answer this question

Do you have any additional concerns or suggestions related to this criterion or sub-criteria in relation to rare diseases?

- ○ Yes ______
- ○ No

In this table the sub-criteria of environmental impact of standard of care are listed. Please indicate to what extent you agree with the proposed method(s) being used to measure the sub-criteria.

	Strongly disagree	Disagree	Slightly disagree	Slightly agree	Agree	Strongly agree	I have insufficient knowledge
**Greenhouse gas emissions related to the standard of care**							
**Literature review**	○	○	○	○	○	○	○
**Expert opinion**	○	○	○	○	○	○	○

	Strongly disagree	Disagree	Slightly disagree	Slightly agree	Agree	Strongly agree	I have insufficient knowledge
**Waste related to standard of care**							
Literature review	○	○	○	○	○	○	○
Expert opinion	○	○	○	○	○	○	○
**Use of rare natural resources related to standard of care**							
Literature review	○	○	○	○	○	○	○
Expert opinion	○	○	○	○	○	○	○

Do you have any suggestions, alternatives or challenges regarding the method(s) used to measure the sub-criteria that you want to share? (If you propose an alternative method, please provide also a reference)

For most sub-criteria, a literature review is performed alongside the other methods (e.g. survey). Do you agree with this? If not, please specify why.

- ○ Yes
- ○ No
- ○ I do not know

Do you have any **other suggestions** regarding certain **social needs** **criteria or sub-criteria** that should be **added, removed or adapted** to make the framework fit for the assessment of needs in **rare diseases**?

In case something is unclear, or you would like additional information, please feel free to contact Zilke Claessens (zilke.claessens@kuleuven.be)

Part 2: Considerations for the application of the NEED framework and its criteria in the context of rare diseases - FUTURE IMPACT

Now, we will zoom in on the **third dimension** and its criteria: **future impact**

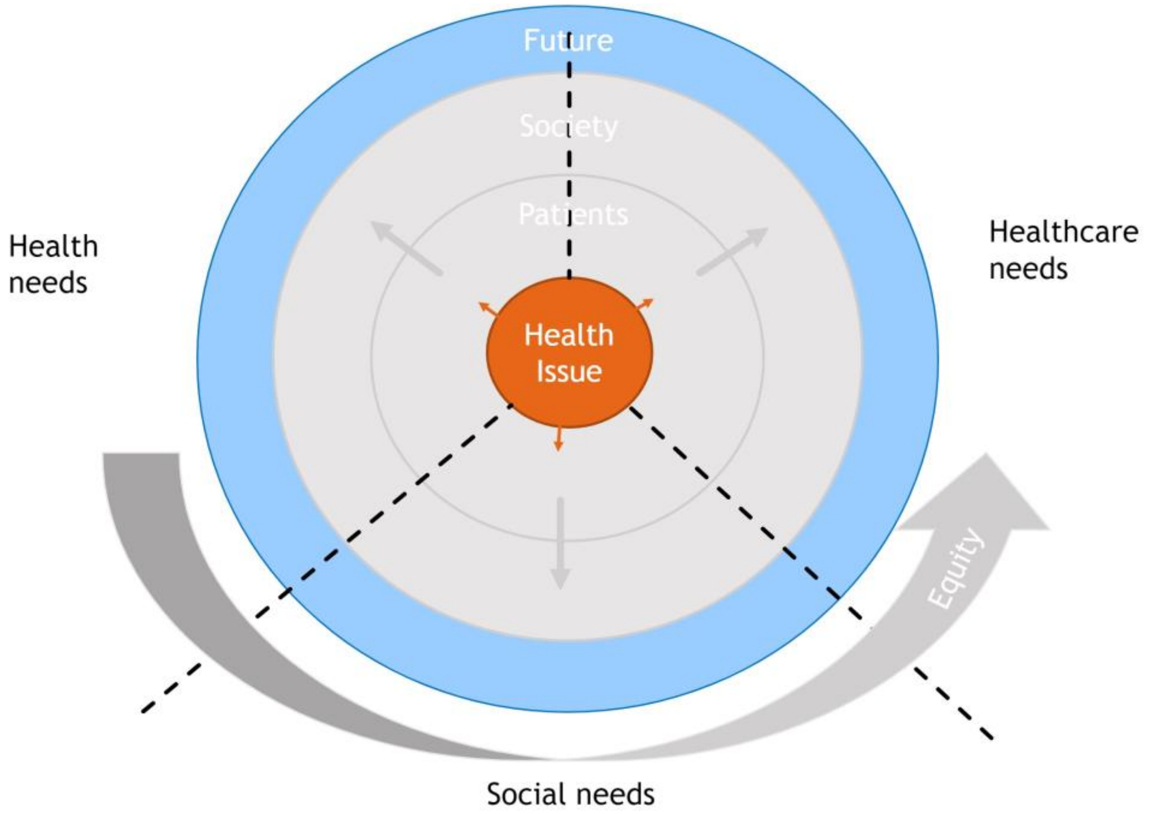

The criteria used to measure the **impact of a health issue on the future** are shown in the figure below:

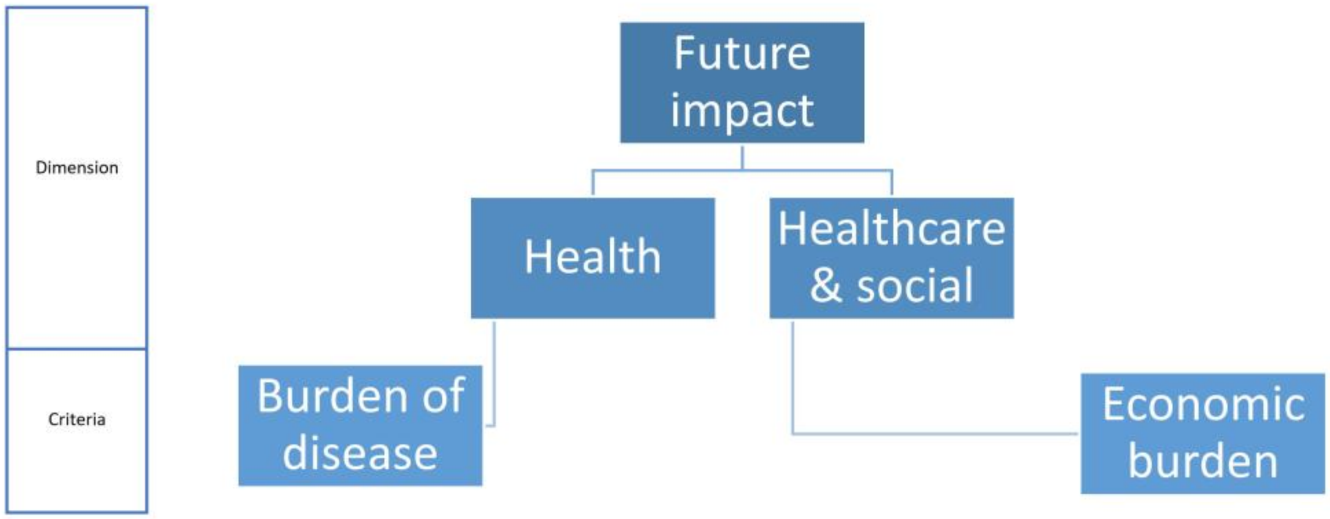

Do you have any comments related to the criteria of the dimension 'Future impact' in the context of rare diseases?

Do you believe that one or more criteria should be added, adapted or removed in the context of rare diseases?

- Yes, the criteria that should be added/adapted/removed and the reasons why are the following:
- No
- I do not know / I am not competent to answer this question

In case something is unclear, or you would like additional information, please feel free to contact Zilke Claessens (zilke.claessens@kuleuven.be)

Part 2: Considerations for the application of the NEED framework and its criteria in the context of rare diseases - FUTURE IMPACT

We will now look further into the **subcriteria** associated with each criterion. We will first focus on the sub-criteria used to measure **future HEALTH needs**.

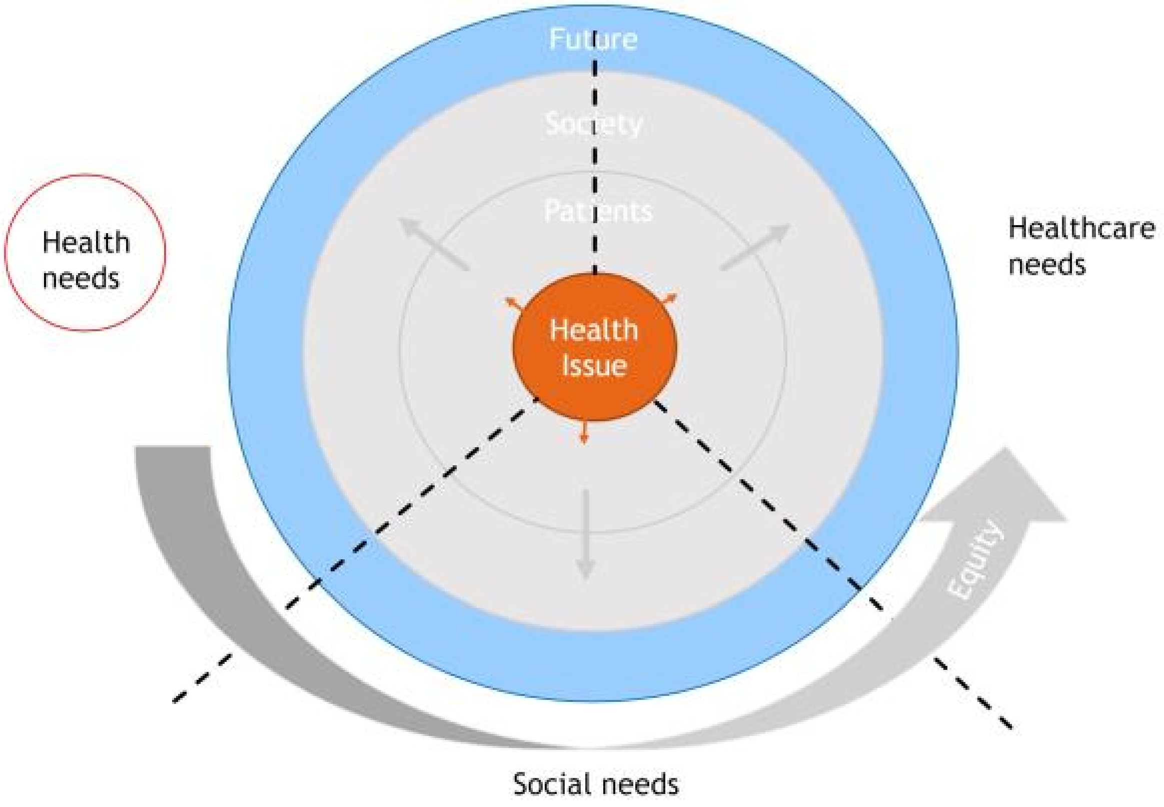

For the following set of questions, we kindly invite you to take a look at the sub-criteria, layed out in the flowchart below:

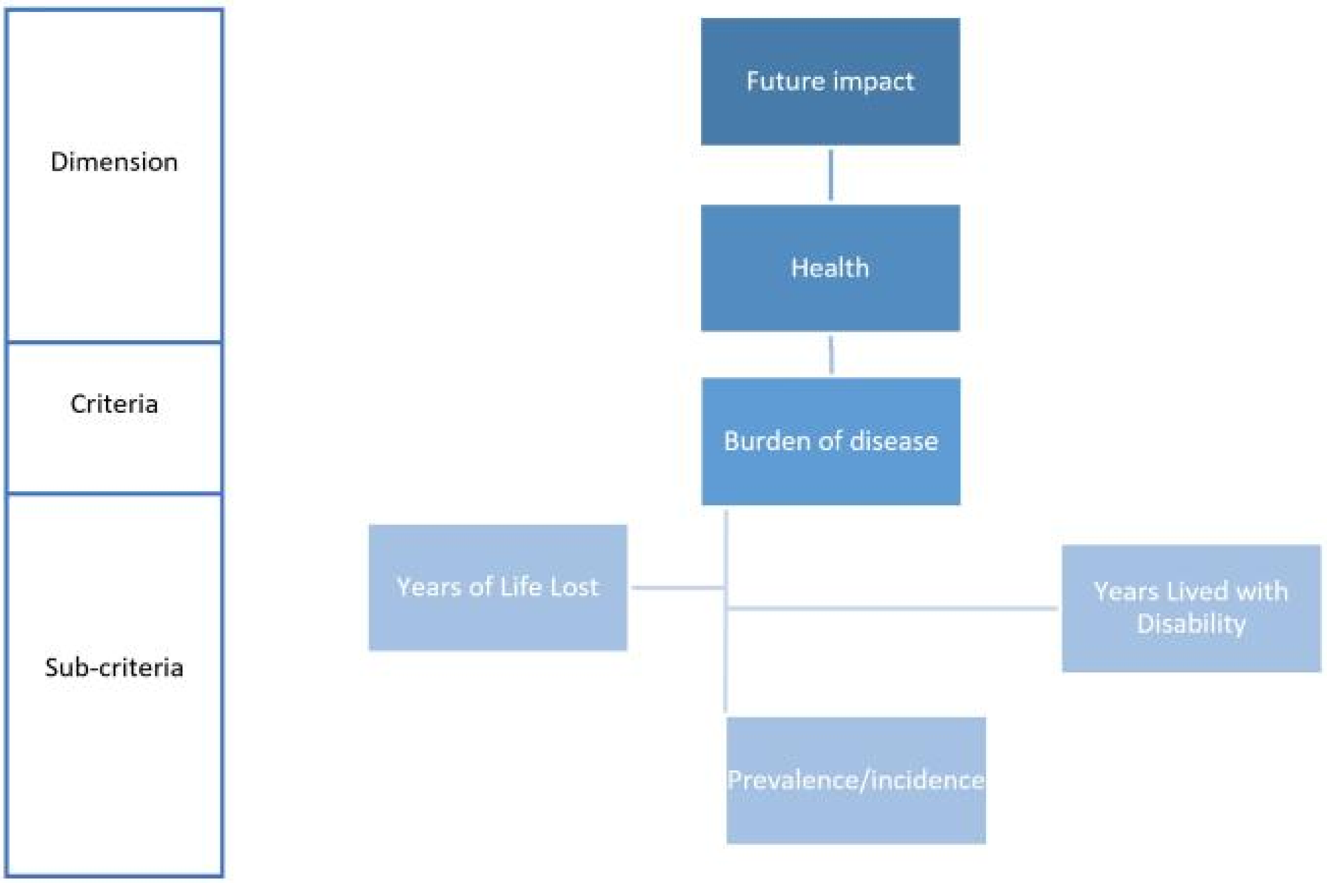

Burden of disease

The only criterion, within the dimension 'Future impact, generating health needs' is Burden of disease. The criterion 'Burden of disease' measures the future impact of a health issue on population's physical and psychological health.

Do you believe that one or more sub-criteria should be added, adapted or removed to assess the needs related to burden of disease in the context of rare diseases?

- ◯ Yes, the sub-criteria that should be added/adapted/removed and the reasons why are the following: ________
- ◯ No
- ◯ I do not know / I am not competent to answer this question

Do you have any additional concerns or suggestions related to this criterion or sub-criteria in relation to rare diseases?

- ◯ Yes ________

○ No

In this table the sub-criteria of burden of disease are listed. Please indicate to what extent you agree with the proposed method(s) being used to measure the sub-criteria.

	Strongly disagree	Disagree	Slightly disagree	Slightly agree	Agree	Strongly agree	I have insufficient knowledge
Prevalence/Incidence							
Belgian Burden of Disease Study (BeBoD)	○	○	○	○	○	○	○
Years of Life Lost (Mortality)							
Belgian Burden of Disease Study (BeBoD)	○	○	○	○	○	○	○
Years Lived with Disability (Morbidity)							
Belgian Burden of Disease Study (BeBoD)	○	○	○	○	○	○	○

Do you have any suggestions, alternatives or challenges regarding the method(s) used to measure the sub-criteria that you want to share? (If you propose an alternative method, please provide also a reference)

For most sub-criteria, a literature review is performed alongside the other methods (e.g. survey). Do you agree with this? If not, please specify why.

○ Yes

○ No

○ I do not know

In case something is unclear, or you would like additional information, please feel free to contact Zilke Claessens (zilke.claessens@kuleuven.be)

Part 2: Considerations for the application of the NEED framework and its criteria in the context of rare diseases - FUTURE IMPACT

Secondly, the focus is on the sub-criteria used to measure **future** **HEALTHCARE & SOCIAL needs**.

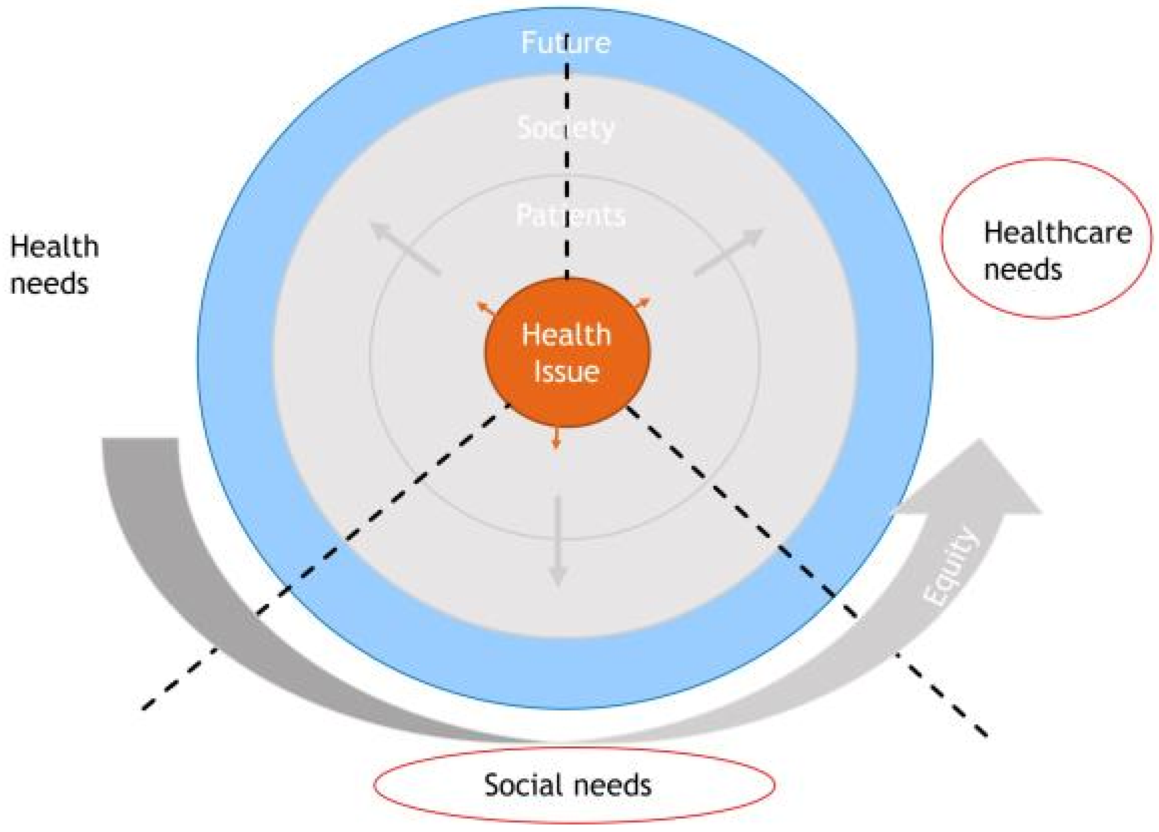

For the following set of questions, we kindly invite you to take a look at the sub-criteria, layed out in the flowchart below:

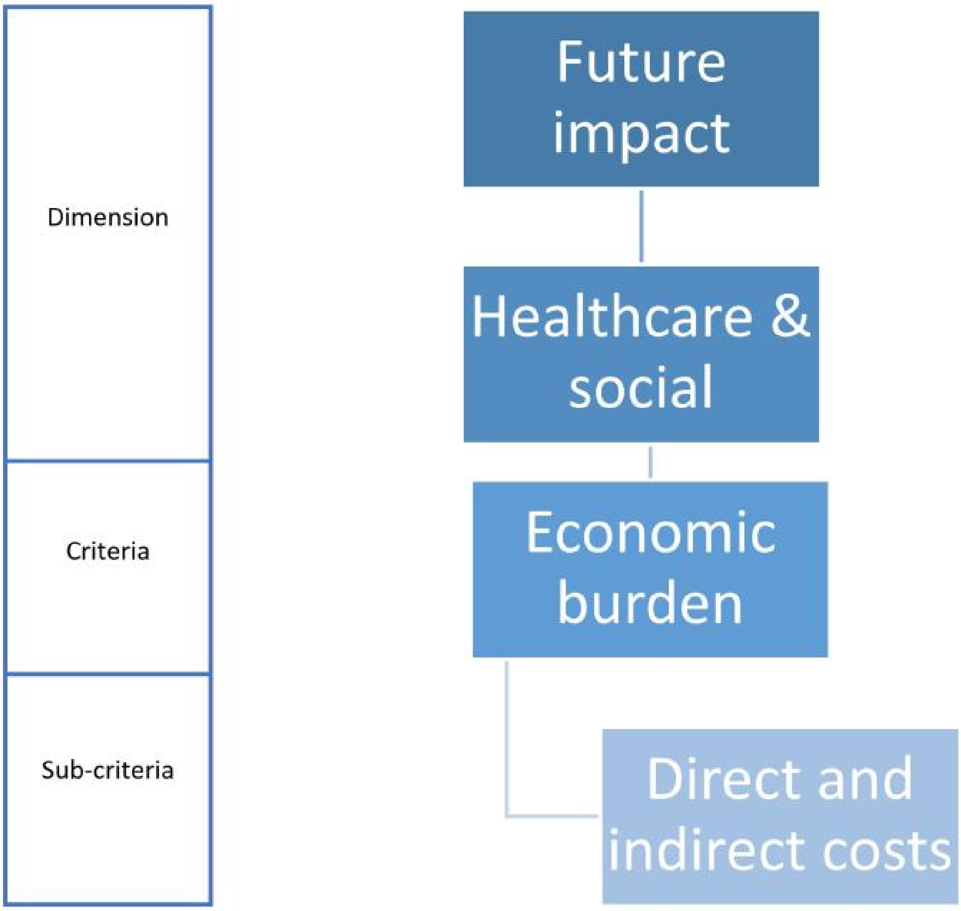

Direct cost
Indirect costs

Economic burden

The only criterion, within the dimension 'Future impact, generating healthcare & social needs' is Economic burden. The criterion 'Economic burden' measures the future impact of a health issue on healthcare expenditures and on productivity, in monetary terms.

Do you believe that one or more sub-criteria should be added, adapted or removed to assess the needs related to economic burden in the context of rare diseases?

- Yes, the sub-criteria that should be added/adapted/removed and the reasons why are the following: ___
- No
- I do not know / I am not competent to answer this question

Do you have any additional concerns or suggestions related to this criterion or sub-criteria in relation to rare diseases?

- Yes
- No

In this table the sub-criteria of economic burden are listed. Please indicate to what extent you agree with the proposed method(s) being used to measure the sub-criteria.

	Strongly disagree	Disagree	Slightly disagree	Slightly agree	Agree	Strongly agree	I have insufficient knowledge
**Direct costs**							
Belgian Burden of Disease Study (BeBoD)	○	○	○	○	○	○	○
**Indirect costs**							
Belgian Burden of Disease Study (BeBoD)	○	○	○	○	○	○	○

Do you have any suggestions, alternatives or challenges regarding the method(s) used to measure the sub-criteria that you want to share? (If you propose an alternative method, please provide also a reference)

Do you have any **other suggestions** regarding certain **future needs criteria or sub-criteria** that should be **added, removed or adapted** to make the framework fit for the assessment of needs in **rare diseases**?

In case something is unclear, or you would like additional information, please feel free to contact Zilke Claessens (zilke.claessens@kuleuven.be)

Part 2: Considerations for the application of the NEED framework and its criteria in the context of rare diseases - TRANSVERSAL

Across the 3 dimensions discussed before, there is also a **transversal criterion**: **equity**.

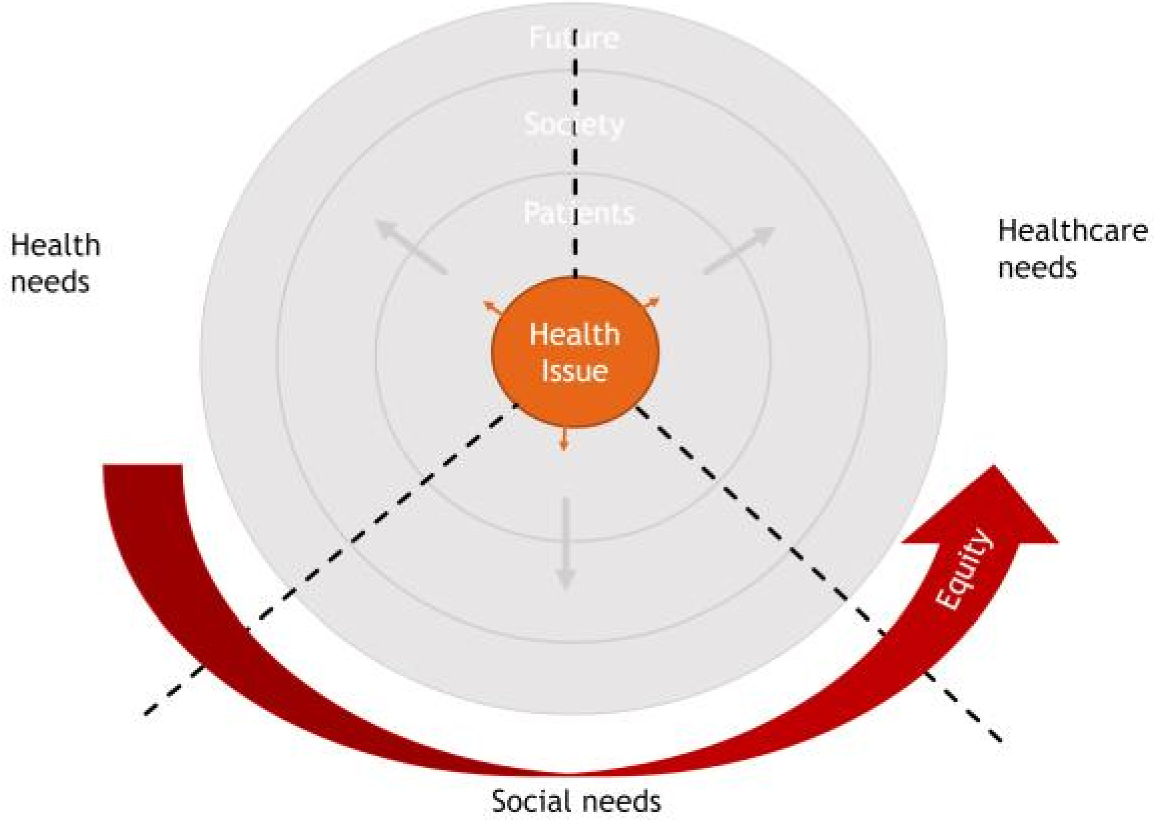

Transversal impact of a health issue on equity: A given health issue has an impact on equity from a patient and a society's perspective. Equity implies the absence of unfair and avoidable or remediable differences in health and healthcare among population groups defined socially, economically, demographically or geographically or by other dimensions of inequality.

When relevant, evidence for patient, societal or future needs criteria and sub-criteria will be presented by population subgroup.

In this table possible methods for measuring equity are listed. Please indicate to what extent you agree with the proposed method(s).

	Strongly disagree	Disagree	Slightly disagree	Slightly agree	Agree	Strongly agree	I have insufficient knowledge
**Equity**							

	Strongly disagree	Disagree	Slightly disagree	Slightly agree	Agree	Strongly agree	I have insufficient knowledge
**Survey question**	○	○	○	○	○	○	○
**Literature review**	○	○	○	○	○	○	○
**Different databases**	○	○	○	○	○	○	○

Do you have any comments related to this transveral criterion or suggestions for other transversal criteria that need to be added to the framework in the context of rare diseases?

In case something is unclear, or you would like additional information, please feel free to contact Zilke Claessens (zilke.claessens@kuleuven.be)

Powered by Qualtrics

Default Question Block

Dear participant,

Thank you for taking part in the **second stakeholder workshop** organised by **the Belgian Healthcare Knowledge Centre (KCE)** and **KU Leuven** regarding the NEED (Needs Examination, Evaluation and Dissemination) project: "**Applicability of the NEED approach to assess patient and societal needs in patients suffering from rare diseases.**"

Your perspective and insights will help us refine the NEED assessment framework and adapt it to the context of rare diseases, in order to better identify and prioritize health conditions with likely high unmet needs. In order to do so, we need to **capture the diverse challenges and considerations related to rare disease** together with their impact on patients and society. Ultimately, identifying the highest unmet needs in the areas of prioritization may lead to better decision-making for these conditions, e.g., better research orientation, healthcare services organisation, and investment in research and development of treatment and technologies.

This survey aims to inform the **second stakeholder workshop** which will be held on **September 7th**.

The aim of this second workshop is **to understand your views on the 5-step implementation model for the evaluation of unmet needs proposed by the KCE** in the context of rare diseases (such as the identification of priority topics and the prioritization of these topics). The aim is also to **balance rare diseases against more common diseases** in the NEED approach.

The topic of 'unmet patient and societal needs' has been chosen as a priority area for the Belgian Presidency of the EU in 2024. Belgium aims to formulate Council conclusions by the end of its term. The findings of this research (including this survey and workshop) are intended to inform these council conclusions.

! Please note that the implementation model has recently been updated to include the evaluation of societal needs (7- to 5-step model) and that this survey contains the last available version.

How will my data be handled and used?

The data collected in this survey will be analysed and used to inform subsequent multistakeholder workshops. We present the aggregated data on the workshops in an anonymous way. However, we may contact you personally following the survey for further information.

This survey will take 10-15 minutes to complete.

Informed consent

To participate in this survey, you must provide your informed consent. Therefore, it is important that you understand and agree with the statements below.

- I have had the opportunity to read the information sheet concerning the study which aims to determine the applicability of the NEED framework, i.e. the criteria and methods, to assess patient and societal needs in patients suffering from rare diseases;
- I have had the opportunity to ask questions or discuss any concerns regarding the information of this study;
- I was given sufficient time to decide whether I was willing to participate in this study;
- I am aware that participation in this study is completely voluntary;
- I am aware that this questionnaire has been prepared in collaboration with the Belgian Healthcare Knowledge centre (KCE), as stated on the information sheet;
- I am aware that I can withdraw from this study at any time;
- I understand that the data resulting from this research will be kept for a maximum of 25 years after the end of the research:
- I understand that my data may be used for future research;
- I am aware that the results of this research will be published in scientific journals and disseminated through presentations at conferences or meetings. I

understand that these results are based on the analysis of anonymous data and cannot identify me in any way.

- I understand that my data will be processed in accordance with the European General Data Protection Regulation (GDPR).

Do you agree with the above statements?

- I agree with all the statements and confirm my participation
- I do not agree with all the statements and do not confirm my participation

Block 3

Background information

1. What is the NEED asssessment framework?

The KCE NEED ('Needs Examination, Evaluation and Dissemination') project developed a **conceptual approach called the NEED framework** (conceptual approach) to collect evidence for particular **health issues** or conditions on unmet health-related needs. Health-related needs can include "**health needs**, "**healthcare needs**", and "**other needs**" ("non-health(care) needs")(figure below). The evidence will be collected from three perspectives: **patients**, **society**, and **future generations**. The data will be summarised in a database accessible to all relevant stakeholders.

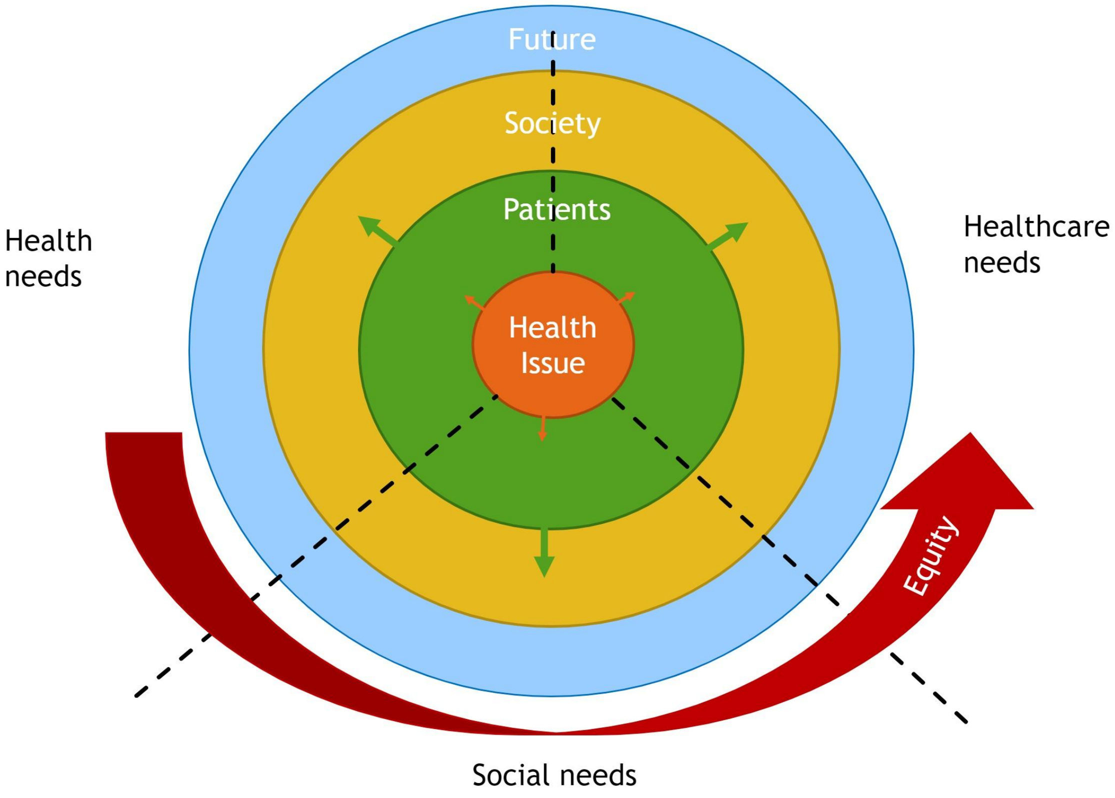

In the NEED framework, explicit criteria are used to collect evidence on the unmet needs related to a given health issue. These criteria are factors responsible for the burden of the health issue on patients as well as on the broader society, both in the present and in the future.

2. The NEED 5-step implementation model

Because it will be impossible to collect evidence for all health conditions at once, it will be necessary to identify and prioritise specific health conditions. Therefore, for the initial phase of the NEED project will involve a selection of topics based on a database search (step 1) and collection of proposals (step 2), followed by a prioritization process (step 3).

This 5-step implementation model is developed to (i) identify relevant topics (that is, diseases or health conditions that potentially have high unmet needs), (ii) select priority health conditions, (iii) apply the NEED assessment framework to identify unmet needs within each health condition, and (iv) disseminate the results.

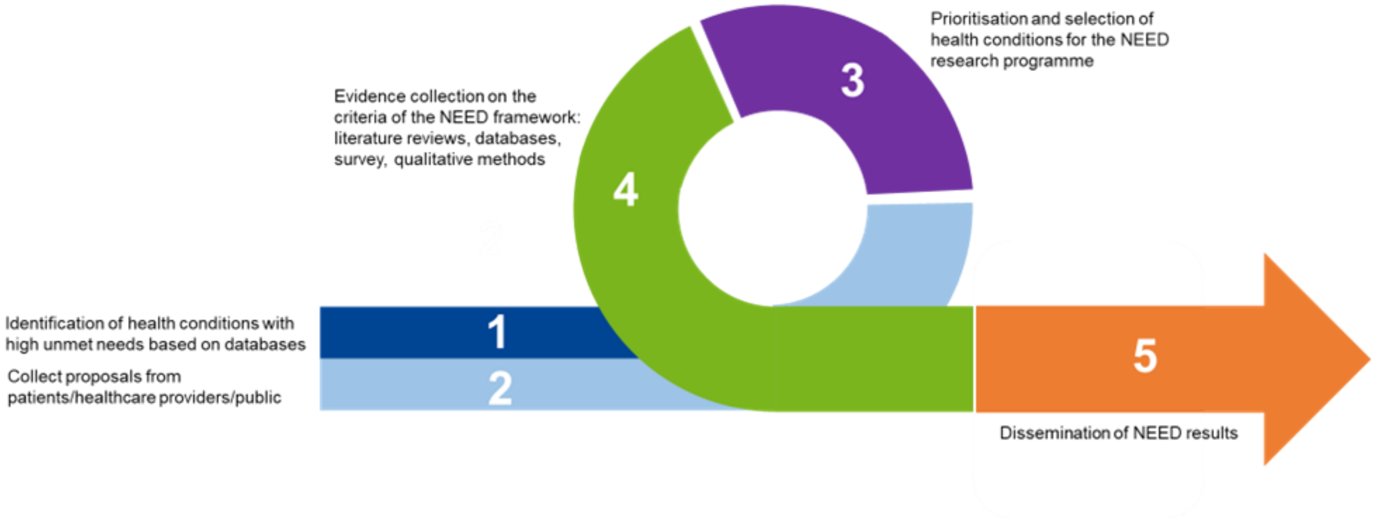

3. What is asked of me?

This survey consists of the following 2 parts:

- PART 1: **background questions** about yourself such as your name and organisation
- PART 2: questions regarding the 5-step **NEED implementation model**

4. What should I do if during the survey something is unclear, or if I would like additional information?

Please contact Zilke Claessens (zilke.claessens@kuleuven.be) if you are experiencing difficulties in completing the survey or would like any information regarding this survey.

Your answers are **saved automatically** by clicking on the blue arrow at the bottom of each page and **submitted automatically** when closing your browser.

Background questions

PART 1: Background questions

Name (optional):

Organisation (optional):

Which stakeholder category do you belong to?

- ☐ Regulator
- ☐ Policy-maker
- ☐ HTA body / payer
- ☐ Pharmaceutical industry / trade organisation
- ☐ Research organisation
- ☐ Healthcare professional
- ☐ Patient organisation
- ☐ Other:

Block 2

PART 2: Overall applicability of the NEED 5-step implementation model to the context of rare diseases

Steps in the NEED 5-step implementation model

Please consider the figure below, which represents the process for how the KCE would like to organise the assessment of unmet needs in rare diseases. This implementation model contains 5 steps to identify patient, societal, and future needs and is the updated version of the previous 7-step model (published and explained in KCE report 348 on page 170). The different steps of the 5-step implementation model are visualised in the figure below:

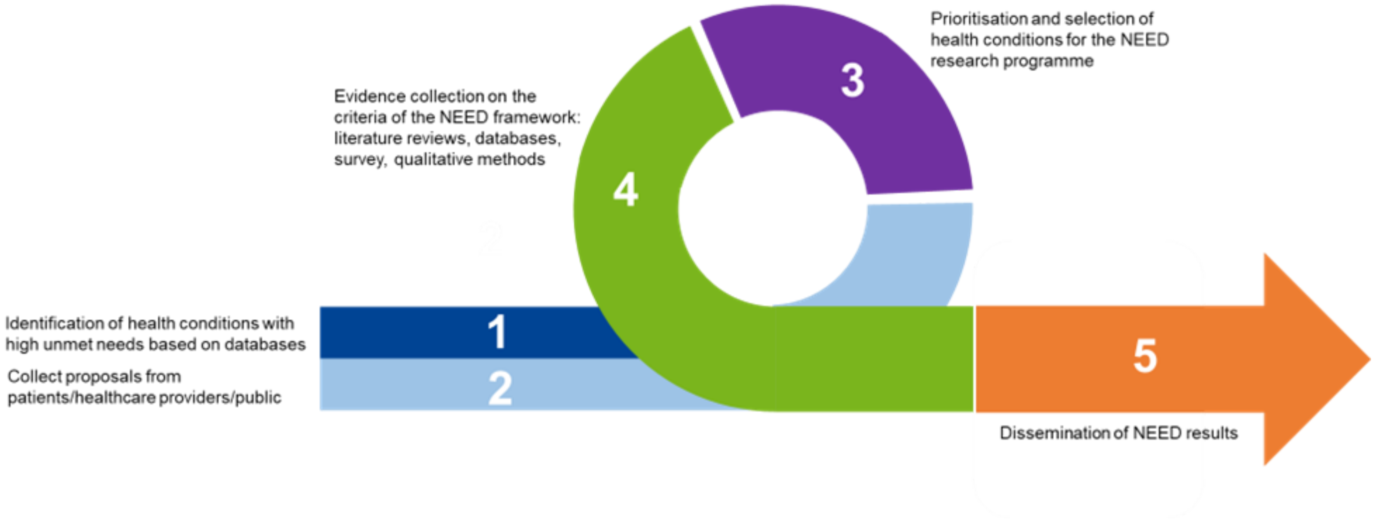

- **Step 1** involves the identification of health conditions with potential high unmet needs using existing databases.

- **Step 2** involves the call for proposals that will invite patients or patient organisations, healthcare providers, and the general public to highlight health conditions with potential high unmet needs.

- **Step 3** involves the prioritisation and selection of health conditions obtained from steps 1 and 2 for the NEED research program.

- **Step 4** involves, for each selected health condition, the collection of evidence on the unmet needs criteria following the methods in the NEED assessment framework. Evidence will be collected using literature reviews, database analysis, surveys, and qualitative methods.

- **Step 5** involves the dissemination of the results on the NEED website and to the relevant stakeholders.

First, we will ask you about your general remarks regarding the model before to go more in detail over the different steps.

Do you have any general remarks regarding this 5-step implementation model and its applicability to rare diseases?

◯ Yes, namely:

◯ No

Do you think certain steps of this proposed model should be adapted or new ones added to make it applicable to the context of rare diseases?

◯ Yes, namely:

◯ No

◯ I don't know

PART 2: Points of attention regarding the steps in the NEED 5-step implementation model

Step 1: Identification of priority topics (i.e. health issues or health conditions)

According to the 5-step implementation model, **Step 1** involves the identification of health conditions with potential high unmet needs using existing databases. In this step, **data from large databases** like the Belgian Burden of Disease database and the Global Burden of Disease database can be used. (KCE Report 348)

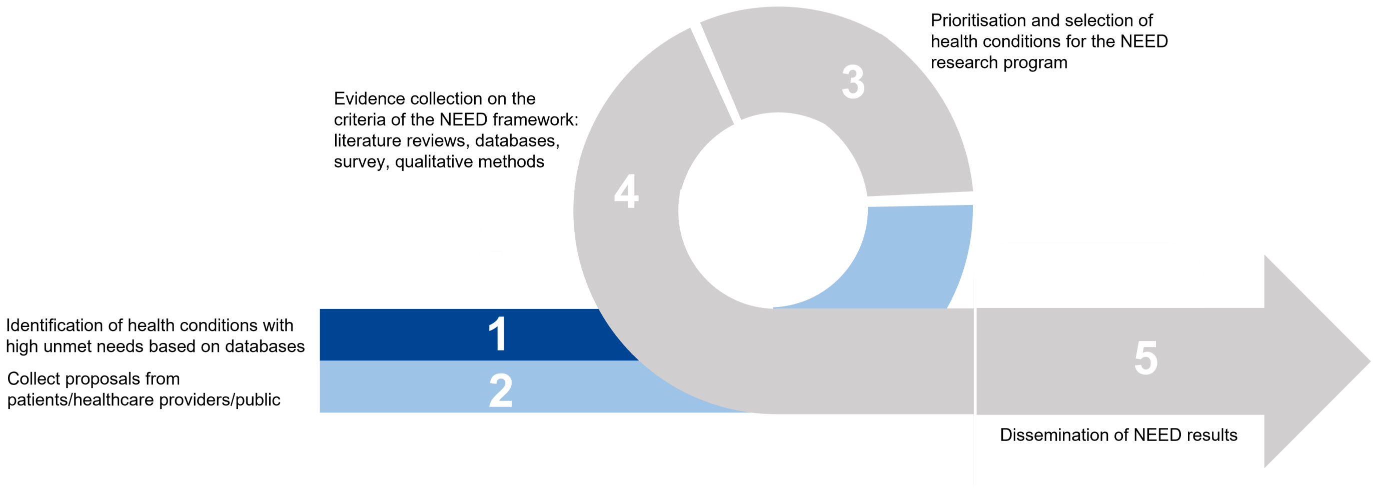

In general, how do you think priority topics with potential high unmet needs should be identified in rare diseases?

PART 2: Points of attention regarding the steps in the NEED 5-step implementation model

Step 1: Identificitation of priority topics (i.e. health issues/conditions)

According to the 5-step implementation model, **Step 1** involves the identification of health conditions for which in-depth data collection should be performed to identify the specific patients and societal needs. In this step, **data from large databases** like the Belgien Burden of Disease database and the Global Burden of Disease database can be used. (KCE Report 348)

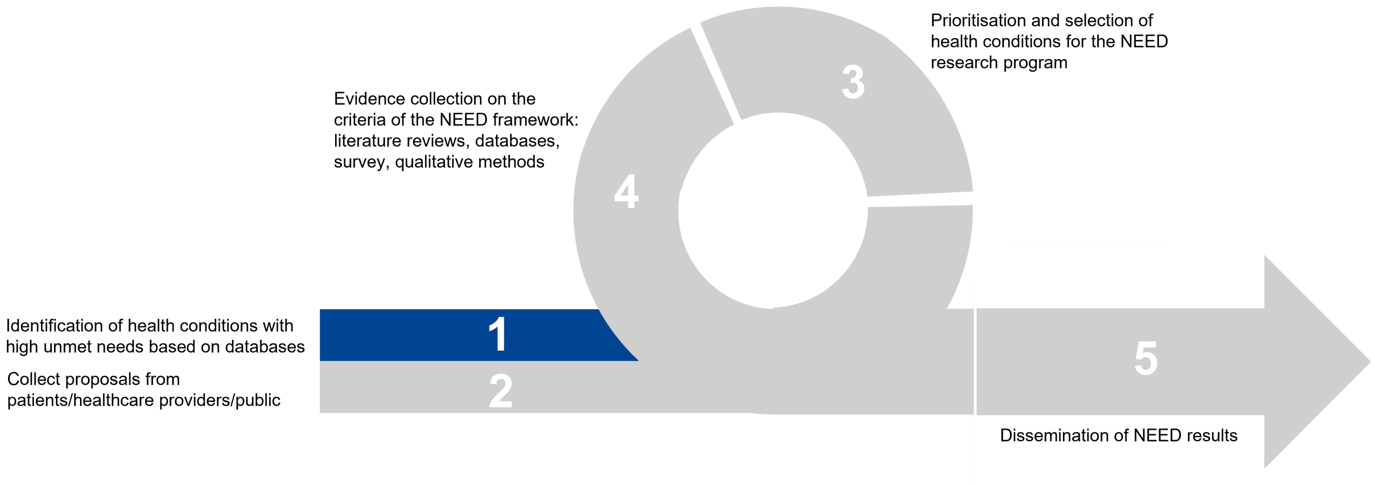

Do you believe that a combination of the proposed databases (Belgian Burden of Disease database, the database from the unmet medical needs program and the special solidarity fund of the NIHDI, international horizon scanning initiative, and the Global Burden of Disease database) is a good data source to help identify priority topics for the NEED assessment in the context of rare diseases, besides the open call for proposals (step 2)?

- Yes
- No; please provide the reason:
- I don't know

What type of data/metrics are fit for the purpose of identifying/prioritising rare diseases/conditions with potential high unmet health-related needs?

Do you believe that other rare disease-specific databases or sources should be used for the identification of priority topics?

- ○ Yes; which existing databases or sources:
- ○ No
- ○ I don't know

Do you have any recommendations regarding the use of databases for the identification of priority topics in the context of rare diseases?

PART 2: Points of attention regarding the steps in the NEED 5-step implementation model

Step 2: Call for topic proposals

In step 2, patients, patient organisations, healthcare professionals, and the general public can submit topic proposals (i.e. suggest a rare disease or health condition).

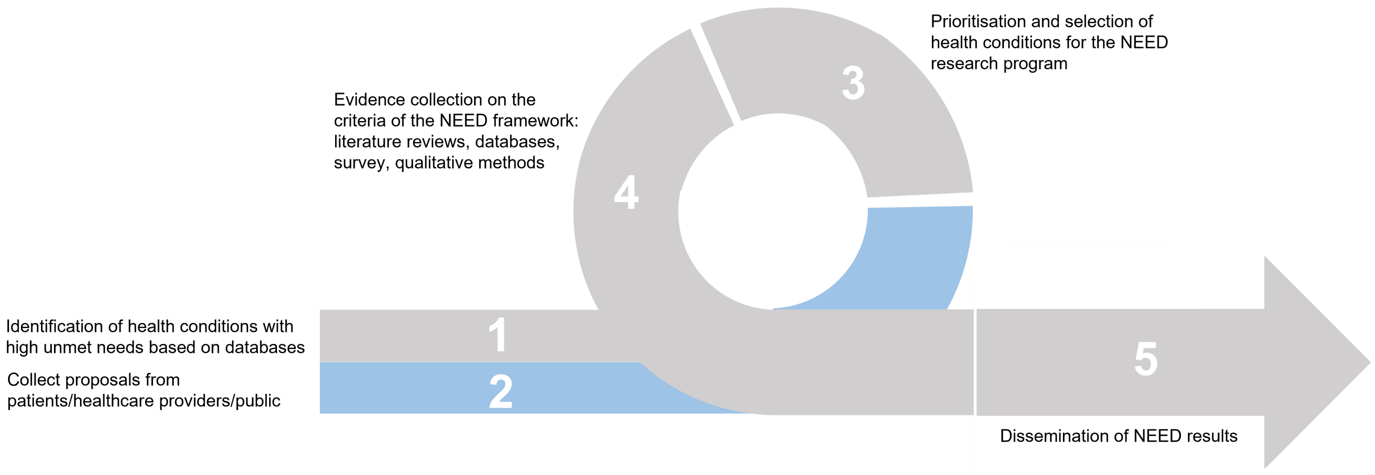

Do you believe that an open call for topics targeted towards patients, healthcare professionals and the general public is a relevant methodology to identify priority

topics with potential high unmet needs, in the context of rare diseases?

- Yes, please indicate if there are any points of attention:
- No, because:
- I don't know

What is the minimal information that should be requested in the call for rare disease topic proposals to enable the prioritisation of topics with potentially high unmet needs (step 3)?

Should the information requested in the call for proposals be different for rare and common diseases?

- Yes, explain why and how the information must differ in your opinion:
- No, because:
- I don't know

PART 2: Points of attention regarding the steps in the NEED 5-step implementation model

Step 3: Prioritization of topics with potentially high unmet needs

In step 3, priority topics will be selected, based on a set of pre-defined prioritisation criteria and an independent review of proposals (KCE Report 348).

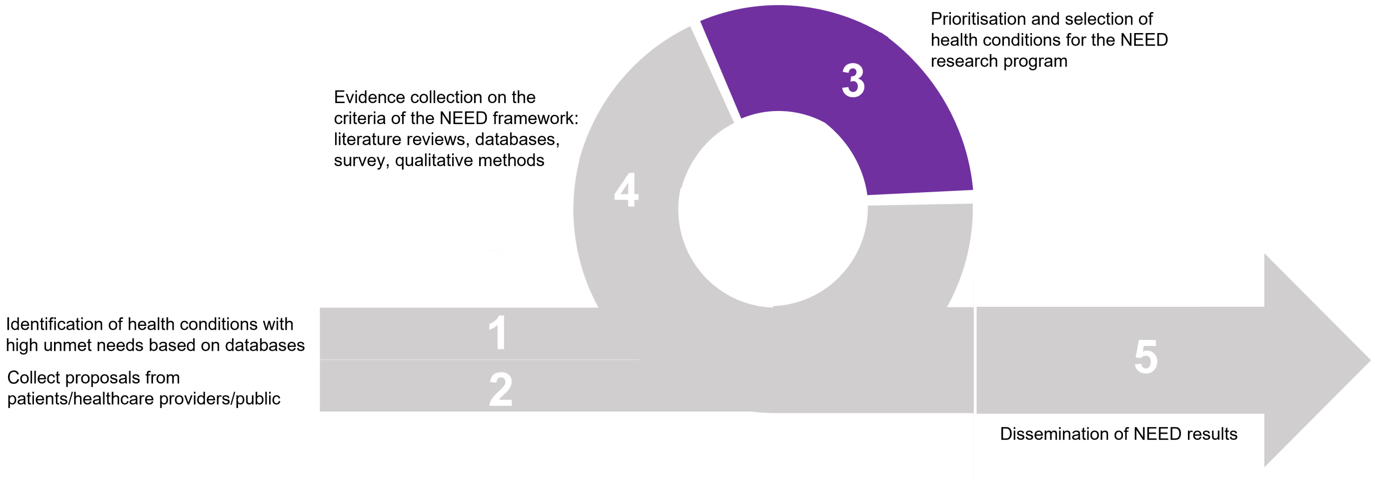

In your opinion, should priority topics for rare diseases be identified by means of the same procedures as for common diseases (i.e. based on explicit priority criteria) or should an alternative approach be taken?

What would be relevant prioritisation criteria for the selection of topics for unmet needs research amongst rare conditions?

Do you have other suggestions/remarks for the prioritisation of topics with high potential unmet needs in rare diseases?

PART 2: Points of attention regarding the steps in the NEED 5-step implementation model

Step 4: Evidence collection of the unmet needs within the selected health conditions, by using literature reviews, databases, surveys, and qualitative methods.

After the identification of topics (steps 1 and 2) and the prioritisation of topics (step 3), the KCE proposes the **4th step**, which consists of the **actual evidence collection on the health-related unmet needs** for each selected health condition. The collection of unmet needs will follow the pre-defined criteria of the NEED framework.

The evidence collection performed in step 4 includes different methods that are criteria-specific (discussed in workshop 1):

- **Literature reviews**: to (1) collect information to adapt the generic patient NEED survey and interview guide to the selected health condition and then (2) gather existing evidence on the unmet needs criteria.
- **Analysis of databases**
- **Implementation of adapted patient NEED survey** (developed by the KCE)
- **Implementation of a qualitative study:** among patients and experts, such as individual interviews and expert opinions

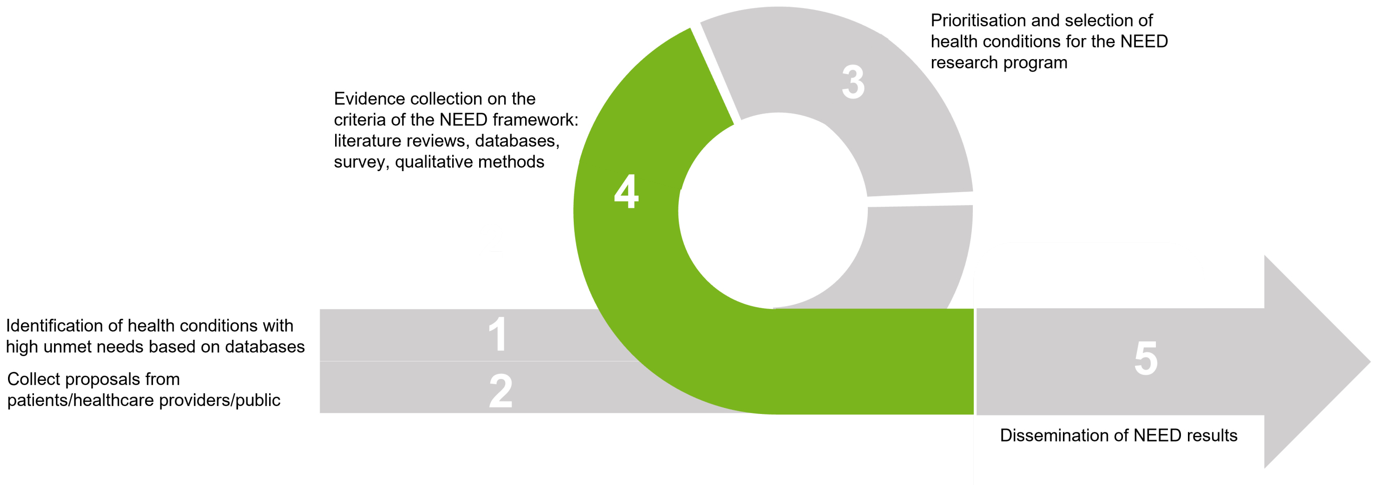

Do you have any recommendations or alternative methods for the NEED survey in case the number of patients living with the rare disease is very low?

- ◯ Yes, I propose the following: [ ]
- ◯ No
- ◯ I don't know

Do you have any ethical concerns (e.g. anonymisation) relating to the conduct of a survey among patients in the context of rare diseases?

- ◯ Yes, I propose the following: [ ]
- ◯ No
- ◯ I don't know

Do you have any other suggestions or remarks related to step 4 of the implementation model?

PART 2: Points of attention regarding the steps in the NEED 5-step implementation model

Step 5: Dissemination of the NEED results

The results of the NEED approach might be useful for diverse types of stakeholders, including but not limited to healthcare policymakers, RIZIV/INAMI, sickness funds, patient organisations, healthcare providers, the healthcare industry, research funding agencies, several regional organisations.

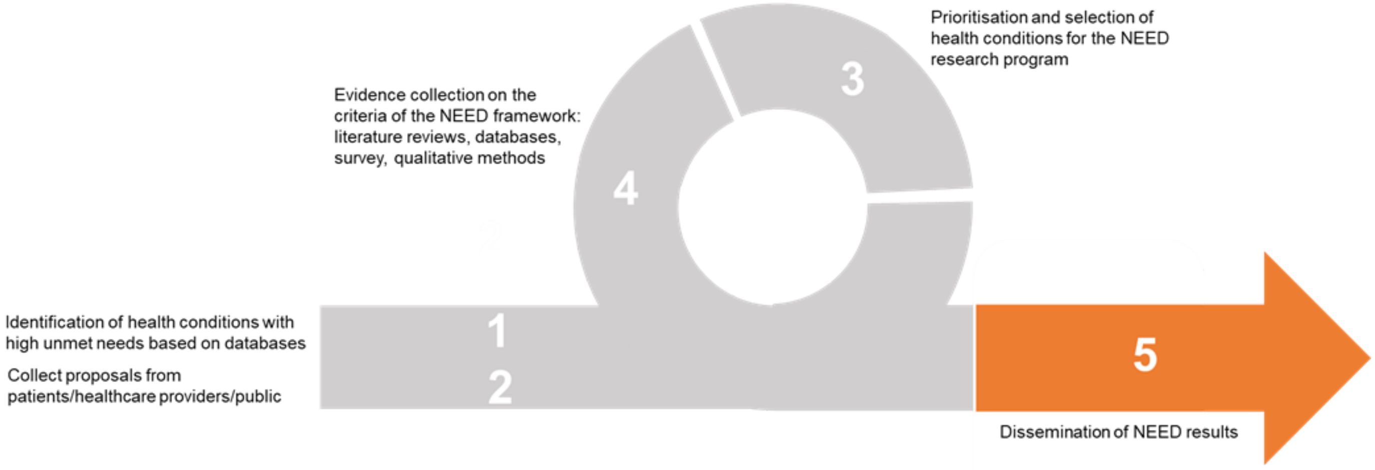

In your opinion, which other decision-making processes regarding rare diseases might be informed by the results of the NEED assessment?

What suggestions, if any, do you have on the dissemination of the results of the NEED assessment to ensure visibility?

To which specific stakeholders or organisations do you think the evidence derived from the NEED assessment should become available?

How, if even, do you suggest to keep the NEED database up-to-date?

Powered by Qualtrics
