# Supplementary material for: Criteria to evaluate unmet health-related needs of persons living with rare diseases and their caregivers: rapid literature review and stakeholder consultations
Source: Orphanet J Rare Dis. 2025 Jul 1;20:321. doi: 10.1186/s13023-025-03838-6 (PMC12211369; doi:10.1186/s13023-025-03838-6)
Supplement: Supplementary file 4 — Additional file 4. [file 13023_2025_3838_MOESM4_ESM.pdf]

Supplementary material 4: Consultation topic guide

1. Stakeholder consultation 1

Questions	Sub-questions	Aim
**Welcome and introduction**		
General description of the NEED approach		• To provide participants with a broad understanding of the research scope
Outline of the research objectives for workshop 1: *To assess the extent to which the NEED framework, along with its criteria and methods, is applicable to rare diseases*		• To clarify the subject of this in-depth discussion
**Dimension 1: Impact on patients – general**		
With respect to this dimension, what is your opinion on the proposed criteria and sub-criteria?	• Why are the highlighted sub-criteria so important?<br>• Why is it important to add the sub sub-criterion: **prevention**?<br>• Why is it important to add the sub sub-criterion: **impact on social life**?	• To obtain a general understanding of participants' perspectives regarding the overarching **"impact on patients"** dimension and its specific criteria and sub-criteria
**Dimension 1: Impact on patients – criteria and sub-criteria related to HEALTH needs**		
Do you have any recommendations on the sub-criteria included within this dimension?	• Why is it important to add the sub-criterion: **fatigue or exhaustion**?	• To assess the applicability of the criteria and sub-criteria within this dimension for rare diseases

	• Why is it important to add the sub-criterion: **aesthetic concerns**?<br>• Do you think the sub-criterion “**usual activities**” should be removed? Why?	
**Dimension 1: Impact on patients – criteria and sub-criteria related to HEALTHCARE needs**		
Do you have any recommendations on the sub-criteria included within this dimension?	• Why is it important to add the sub-criterion: **existence of a treatment**?<br>• Why is it important to add the sub-criterion: **treatment adherence**?<br>• Why is the criterion **“patient experience with healthcare professionals”** so important?<br>• Why is it important to add the following sub-sub criteria: i) **access to information**, ii) **access to diagnostic tests**, iii) **availability to disease experts?**<br>• Why is it important to add the follow sub sub-criteria: i) **number of mis-diagnosed and** ii) **number of specialists visited**?	• To assess the applicability of the criteria and sub-criteria within this dimension for rare diseases
**Dimension 1: impact on patients – criteria and sub-criteria related to SOCIAL needs**		
Do you have any recommendations on the sub-criteria included within this dimension?	• Why do you think the current criterion **“impact on work”** is to narrowly defined?<br>• Why is it important to add the sub-criterion: **re-integration after the disease**?	• To assess the applicability of the criteria and sub-criteria within this dimension for rare diseases
**Dimension 2: Impact on society – general**		

With respect to this dimension, what is your opinion on the proposed criteria and sub-criteria?	• Why are the highlighted (!) sub-criteria so important?<br>• Should the sub-criteria highlighted by a "?" be included within this domain?<br>• Why is it important to add the sub-criterion: **impact on patient organisations**?<br>• Why is it important to add the sub-criterion: **indirect costs for caregivers**?<br>• Why is it important to add the sub-criterion: **productivity gains**?	• To obtain a general understanding of participants' perspectives regarding the overarching **"impact on society"** dimension and its specific criteria and sub-criteria
**Dimension 2: Impact on society – criteria and sub-criteria related to HEALTH needs**		
Do you have any recommendations on the sub-criteria included within this dimension?	• What do you think of the criterion: **transmissibility**?<br>• What do you think of the criterion: **antimicrobial resistance**?<br>• Why is it important to add the sub-criterion: **social impact on caregivers**?	• To assess the applicability of the criteria and sub-criteria within this dimension for rare diseases
**Dimension 2: Impact on society – criteria and sub-criteria related to HEALTHCARE needs**		
Do you have any recommendations on the sub-criteria included within this dimension?		• To assess the applicability of the criteria and sub-criteria within this dimension for rare diseases
**Dimension 2: Impact on society – criteria and sub-criteria related to SOCIAL needs**		
Do you have any recommendations on the sub-		• To assess the applicability of the criteria and sub-criteria within this dimension for rare diseases

criteria included within this dimension?		
**Dimension 3: Impact on future generations – general**		
Do you have any recommendations on the sub-criteria included within this dimension?	• Why is it important to add the criterion: **speed of innovation uptake**?<br>• Why is it important to add the criterion: **impact on caregivers**?	• To assess the applicability of the criteria and sub-criteria within this dimension for rare diseases
**Methodological challenges**		
Overview of the different databases that can be used to identify rare health conditions with potential high unmet needs		• To inform participants on the different databases and which database will be used to measure a certain criterion or sub-criterion
What is your opinion on the use of databases in the context of rare diseases?	• What are or could be challenges related to this methodology?<br>• What are or could be potential mitigation strategies?	• To evaluate the appropriateness of the proposed methods in the NEED framework to assess the NEEDs criteria in the context of rare diseases
What is your opinion on the use of the BeBoD register in the context of rare diseases?		• To evaluate the adequacy of the BeBoD database to assess specific NEEDs criteria
**Methodological challenges – literature reviews**		
Overview of the different criteria and sub-criteria that can be measured by conducting a literature review		• To inform participants on which criteria and sub-criteria can be measures by conducting a literature review

What is your opinion on the use of literature reviews in the context of rare diseases?	• What are or could be challenges related to this methodology?<br>• What are or could be potential mitigation strategies?	• To evaluate the appropriateness of the proposed methods in the NEED framework to assess the NEEDs criteria in the context of rare diseases
**Methodological challenges – qualitative methods**		
Overview of the different criteria and sub-criteria that can be measured through qualitative methods		• To inform participants on which criteria and sub-criteria can be measures through qualitative research methods
What is your opinion on the use of qualitative methods in the context of rare diseases?	• What are or could be challenges related to this methodology?<br>• What are or could be potential mitigation strategies?	• To evaluate the appropriateness of the proposed methods in the NEED framework to assess the NEEDs criteria in the context of rare diseases
**Methodological challenges – surveys**		
Overview of the different criteria and sub-criteria that can be measured through quantitative methods		• To inform participants on which criteria and sub-criteria can be measures through quantitative research methods
What is your opinion on the use of quantitative methods in the context of rare diseases?	• What are or could be challenges related to this methodology?<br>• What are or could be potential mitigation strategies?	• To evaluate the appropriateness of the proposed methods in the NEED framework to assess the NEEDs criteria in the context of rare diseases
What are possible actions that can be taken to "**target the hard to reach**"?		• To evaluate the appropriateness of the proposed methods in the NEED framework to assess the NEEDs criteria in the context of rare diseases

What are possible actions that can be taken **to generalize findings from a national to an international level**?		• To evaluate the appropriateness of the proposed methods in the NEED framework to assess the NEEDs criteria in the context of rare diseases
What is your opinion on the use of the EQ-5D in the context of rare diseases?	• Are you aware of **other questionnaires that are better suited** to measure certain criteria or sub-criteria?	• To evaluate the appropriateness of the proposed methods in the NEED framework to assess the NEEDs criteria in the context of rare diseases
**Methodological challenges – multi methods**		
Overview of the different criteria and sub-criteria that can be measured using multiple methods		• To inform participants on which criteria and sub-criteria can be measures using multiple methods
**Closing remarks**		

2. Stakeholder consultation 2

Questions	Sub-questions	Aim
**Welcome and introduction**		
General description of the NEED approach		- To provide participants with a broad understanding of the research scope
Recap of workshop 1: *NEED assessment framework and its applicability in the context of rare health conditions*		- To outline the scope and key findings from the previous workshop
Outline of the research objectives for workshop 2: To *evaluate the 5-step model for the identification of patients' needs as a whole and its applicability to rare diseases*		- To clarify the subject of this in-depth discussion
Description of the 5-step model	• Identification of health conditions with high unmet needs based on databases **(step 1)**<br>• Open calls to collect proposals from patient organisations, HCPs and the general public **(step 2)**<br>• Prioritisation and selection of health conditions **(step 3)**<br>• Evidence collection on the criteria of the NEED framework **(step 4)**<br>• Dissemination of the NEED results **(step 5)**	- To inform participants on the objectives of the individual phases within the NEEDs 5-step implementation model
**General remarks on the 5-step implementation model**		

Do you have any general remarks regarding this 5-step implementation model and its applicability to rare diseases?		- To assess participants overall opinion on the 5-step model
Do you think certain steps of this proposed model should be adapted of added to make it applicable to the context of rare diseases?		- To assess the applicability of the 5-step implementation model in the context of rare diseases
**Step 1: Identification of health conditions with high unmet needs based on databases**		
In general, how do you think priority topics with potential high unmet needs should be identified in rare diseases?		- To identify methods that support the identification of rare health conditions
Do you have any recommendations regarding the use of databases for the identification of priority topics in the context of rare diseases?		- To gather participants' perspectives regarding the potential (dis)advantages of using databases to identify rare health conditions
**Step 2: Open calls to collect proposals from patient organisations, HCPs and the general public**		
What is your opinion on the open call for topics to identify priority topics with potential high unmet needs, in the context of rare diseases?	• Do you have any specific recommendations on how to perform the call for topics?	- To gather participants' perspectives regarding the potential (dis)advantages of using open calls to identify rare health conditions
What platforms/channels do you suggest for the dissemination of the call for topics?		- To identify platforms such that open calls can be broadly distributed

For common diseases, particular information items can be requested in the call for proposals, for instance the impact on quality of life, mortality, prevalence, inconveniences or availability of treatment?	• What is the minimal information that should be requested in the call for rare disease topic proposals to enable the prioritization of topics?	- To identify which information/topics should be requested in open calls
Should the information requested in the call for proposals be different for rare and common diseases?	• How should information items differ in view of rare diseases?	- To assess the differences in information requirements between common vs rare diseases
**Step 3: Prioritisation and selection of health conditions for the NEED research program**		
Outline of the KCE prioritisation process		
Should the prioritisation process be different or equal for common and rare health conditions?	• Why do you think that is important?<br>• In what way do you think the prioritisation process must be different?	- To assess whether common vs rare diseases require a different approach for prioritisation
Do you have any suggestions or remarks regarding the prioritisation criteria and their applicability in the context of rare diseases?		- To assess the applicability of the current prioiritisation criteria for rare diseases
**Step 4: Evidence collection on the criteria of the NEED framework**		
Do you have any recommendations or alternative methods for the NEED survey in case the number of patients living with the rare disease is very low?		- To identify general recommendations on how to tackle the problem of low disease prevalence within the context of rare diseases

Do you have any recommendations or remarks on the proposal to broaden the data gathering to the European level in the context of rare health conditions?		- To identify general recommendations on how to tackle the problem of low disease prevalence within the context of rare diseases
Do you have any ethical concerns relating to the conduct of a survey among patients in the context of rare diseases?		- To identify general recommendations on how to tackle the problem of low disease prevalence within the context of rare diseases
**Step 5: Dissemination of the NEED survey**		
What suggestions do you have on the dissemination of the results of the NEED assessment to ensure visibility?		- To identify the future use and application of the results
Which other decision-making processes regarding rare diseases might be informed by the results of the NEED assessment?	• How can these processes be informed?	- To identify the future use and application of the results
To which specific stakeholders or organisations do you think the evidence derived from the NEED assessment should become available		- To identify the future use and application of the results
How do you suggest keeping the NEED database up to date?		- To identify the future use and application of the results
**Closing remarks**		
