# Supplementary material for: Criteria to evaluate unmet health-related needs of persons living with rare diseases and their caregivers: rapid literature review and stakeholder consultations
Source: Orphanet J Rare Dis. 2025 Jul 1;20:321. doi: 10.1186/s13023-025-03838-6 (PMC12211369; doi:10.1186/s13023-025-03838-6)
Supplement: Supplementary file 5 — Additional file 5. [file 13023_2025_3838_MOESM5_ESM.pdf]

Supplementary material 5: Article characteristics

Overview of identified articles including an evaluation of needs in rare diseases

Study characteristics	Number of included articles (N = 272)
**Year of publication**	
2006 – 2009	5
2010 – 2013	8
2014 – 2017	33
2018 – 2021	121
2022 – 2023	105
**Geographical region**	
Europe	135
US	44
Australia	12
Canada	5
Latin-America	3
Asia	8
Inter-continental	30
Unspecificed	35
**Study design**	
Literature review	31
Qualitative methods	70
Quantitative methods	129
Mixed quantitative-qualitative methods	28
Other	14
**Disease categorisation (ICD-11)**	

**Orphan but non-disease specific**	56
**Disease specific**	213
ICD01 - Certain infection diseases	2
ICD02 - Neoplasms	13
ICD03 - Diseases of the blood or blood-forming organs	11
ICD04 - Diseases of the immune system	22
ICD05 - Endocrine, nutritional or metabolic diseases	62
ICD07 - Sleep/wake disorders	1
ICD08 - Diseases of the nervous system	31
ICD09 - Diseases of the visual system	1
ICD11 - Diseases of the circulatory system	3
ICD12 - Diseases of the respiratory system	4
ICD13 - Diseases of the digestive system	4
ICD14 - Diseases of the skin	6
ICD15 - Diseases of the musculoskeletal system or connective tissue	8
ICD16 - Diseases of the genitourinary system	2
ICD18 - Pregnancy, childbirth or the puerperium	1
ICD20 - Development anomalies	35
X - Extension Codes	7
**Not specified**	3

Summary of identified article characteristics including an evaluation of needs in rare diseases categorized according to method category

1. Literature based studies

Author (year)	Geographical region	Disease	Study design: Method	Study design: Objectives	NEED criteria
**Review**					
**Wehrli (2023)**	Unspecified	Osteogenesis imperfecta		Improved physical healthof paediatric and adult patients	- Improved mental health needs<br>- Support in work and education
**Choon (2023)**	Unspecified	Generalised pustular psoriasis	*Systematic literature review*	Health-related Improved physical health	- Improved physical health<br>- Improved mental health<br>- Availability of Availability of treatments<br>- Social support
**Wechsler (2023)**	Unspecified	hypereosinophilic syndrome and eosinophilic granulomatosis with polyangiitis		Unmet needs and evidence gaps	- Improved physical health<br>- Availability of Availability of treatments<br>- Timely and accurate diagnosis<br>- Information needs
**Fournier (2023)**	Europe	Rare genetic skin diseases	*Systematic literature review*	Psychosocial implications	- Improved physical health<br>- Improved mental health<br>- Improved autonomy<br>- Accessibility of healthcare<br>- Social support<br>- Support in work and education
**Connolly (2023)**	Australia	Sarcoma			- Improved physical health
**Howley (2023)**	Europe	Congenital athymia			- Financial stability<br>- Improved mental health<br>- Integrated, multi-disciplinary care<br>- Information needs<br>- Accessibility of healthcare<br>- Timely and accurate diagnosis
**Applequist (2023)**	US	Orphan but non disease specific		Direct-to-Consumer recruitment methods	
**Dabbous (2023)**	Inter-continental	Orphan but non disease specific	*Systematic literature review*	Valuation of Availability of treatments of RD	- Improved physical health<br>- Financial stability
**McMullan (2022)**	Unspecified	Rare diseases		Needs of informal caregivers	- Improved physical health<br>- Financial stability
**Sawad (2022)**	US	Arginase 1 deficiency (ARG1-D)	*Systematic review of case reports*	Unmet needs of patients	- Improved physical health<br>- Availability of treatments<br>- Timely and accurate diagnosis
**Trieste (2022)**	Europe	Rare and complex connective tissue and musculoskeletal diseases (rCTDs)		Assessing Improved physical health	

**Brandt (2022)**	Inter-continental	Spinal muscular atrophy	*Systematic review of quantitative and qualitative data*	Psychosocial situation, caregiver burden and family needs	- Improved physical health<br>- Financial stability<br>- Integrated, multi-disciplinary care<br>- Information needs<br>- Social support
**Hassall (2022)**	Europe	Lysosomal storage disorders (LSDs)	*Systematic review and integrative sequential explanatory narrative synthesis*	Psychosocial impact of parenting a patient	- Improved physical health<br>- Financial stability<br>- Availability of treatments<br>- Timely and accurate diagnosis
**Camerini (2022)**	Europe	Acromegaly	*Systematic review*	Clinical powerful instruments in Timely and accurate diagnosis and management	- Availability of Availability of treatments<br>- Availability of Availability of treatments
**Bardon (2022)**	Inter-continental	Orphan but non disease specific	*Realist review of literature*	Development, evaluation and implementation of evidence-based group therapy	- Social support
**De Heus (2021)**	Europe	Rare cancer	*Systematic review*	Unmet supportive care needs	- Improved physical health<br>- Timely and accurate diagnosis<br>- Social support
**Soeun (2021)**	US	Immunoglobulin A nephropathy	*Systematic literature review*	Epidemiology, HRImproved physical healthand Financial stability	- Financial stability<br>- Improved physical health<br>- Improved mental health<br>- Availability of treatments<br>- Timely and accurate diagnosis
**Seefried (2021)**	Unspecified	X-linked hypophosphataemia	*Systematic literature review*	Improved physical health	- Improved physical health<br>- Improved mental health<br>- Improved autonomy<br>- Availability of treatments<br>- Accessibility of healthcare<br>- Impact on work
**Petros (2021)**	US	Adult-onset Still's disease		Clinical manifestation, Timely and accurate diagnosis, Availability of treatments, and unmet needs	- Accessibility of healthcare<br>- Timely and accurate diagnosis<br>- Social support
**Monga (2020)**	Inter-continental	Mantle cell lymphoma	*Systematic literature review*	Real-world clinical, economic and humanistic burden data	- Financial stability<br>- Improved physical health<br>- Availability of treatments<br>- Timely and accurate diagnosis
**Lanar (2020)**	Europe	Orphan but non disease specific		To identify all relevant PROMs	
**Hill (2019)**	Europe	Osteogenesis imperfecta	*Systematic review*	Experiences and psychosocial impact on families and caregivers	- Improved physical health<br>- Financial stability<br>- Improved mental health<br>- Improved autonomy<br>- Information needs<br>- Accessibility of healthcare
**Mackay (2019)**	Unspecified	Prader-Willi syndrome		Describing health and well-being	- Improved physical health<br>- Improved physical health<br>- Impact on work

**Meyer (2019)**	Unspecified	Idiopathic inflammatory myopathies	*Narrative review Systematic literature search*	Unmet needs in clinical practice guidelines	- Accessibility of healthcare
**Palikara (2018)**	Unspecified	Williams syndrome		Educational needs	- Improved physical health<br>- Improved autonomy<br>- Information needs<br>- Impact on work and education
**Wheeler (2017)**	US	Angelman syndrome	*Targeted literature review*	AS phenotype, epidemiology, diagnostic processes, Availability of treatments and burden	- Improved physical health<br>- Improved mental health<br>- Availability of treatments<br>- Timely and accurate diagnosis<br>- Social support
**Yamaguchi (2017)**	Asia	Inborn errors of metabolism		Difficulties in Daily Life and Associated Factors and Improved physical health	- Improved physical health<br>- Improved mental health<br>- Accessibility of healthcare<br>- Financial stability
**Pelentsov (2015)**	Australia	Orphan but non disease specific	*Scoping review*	Supportive care needs of parents	- Improved physical health<br>- Financial stability<br>- Information needs<br>- Accessibility of healthcare<br>- Social support
**Banerji (2013)**	US	Hereditary angioedema	*Boolean search*	Burden of ilness	- Improved physical health<br>- Financial stability<br>- Improved mental health<br>- Improved autonomy<br>- Accessibility of healthcare<br>- Social support
**Rajmil (2010)**	Unspecified	Orphan but non disease specific	*Systematic review*	Available instruments of HRImproved physical health	
**Gaite (2008)**	Europe	Orphan but non disease specific		Needs assessment	- Improved physical health<br>- Financial stability<br>- Integrated, multi-disciplinary care<br>- Information needs<br>- Timely and accurate diagnosis<br>- Social support

2. Quantitative studies

Author (year)	Geographical region	Disease	Study desgin		NEED criteria
			Population	*Validated tool*	
**Survey/Questionnaire**					
**Schut (2023)**	Europe	Desmoid- type fibromatosis (DTF)	- Patients/patient organisations/patient representatives	- SQ20<br>- EORTC QLQ-C30	- Improved physical health<br>- Integrated, multi-disciplinary care<br>- Accessibility to healthcare
**Landlust (2023)**	Inter-continental	Phelan-McDermid syndrome (PMS)	- Caregiver/parents	- Genetic Syndrome Stressors Scale (GSSS)	- Improved physical health
**Taruscio (2023)**	Inter-continental	Undiagnosed diseases	- Multistakeholder		- Integrated, multi-disciplinary care<br>- Information needs<br>- Timely and accurate diagnosis
**Goldfarb (2023)**	US	Primary hyperoxaluria	- Patients and caregivers	- Kidney Disease Quality of Life (KDIMPROVED PHYSICAL HEALTH-36™)<br>- Wisconsin Stone Quality of Life (WISIMPROVED PHYSICAL HEALTH)	- Availability of treatments<br>- Timely and accurate diagnosis
**Skrobanski (2023)**	Europe	tuberous sclerosis complex	- Caregiver		
**Moore (2023)**	Inter-continental	Pelizaeus-Merzbacher Disease	- Caregiver/parent		- Improved physical health<br>- Availability of treatments<br>- Timely and accurate diagnosis
**Stubbs (2023)**	US	juvenile localized and systemic scleroderma	- Caregiver	- The Barriers to Care Scale (BACS)	- Availability of treatments<br>- Integrated, multi-disciplinary care<br>- Accessibility to healthcare<br>- Timely and accurate diagnosis
**Acaster (2023)**	US	Hereditary transthyretin amyloidosis	- Caregiver/parents	- EQ-5D-3L<br>- Hospital anxiety and depression scale	- Improved physical health
**Tkachuk (2023)**	Latin-America	neuromyelitis optica spectrum disorders	- Patients/patient organisations/patient representatives	- EDSS	- Accessibility to healthcare<br>- Timely and accurate diagnosis
**Alesci (2023)**	Europe	Immune thrombocytopenia	- Patients/patient organisations/patient representatives	- FACIT-F<br>- BDI	- Improved physical health

				- Epworth Sleepiness Scale<br>- STOP-BANG	
**Ballesteros-Sayas (2023)**	Europe	Dravet syndrome	- Caregiver/parents		- Timely and accurate diagnosis<br>- Accessibility of healthcare<br>- Support in work and education<br>- Financial stability
**Büttner (2023)**	Europe	Hypoparathyriodism	- Patients/patient organisations/patient representatives	- HPQ-28	- Improved physical health<br>- Information needs<br>- Impact on work
**Kerr (2023)**	US	complex vascular anomalies	- Patients/patient organisations/patient representatives	- PROMIS Global Health Measure<br>- PCC-Ca-36	- Improved physical health<br>- Integrated, multi-disciplinary care<br>- Information needs<br>- Accessibility of healthcare<br>- Social support
**Chappell (2023)**	Unspecified	mtDNA deletion syndromes	- Patient and caregivers	- MCN-PNS<br>- ZBI-22	- Improved physical health<br>- Financial stability<br>- Integrated, multi-disciplinary care<br>- Information needs
**Mahic (2023)**	Europe	myasthenia gravis	- HCPs	- WPAI<br>- MG-Improved physical health-15r<br>- EQ-5D-5L<br>- VAS	- Improved physical health<br>- Availability of treatments<br>- Integrated, multi-disciplinary care
**Pivonello (2023)**	Europe	Cushing's syndrome	- HCPs		- Availability of treatments
**Runacres (2023)**	Australia	Motor neuron disease	- Patients	- ALSFRS-R<br>- IPOS-Neuro questionnaire	- Improved physical health
**Van Roessel (2023)**	Europe	hypothalamic dysfunction	- Patients/patient organisations/patient representatives		- Improved physical health<br>- Integrated, multi-disciplinary care<br>- Timely and accurate diagnosis<br>- Information needs<br>- Financial stability
**Weiss (2023)**	Unspecified	antisynthetase syndrome	- Patients/patient organisations/patient representatives<br>- HCP		- Timely and accurate diagnosis<br>- Information needs<br>- Impact on work
**Schuster (2022)**	Europe	Duchenne Muscular Dystrophy	- Patients & caregivers	- PUL-PROM<br>- EQ-5D-3L	- Improved physical health<br>- Availability of treatments
**Pélissier (2022)**	Europe	Non-disease specific	- Patients & caregivers		- Timely and accurate diagnosis
**Strzelczyk (2022)**	Europe	Dravet syndrome	- Patients & caregivers		
**Gutenbrunner (2022)**	Europe	Non-disease specific	- Patients/patient organisations/patient representatives	- ReGoS	- Improved physical health<br>- Integrated, multi-disciplinary care<br>- Accessibility to healthcare

**Gimenez-Lozano (2022)**	Europe	Non-disease specific	- Patients & caregivers		- Improved physical health<br>- Availability of treatments<br>- Accessibility to healthcare<br>- Timely and accurate diagnosis
**Valassi (2022)**	Inter-continental	Cushing's syndrome	- Patients & caregivers		- Improved physical health<br>- Availability of treatments<br>- Integrated, multi-disciplinary care
**Kramer-Golinkoff (2022)**	US	Cystic fibrosis (CF)	- Patients & caregivers		- Improved physical health<br>- Availability of treatments<br>- Accessibility to healthcare
**Kontogeorgos (2022)**	Europe	Hypoparathyroidism	- Patients/patient organisations/patient representatives	- SF-36<br>- EQ5D-VAS	- Improved physical health<br>- Accessibility to healthcare
**Bonnekoh (2022)**	Europe	Urticarial vasculitis	- Patients/patient organisations/patient representatives		- Availability of treatments
**Johansen (2022)**	Europe	Loeys-Dietz syndromes, vascular Ehlers-Danlos syndrome	- Patients/patient organisations/patient representatives	- Standardized Nordic Questionnaire<br>- FSS<br>- HADS	- Improved physical health<br>- Integrated, multi-disciplinary care<br>- Accessibility to healthcare
**Rihm (2022)**	Europe	Non-disease specific	- Caregiver/parents	- DT-P	- Improved physical health<br>- Availability of treatments<br>- Integrated, multi-disciplinary care
**Scharping (2022)**	Europe	Inherited metabolic diseases (IMDs)	- Caregiver/parents	- PNS	- Integrated, multi-disciplinary care<br>- Accessibility to healthcare
**Mancuso (2022)**	Europe	Rare neurological diseases (RNDs)	- HCP		- Accessibility to healthcare<br>- Timely and accurate diagnosis
**Mahic (2022)**	US	Myasthenia gravis	- HCP		- Improved physical health<br>- Availability of treatments<br>- Timely and accurate diagnosis
**Al Mukaddam (2022)**	Inter-continental	Fibrodysplasia ossificans progressiva	- Patients & caregivers		- Improved physical health<br>- Availability of treatments<br>- Accessibility to healthcare
**Mesa (2022)**	US	Systemic mastocytosis (SM)	- Patients/patient organisations/patient representatives		- Improved physical health
**Mesa (2022)**	US	Systemic mastocytosis (SM)	- HCP		- Improved physical health<br>- Availability of treatments
**Auchus (2022)**	Inter-continental	Congenital adrenal hyperplasia	- HCP		- Improved physical health<br>- Availability of treatments
**Rispoli (2022)**	Europe	Dystonia	- Patients/patient organisations/patient representatives		- Improved physical health<br>- Integrated, multi-disciplinary care
**Koto (2022)**	Asia	Fabry disease	- Patients/patient organisations/patient representatives	- SF-8	- Improved physical health<br>- Timely and accurate diagnosis
**Atila (2022)**	Europe	Central diabetes insipidus	- Patients/patient organisations/patient representatives		- Improved physical health<br>- Integrated, multi-disciplinary care
**Bogart (2022)**	US	Orphan but non disease specific	- Patients & caregivers		- Improved physical health<br>- Integrated, multi-disciplinary care<br>- Timely and accurate diagnosis<br>- Information needs

					- Accessibility of healthcare<br>- Social support<br>- Financial stability
**Moretti (2021)**	Europe	Non-disease specific	- Patients & caregivers	- Checklist For Children with Special Healthcare Needs	- Improved physical health<br>- Accessibility to healthcare
**Strober (2021)**	NS	Generalized pustular psoriasis (GPP)	- HCP		- Availability of treatments
**Bryson (2021)**	Inter-continental	Non-disease specific	- Patients/patient organisations/patient representatives		- Improved physical health<br>- Integrated, multi-disciplinary care
**Drabbe (2021)**	Europe	Non-disease specific	- Patients/patient organisations/patient representatives		- Integrated, multi-disciplinary care<br>- Timely and accurate diagnosis
**Lamy (2021)**	Europe	Non-disease specific	- Patients/patient organisations/patient representatives		- Integrated, multi-disciplinary care<br>- Accessibility to healthcare<br>- Timely and accurate diagnosis
**Rose (2021)**	NS	relapsing polychondritis	- Patients & HCP	- SF-36<br>- MFI	- Improved physical health<br>- Availability of treatments<br>- Integrated, multi-disciplinary care<br>- Timely and accurate diagnosis
**McMillan (2021)**	NS	Spinal Muscular Atrophy (SMA)	- Patients & caregivers	- EQ-5D-5L<br>- CSI	- Improved physical health
**Mendivil (2021)**	Inter-continental	hereditary angioedema	- Patients/patient organisations/patient representatives	- AE-Improved physical health<br>- SF-12v2<br>- AECT<br>- HADS<br>- WPAI	- Improved physical health<br>- Availability of treatments
**Langhinrichsen-Rohling (2021)**	US	EhlersDanlos Syndrome (EDS)	- Patients/patient organisations/patient representatives		- Improved physical health<br>- Integrated, multi-disciplinary care<br>- Timely and accurate diagnosis
**de Graaf (2021)**	Europe	Non-disease specific	- Patients/patient organisations/patient representatives	- CarerImproved physical health-7D<br>- WPAI<br>- 5-D-Itch scale<br>-	- Improved physical health<br>- Availability of treatments<br>- Integrated, multi-disciplinary care
**Ruiz-Casas (2021)**	Europe	progressive familial intrahepatic cholestasis	- Caregiver & HCP	- PedsQL™<br>- FIM<br>- VSS<br>- GMFC-MLD<br>- HRIMPROVED PHYSICAL HEALTH<br>- Family Functioning	

				- Summary Scores<br>- Total Impact Score	
**AmmannSchnell (2021)**	Europe	severe neurological disorders, including Pontocerebellar hypoplasia type 2 (PCH2) and metachromatic leukodystrophy (MLD)	- Caregiver/parents		
**Fasshauer (2021)**	Europe	Primary immunodeficiencies	- Patients/patient organisations/patient representatives		- Improved physical health
**Eichler (2021)**	Europe	Gastrointestinal stromal tumours	- Patients/patient organisations/patient representatives	- EORTC QLQ-C30	- Improved physical health<br>- Availability of treatments<br>-
**Depping (2021)**	Europe	Non-disease specific	- Patients/patient organisations/patient representatives	- SCNS-SF34	- Improved physical health<br>- Integrated, multi-disciplinary care
**Cheung (2021)**	Inter-continental	X-linked hypophosphatemia	- Patients & caregivers	- XLH Improved physical health	- Improved physical health<br>- Availability of treatments<br>- Integrated, multi-disciplinary care
**Grant (2021)**	US	mucopolysaccharidoses	- Family members		
**Webb (2021)**	Inter-continental	Non-disease specific	- Patients & HCP		- Timely and accurate diagnosis
**Sestini (2021)**	Europe	Inherited Metabolic Diseases	- Patient & caregiver		- Improved physical health<br>- Integrated, multi-disciplinary care<br>- Accessibility to healthcare
**Qi X (2021)**	Asia	Gaucher Disease	- Patient & caregiver	- PSQI<br>- SG-36	- Improved physical health<br>- Integrated, multi-disciplinary care<br>- Accessibility to healthcare<br>- Timely and accurate diagnosis
**Abeni (2021)**	Europe	Autosomal recessive congenital ichthyoses	- Patients/patient organisations/patient representatives<br>-	- FDLQI<br>- FBI tool	- Improved physical health<br>- Financial stability
**Bejarano (2021)**	Unspecified	hATTR amyloidosis	- Patients/patient organisations/patient representatives	- IEXPAC questionnaire	- Integrated, multi-disciplinary care<br>- Impact on work
**Molnár (2021)**	Europe	Pompe disease	- Patients/patient organisations/patient representatives	- Fatigue Severity Score<br>- R-PAct-Scale<br>- Rotterdam and Bartel disability scale<br>- EQ-5D<br>- SG-36	- Improved physical health<br>- Information needs
**Martins (2020)**	Latin-Amerika	Phenylketonuria (PKU)	- Patients & caregivers		- Improved physical health<br>- Integrated, multi-disciplinary care<br>- Timely and accurate diagnosis

**Painous (2020)**	Europe	Four different groups of rare MDs were studied: group 1 included dystonia, paroxysmal dyskinesia and neurodegeneration with brain iron accumulation (NBIA) (group 1-DYS); group 2, ataxias and hereditary spastic paraparesis (HSP) (group 2-ATX/ HSP); group 3, atypical parkinsonism such as progressive supranuclear palsy, multiple system atrophy and corticobasal degeneration (group 3-AP); group 4 included Huntington's disease (HD) and other choreas (group 4-C).	- HCPs		- Availability of treatments<br>- Accessibility to healthcare<br>- Timely and accurate diagnosis
**Dermer (2020)**	Canada	Genetically determined leukoencephalopathies	- Caregiver/parents	- Parenting Stress Index– 4th Edition	- Timely and accurate diagnosis
**Izquierdo-Garcíaa (2020)**	Europe	Hereditary fructose intolerance	- Patients & caregivers	- EQ-5D-Y<br>- ED-5D	- Improved physical health<br>- Integrated, multi-disciplinary care<br>- Accessibility to healthcare<br>- Timely and accurate diagnosis
**Bolte (2020)**	Europe	Transthyretin familial amyloid polyneuropathy (ATTRFAP)	- Patients/patient organisations/patient representatives	- PROMIS-10<br>- WHODAS 2.0	- Improved physical health<br>- Accessibility to healthcare
**Xu (2021)**	Asia	Gaucher Disease	- Caregiver	- SF- 36<br>- SAS<br>- SDS<br>- Multi-dimensional Scale of Perceived Social support	
**Boettcher (2020)**	Europe	Non-disease specific	- Caregiver/parents	- Improved physical health<br>- ULQIE<br>- BSI<br>- CHIP<br>- OSSS-3<br>- FAM	

**Fjermestad (2020)**	Europe	neurofibromatosis 1 (NF1) and hereditary spastic paraparesis (HSP)	- Patients/patient organisations/patient representatives		- Integrated, multi-disciplinary care
**Saglio (2020)**	Europe	Narcolepsy and idiopathic hypersomnia	- HCP		
**Cook (2020)**	Inter-continental	Pigmented villonodular synovitis	- Patients/patient organisations/patient representatives		- Improved physical health<br>- Availability of treatments<br>- Integrated, multi-disciplinary care<br>- Accessibility to healthcare<br>- Timely and accurate diagnosis
**Dinur (2020)**	Inter-continental	Gaucher disease	- Patients/patient organisations/patient representatives	- GD1-PROM	- Improved physical health<br>- Availability of treatments
**Kodra (2020)**	Europe	Cri du Chat syndrome (CdC)	- Patients & caregiver		- Improved physical health<br>- Availability of treatments<br>-
**Kuman (2020)**	Europe	- homozygous familial hypercholesterolemia	- Patients/patient organisations/patient representatives	- SCL-90-R	- Improved physical health
**Valero-Moreno (2020)**	Unspecified	- Primary ciliary dyskinesia	- Patients/patient organisations/patient representatives	- BIEPS-J<br>- HADS-A	- Improved physical health
**Khair (2019)**	Europe	Inherited bleeding disorders	- Caregiver/parents	- PNS-RD	- Integrated, multi-disciplinary care
**Lagaea (2019)**	Europe	Dravet syndrome (DS)	- Patients/patient organisations/patient representatives		- Improved physical health<br>- Availability of treatments<br>- Accessibility to healthcare
**Efthymiadou (2019)**	Europe	Non-disease specific	- Patients/patient organisations/patient representatives		- Integrated, multi-disciplinary care
**Courbier (2019)**	Inter-continental	Non-disease specific	- Patients & caregivers		- Integrated, multi-disciplinary care
**Conner (2019)**	US	Mucopolysaccharidosis	- Caregiver/parents		- Improved physical health<br>- Availability of treatments<br>- Accessibility to healthcare
**Nagamatsu (2019)**	Asia	malignant pleural mesothelioma (MPM)	- Patients/patient organisations/patient representatives		- Improved physical health<br>- Availability of treatments<br>- Integrated, multi-disciplinary care<br>- Accessibility to healthcare
**Morrison (2019)**	Europe	Mucopolysaccharidosis type VII	- Patients/patient organisations/patient representatives		- Improved physical health<br>- Improved physical health<br>- Timely and accurate diagnosis<br>- Social support<br>- Support in work and education
**Godoy Torres (2018)**	Latin-Amerika	Chronic kidney disease (CKD) in children	- Caregiver/parents	- CBS<br>- BDI<br>- BAI	- Improved physical health<br>- Accessibility to healthcare
**Ebert (2018)**	Europe	anorectal malformations (ARM) and the exstrophy-	- Patients/patient organisations/patient representatives		- Improved physical health

		epispadias complex (EEC)			
**Fischer (2020)**	Europe	spinal muscular atrophy	- Patients/patient organisations/patient representatives	- SWLS<br>- RSES<br>- PANAS<br>- USER-P<br>- BPNSFS	- Improved physical health<br>- Availability of treatments
**Efthymiadou (2018)**	Europe	Non-disease specific	- Patients/patient organisations/patient representatives		- Improved physical health
**Pinto (2018)**	Europe	hemophilia (PWH)	- Caregiver/parents	- Sociodemographic and Clinical Questionnaires<br>- Improved physical healthActivity Questionnaire<br>- A36 Hemofilia-Improved physical health:15<br>- CHO-KLAT<br>- HAL<br>- PedHAL<br>- PROMIS-Anxiety and Depression<br>- IPQ-R	- Improved physical health
**Majoor (2017)**	Europe	Fibrous dysplasia (FD)	- Patients/patient organisations/patient representatives	- EQ-5D	- Improved physical health
**van Walsem (2017)**	Europe	Huntington's disease	- Patients/patient organisations/patient representatives	- EQ-5D-3L<br>- NPCS<br>- UHDRS	- Improved physical health<br>- Availability of treatments
**Lakhani Jindal (2017)**	Canada	Hereditary angioedema	- Patients/patient organisations/patient representatives	- SF-36v2	
**Dzemaili (2017)**	Inter-continental	congenital hypogonadotropic hypogonadism (CHH)	- Patients/patient organisations/patient representatives	- MMAS<br>- SDS<br>- IPQ-R	- Improved physical health<br>- Availability of treatments<br>- Integrated, multi-disciplinary care<br>- Accessibility to healthcare<br>- Timely and accurate diagnosis
**Wolin (2017)**	US	Neuroendocrine Tumors	- Patients/patient organisations/patient representatives		- Improved physical health<br>- Availability of treatments<br>- Integrated, multi-disciplinary care<br>- Timely and accurate diagnosis<br>- Accessibility of healthcare<br>- Impact on work<br>- Financial stability

**Angelis (2016)**	Europe	Juvenile idiopathic arthritis (group of different rare disorders)	- Patients & caregivers	- EQ-5D<br>- Barthel Index	
**Molster (2016)**	Australia	Non-disease specific	- Patients & caregivers		- Integrated, multi-disciplinary care<br>- Accessibility to healthcare<br>- Timely and accurate diagnosis
**Landfeldt (2016)**	Europe	Duchenne muscular dystrophy (DMD)	- Caregivers/parents		
**Silibello (2016)**	Europe	Non-disease specific	- Caregivers		- Improved physical health<br>- Integrated, multi-disciplinary care
**Pelentsov (2016)**	Australia	Non-disease specific	- Caregivers/parents		- Integrated, multi-disciplinary care<br>- Accessibility to healthcare
**Pelentsov (2016)**	Australia	Non-disease specific	- Caregivers/parents		
**Siden (2015)**	Canada	Non-disease specific	- Patients & caregivers		- Improved physical health
**Chevreul (2015)**	Europe	Systemic sclerosis	- Patients & caregivers	- EQ-5D-5L	
**Fang (2015)**	Asia	Primary cutaneous amyloidosis	- Patients/patient organisations/patient representatives	- DLQI<br>- SF-36	- Improved physical health
**Garcia (2015)**	Europe	Orphan but non disease specific	- Patients/patient organisations/patient representatives		- Timely and accurate diagnosis<br>- Information needs<br>- Social support<br>- Financial stability
**Kocova (2014)**	Europe	Spinal muscular atrophy	- Patients & caregivers	- PedsQL Neuromuscular Module Scales	- Integrated, multi-disciplinary care
**Anderson (2013)**	Australia	Non-disease specific	- Patients & caregivers	- HUI- II<br>- IOF<br>- RAHC-MOF	- Improved physical health<br>- Accessibility to healthcare<br>- Timely and accurate diagnosis
**Guillevin (2013)**	Europe	Pulmonary arterial hypertension	Patients & caregivers		- Improved physical health<br>- Improved physical health<br>- Information needs<br>- Social support<br>- Impact on work
**Morad (2007)**	Canada	ocular genetic eye disease	- Patients/patient organisations/patient representatives		- Integrated, multi-disciplinary care<br>- Accessibility to healthcare
**Database analysis**					
**Bell (2023)**	US	Eosinophilic granulomatosis with polyangiitis	- Patients/patient organisations/patient representatives		
**Benito-Lozano (2023)**	Europe	Orphan but non disease specific	- Patients/patient organisations/patient representatives		- Improved physical health<br>- Timely and accurate diagnosis<br>- Social support<br>- Support in work and education
**Strobel (2022)**	Inter-continental	Rare Eosinophil-Driven Diseases			- Improved physical health<br>- Accessibility to healthcare

				- Timely and accurate diagnosis
**Tisdale (2021)**	US		- Patients/patient organisations/patient representatives	- Improved physical health<br>- Accessibility to healthcare<br>- Timely and accurate diagnosis
**Hilker (2021)**	Europe	Sarcoidosis or Boeck's disease	-	- Integrated, multi-disciplinary care
**Burke (2021)**	US	Hemophilia B	- Patients/patient organisations/patient representatives	- Improved physical health
**Carbone (2020)**	Europe	Primary sclerosing cholangitis	- Patients/patient organisations/patient representatives	- Integrated, multi-disciplinary care
**Meng (2019)**	Europe		- - Patients & caregivers	- Integrated, multi-disciplinary care<br>- Accessibility to healthcare<br>- Timely and accurate diagnosis
**Pujades-Rodriguez (2018)**	Unspecified	hypertrophic cardiomyopathy	- Patients/patient organisations/patient representatives	- Improved physical health<br>-<br>- Availability of treatments
**Registries**				
**Palladini (2023)**	Europe	light chain (AL) amyloidosis	- Patients/patient organisations/patient representatives	- Availability of treatments<br>- Timely and accurate diagnosis
**Senn (2023)**	Europe	Inclusion Body Myositis	- Patients/patient organisations/patient representatives	- Improved physical health<br>- Integrated, multi-disciplinary care
**Van Geest (2023)**	Unspecified	MCT8 Deficiency	- Patients/patient organisations/patient representatives	- Integrated, multi-disciplinary care<br>- Timely and accurate diagnosis
**Duffy (2021)**	US	WAGR syndrome	- Patients/patient organisations/patient representatives	- Improved physical health

**Duda (2020)**	Europe	Hemophilia	- Patients/patient organisations/patient representatives		
**Kolbin (2020)**	Europe	Spinal muscular atrophies	- Patients/patient organisations/patient representatives		
**Tilson (2013)**	Europe	Cryopyrin-Associated Periodic Syndromes (CAPS)	- Patients/patient organisations/patient representatives		
**MCDA**					
**Gasol (2022)**	Europe	Non-disease specific	- Multistakeholder		
**Cross-sectional studies**					
**Burgevin (2022)**	Europe	Silver-Russell syndrome	- Patients/patient organisations/patient representatives		- Improved physical health<br>- Social support
**Clairman (2021)**	Unspecified	Juvenile dermatomyositis	- Patients/patient organisations/patient representatives		
**Witt (2019)**	Europe	achondroplasia	- Patients & HCP		- Improved physical health<br>- Integrated, multi-disciplinary care

3. Qualitative studies

Author (year)	Geographical region	Disease	Study design		NEED criteria
			**Population**	***Objective***	
**Focus group discussions**					
**Snijders (2023)**	Europe	Autoimmune hepatitis	Multistakeholder		- Integrated, multi-disciplinary care<br>- Information needs
**Witt (2023)**	Unspecified	Orphan but non disease specific	Multistakeholder	Obstacles and barriers in route to psychosocial care	- Integrated, multi-disciplinary care<br>- Information needs
**Beirne (2023)**	Europe	Hyperemesis Gravidarum	Patients	Healthcare experience	- Improved mental health<br>- Accessibility of healthcare<br>- Social support<br>- Financial stability
**Dreyer (2022)**	US	Kallmann syndrome	Patients & caregivers	In person vs virtual focus groups	- Timely and accurate diagnosis<br>- Social support
**Pignolo (2022)**	Inter-continental	Fibrodysplasia ossificans progressive	Multistakeholder	Unmet needs	- Availability of treatments<br>- Integrated, multi-disciplinary care<br>- Information needs

					- Accessibility of healthcare<br>- Timely and accurate diagnosis<br>- Social support
**Petracca (2021)**	Europe	Type A Hemophilia	Multistakeholder	Input for app improvement	
**Kleinendorst (2020)[1]**	Europe	16p11.2 deletion	Patients & caregivers	Impact on life and information provision	- Improved physical health<br>- Financial stability<br>- Improved mental health<br>- Improved autonomy<br>- Integrated, multi-disciplinary care<br>- Information needs<br>- Social support
**Aiyegbusi (2020)**	Europe	Renal transplant recipients and primary sclerosing cholangitis patients	Patients & HCP	Impact of rare disease on patients	- Future public health trends<br>- Improved physical health
**Pak (2020)**	Unspecified	Acromegaly	Patients	Patient perceptions of primary to tertiary care	- Integrated, multi-disciplinary care<br>- Information needs<br>- Social support
**O'Mahony (2018)**	Global	Haemophilia	Multistakeholder	Patient impacts	- Improved physical health<br>- Accessibility of healthcare
**Ogunsanya (2018)**	US	Cutaneous lupus erythematosus	Patient/patient organisation/representatives	Improved physical health	- Improved physical health<br>- Improved mental health<br>- Availability of treatments<br>- information needs<br>- Social support
**Swezey (2018)**	Unspecified	Osteogenesis imperfecta	Patients/patient organisation/representatives	Key health issues, gaps in knowledge, research priorities	- Improved physical health<br>- Improved mental health<br>- Availability of treatments<br>- Integrated, multi-disciplinary care<br>- Social support
**De Freitas (2017)**	Inter-continental	Congenital Disorders of Glycosylation	Multistakeholder	Public and patient involvement in needs assessment	- Improved physical health<br>- Financial stability<br>- Improved mental health<br>- Integrated, multi-disciplinary care<br>- Accessibility of healthcare<br>- Timely and accurate diagnosis<br>- Social support
**Pelentsov (2016)**	Australia	Orphan but non disease specific	Patients & caregivers	Supportive care needs	- Improved physical health<br>- Financial stability<br>- Integrated, multi-disciplinary care<br>- Information needs<br>- Timely and accurate diagnosis
Interviews					
**Loesken (2023)**	Europe	Primary sclerosing cholangitis	Patients/patient organisations/representatives	Patient perception	- Improved physical health<br>- Improved mental health<br>- Integrated, multi-disciplinary care<br>- Information needs

					- Timely and accurate diagnosis<br>- Social support
**Respondek (2023)**	Global	Progressive supranuclear palsy	Multistakeholder	Patient experiences	- Improved physical health<br>- Improved mental health<br>- Integrated, multi-disciplinary care<br>- Information needs<br>- Timely and accurate diagnosis
**Kleitsch (2023)**	US	Dermatomyositis	Patients/patient organisations/representatives	Improved physical healthand emotional impact	- Improved physical health<br>- Improved mental health<br>- Availability of treatments<br>- Social support
**Raspa (2023)**	US	Severe combines immunodeficiency (SCID)	Parents & caregivers	Uncertainties experienced by patients	- Improved physical health<br>- Information needs<br>- Accessibility of healthcare<br>- Timely and accurate diagnosis
**Witt (2023)**	Unspecified	Orphan but non disease specific	Patients & caregivers	Experiences/needs with medical and psychosocial care	- Improved physical health<br>- Improved mental health<br>- Improved autonomy<br>- Integrated, multi-disciplinary care<br>- Timely and accurate diagnosis<br>- Information needs<br>- Social support<br>- Support in work and education
**Potter (2023)**	Inter-continental	Men diagnosed with breast cancer (MBC)	Patients/patient organisations/representatives	Patient experiences	- Improved physical health<br>- Improved mental health<br>- Improved autonomy<br>- Availability of treatments<br>- Integrated, multi-disciplinary care<br>- Information needs<br>- Social support
**Bauskis (2022)**	Australia	Orphan but non disease specific	Parents & caregivers	Diagnostic odyssey	- Improved physical health<br>- Availability of treatments<br>- Integrated, multi-disciplinary care<br>- Social support
**Ciesluk (2022)**	US	Nuclear protein in testis (NUT) carcinoma	Patients & caregivers	Patient experiences	- Improved physical health
**Olischläger (2022)**	Unspecified	Orphan but non disease specific	Patients & HCP	Patient experiences & needs	- Integrated, multi-disciplinary care<br>- Information needs<br>- Accessibility of healthcare
**Randall (2022)**	US	Classic galactosemia	Patients & caregivers	Disease burden & daily challenges	- Improved physical health<br>- Improved mental health<br>- Improved autonomy<br>- Social support
**Garcia-Diaz (2022)**	Europe	Lysosomal Storage Diseases	Patients & HCP	Disease impact	- Improved physical health<br>- Improved mental health<br>- Improved autonomy<br>- Patient experience<br>- Information needs

					- Accessibility of healthcare<br>- Timely and accurate diagnosis<br>- Social support
**Smits (2022)**	unspecified	Orphan but non disease	Caregiver	Needs	- Improved physical health<br>- Improved mental health<br>- Integrated, multi-disciplinary care<br>- Information needs<br>- Accessibility of healthcare<br>- Social support
**Ashtari (2022)**	US	Ehlers-Danlos syndrome	/	Effectiveness of online peer support groups	- Improved mental health<br>- Availability of treatments<br>- Information needs<br>- Accessibility of healthcare<br>- Social support
**Hill (2022)**	Unspecified	Osteogenesis imperfecta	Multistakeholder	Patient experiences and needs	- Improved physical health<br>- Improved mental health<br>- Availability of treatments<br>- Integrated, multi-disciplinary care<br>- Accessibility of healthcare<br>- Social support<br>- Impact on work<br>- Financial stability
**Simpson (2021)**	Europe	Orphan but non disease specific	Patient & caregivers	Impact on patients and their carers	- Financial stability<br>- Integrated, multi-disciplinary care<br>- Accessibility of healthcare
**Cardão (2021)**	Europe	Congenital disorders of glycosylation	Parents & caregivers	Patient experience	- Improved physical health<br>- Financial stability
**Skrobanski (2021)**	Inter-continental	Aromatic l-amino acid decarboxylase (AADC) deficiency	Caregiver	Impact of caring for a patient	- Improved physical health<br>- Financial stability
**Maxfield (2021)**	Unspecified	Disorder of the corpus callosum	/	Patient experience	- Integrated, multi-disciplinary care<br>- Social support<br>- Support in work and education<br>- Impact on work
**Strugnell (2020)**	Australia	Rett syndrome	Patients & caregivers	Aspects of life that are satisfying or challenging	- Improved physical health<br>- Improved mental health<br>- Improved autonomy<br>- Integrated, multi-disciplinary care<br>- Social support
**Hiltrop (2020)**	Europe	Male breast cancer patients (MBCPs)	Patients/patient organisations/representatives	Return-to-work experiences	- Financial stability<br>- Improved physical health
**Brod (2020)**	US	Hypoparathyroidism	Patients & HCP	Patient experience	- Improved physical health
**Verrecchia (2020)**	Europe	Alpha-mannosidosis	/	Caregiver's and physician's perspectives	- Integrated, multi-disciplinary care<br>- Timely and accurate diagnosis
**Oladapo (2019)**	Unspecified	Congenital (Hereditary) Thrombotic Thrombocytopenic Purpura	Patients/patient organisations/representatives	Patient experiences	- Improved physical health<br>- Improved mental health<br>- Improved autonomy

**Baumbusch (2019)**	Canada	Orphan but non disease specific	Parents & caregivers	Parent experiences	- Financial stability<br>- Improved mental health<br>- Integrated, multi-disciplinary care<br>- Information needs<br>- Social support
**Wu (2019)**	Asia	Epidermolysis bullosa	Caregiver	Family caregivers' experiences	- Improved physical health
**Mooney (2019)**	Unspecified	Rare rheumatic condition	/	Impact on patients and informal carers	- Improved physical health<br>- Financial stability<br>- Improved physical health<br>- Improved mental health
**Babac (2018)**	Europe	Orphan but non disease specific	Multistakeholder	Patient, relatives and HCP needs	- Improved mental health<br>- Information needs<br>- Social support
**Ruijin (2018)**	Asia	Adrenoleukodystrophy	Caregiver/parents	Parents' experience	- Improved physical health<br>- Integrated, multi-disciplinary care<br>- Timely and accurate diagnosis
**Adams (2018)**	Europe	Lipodystrophy	Patients/patient organisations/representatives	Impact on body image	- Improved mental health<br>- Timely and accurate diagnosis<br>- Information needs<br>- Social support
**Sibeoni (2018)**	Europe	Neuroendocrine tumors	/	Patients and physicians perspectives	- Improved mental health
**Beynon (2015)**	Europe	Primary cutaneous T-cell lymphoma	Patients/patient organisations/representatives	Patient experiences, attitudes and approaches to coping	- Improved physical health<br>- Improved mental health<br>- Availability of Availability of treatments<br>- Timely and accurate diagnosis<br>- Social support<br>- Financial stability
**Montali (2011)**	Unspecified	Primary biliary cirrhosis	/	Patient experiences	- Integrated, multi-disciplinary care<br>- Social support
**Henderson (2009)**	US	Niemann-Pick disease	Patients & caregivers	Psychosocial aspects of patients	- Improved physical health<br>- Improved mental health<br>- Information needs<br>- Social support
**Griffiths (2007)**	Europe	Rarer cancers	Patients/patient organisations/patient representatives	Patient needs	- Improved physical health<br>- Improved mental health<br>- Integrated, multi-disciplinary care<br>- Accessibility of Availability of treatments/care<br>- Social support
**Lowton (2006)**	Europe	Cystic fibrosis	Patients/patient organisations/representatives	Patient experiences	- Financial stability<br>- Integrated, multi-disciplinary care<br>- Information needs
Report					
**Mease (2023)**	Unspecified	Orphan but non disease specific	/	Patient experiences	- Improved physical health<br>- Improved mental health

					- Availability of Availability of treatments<br>- Timely and accurate diagnosis<br>- Impact on work
**Gross (2017)**	Inter-continental	Disease(s)-specific	/	Unmet needs	- Timely and accurate diagnosis<br>- Accessibility of healthcare<br>- Social support<br>- Support in work and education
Mixed qualitative methods					
**Foster (2023)**	US	Orphan but non disease specific	Multistakeholder	Self-efficacy managing by parent	- Improved physical health<br>- Availability of treatments
**Hartford (2023)**	US	Generalized myasthenia gravis	Patients & HCP	Patient experience	- Improved physical health<br>- Availability of Availability of treatments
**Bingaman (2023)**	Inter-continental	GM1-gangliosidosis	Parents & caregivers	Disease burden	- Improved physical health<br>- Financial stability<br>- Improved autonomy<br>- Availability of treatments<br>- Social support
**Martin-gomez (2023)**	Europe	NKX2-1-related disorders	Patients & caregivers	Preferences and information needs of patients and caregivers	
**Golden (2023)**	US	Niemann-Pick disease type C	Patients & caregivers	Patient experience	- Improved physical health<br>- Improved mental health<br>- Improved autonomy<br>- Integrated, multi-disciplinary care<br>- Timely and accurate diagnosis<br>- Social support
**Engler (2022)**	Europe	Life-limiting illness	Multistakeholder	Needs for palliative care	- Accessibility of healthcare
**Teare (2022)**	Europe	Central diabetes insipidus	Patients & HCP	Challenges and improvement needs	- Financial stability<br>- Availability of treatments<br>- Integrated, multi-disciplinary care<br>- Timely and accurate diagnosis<br>- Social support
**Hanbury (2021)**	Europe	Aromatic l-amino acid decarboxylase	Multistakeholder	Needs	- Improved physical health
**Jimenez-Moreno (2021)**	Inter-continental	Neuromuscular diseases	Patients & caregivers	Patient preferences	- Improved physical health<br>- Improved mental health<br>- Availability of treatments<br>- Impact on work
**Güeita-Rodriguez (2020)**	Europe	Rett syndrome	Parents & caregivers	Access to health and social care resources	- Financial stability<br>- Availability of treatments<br>- Integrated, multi-disciplinary care<br>- Information needs<br>- Accessibility of healthcare<br>- Timely and accurate diagnosis<br>- Social support
**Khanna (2019)**	Inter-continental	Systemic sclerosis	/	Patient perception of disease burden	- Timely and accurate diagnosis<br>- Social support

**Both (2018)**	Europe	Tuberous sclerosis complex	Patients & caregivers	Concerns and care needs of young adults	- Financial stability<br>- Improved physical health<br>- Improved mental health<br>- Improved autonomy<br>- Information needs<br>- Accessibility of healthcare<br>- Social support
**Litzkendorf (2018)**	Europe	Orphan but non disease specific	Multistakeholder	To develop an information portal on RD	- Information needs
**Simoes (2017)**	Europe	/	Patients/patient organisations/representatitves	Patient expectations	- Integrated, multi-disciplinary care<br>- Information needs
**Pauer (2016)**	Europe	Orphan but non disease specific	Patients & caregivers	Patient information needs	- Information needs

4. Mixed-method studies

**Author (year)**	**Geographical region**	**Disease**	**Study design**			**NEED criteria**
			**Methods**	**Population**	**Objective**	
**Kocher (2023)**	Europe	Systemic sclerosis	- Survey<br>- Interviews<br>- Focus group discussion	Patients/ Patient organisations / Patient representatives	To describe the current state of chronic illness care and HRImproved physical healthfrom the patient perspective	- Improved physical health<br>- Integrated, multi-disciplinary care<br>- Information needs
**Hoenig (2023)**	Europe	Non-tuberculous mycobacteria (NTM)	- Survey<br>- Interviews	Patients/ Patient organisations / Patient representatives	Eliciting Patient Insights on the Burden of Nontuberculous Mycobacterial Lung Disease	- Improved physical health<br>- Availability of treatments<br>- Integrated, multi-disciplinary care<br>- Timely and accurate diagnosis
**Antoniadou (2023)**	Europe	Orphan but non-disease specific	- Survey<br>- Interviews	Multistakeholder	To identify the parameters that matter for patients, professionals and policymakers in the context of rare diseases	- Improved physical health<br>- Accessibility of healthcare<br>- Social support
**Hughes (2023)**	US	generalized myasthenia gravis	- Survey<br>- Interviews	Patients and Caregivers	To characterize how SDOH barriers impact individuals from diverse racial, ethnic, educational, and socioeconomic	- Improved physical health<br>- Information needs<br>- Accessibility of healthcare<br>- Social support<br>- Financial stability

					backgrounds living with gMG	
**Müller (2023)**	Unspecified	tuberous sclerosis complex	- Survey<br>- Interviews	Patients	To understand the impact of tuberous sclerosis complex through the development and validation of the TSC-PROM	
**Marinello (2022)**	Europe	Rare and complex rheumatic tissue diseases (rCTDs)	- Survey<br>- Focus group	Patients/ Patient organisations / Patient representatives	Exploring patient's experience and unmet needs on pregnancy and family planning in rare and complex connective tissue diseases	- Improved physical health<br>- Integrated, multi-disciplinary care<br>- Information needs
**McMullan (2022)**	Europe	Orphan but non-disease specific	- Survey<br>- Focus group		To gain an understanding of carer reported experiences derived specifically from persons caring for someone with a rare disease	- Improved physical health<br>- Financial stability<br>- Information needs<br>- Social support
**Spencer-Tansley (2022)**	Europe	Orphan but non-disease specific	- Survey<br>- Interviews	Patients and caregivers	To explore the impact on mental health of living with a rare condition, and experiences of health service support	- Improved physical health<br>- Integrated, multi-disciplinary care<br>- Accessibility of healthcare<br>- Social support
**Marcinkowska (2022)**	Europe	Tuberous Sclerosis Complex	- Survey<br>- Interviews	Patient and caregiver	Identifying the needs and difficulties of patients with Tuberous Sclerosis Complex	- Improved physical health<br>- Support in work and education
**Yang (2022)**	US	Neurofibromatosis Type1 (NF1) and Plexiform Neurofibroma (PN)	- Survey<br>- Interviews	Patients and caregivers	To gain understanding in the burden Among Caregivers of Pediatric Patients with Neurofibromatosis Type 1 (NF1) and Plexiform Neurofibroma (PN)	- Improved physical health<br>- Financial stability<br>- Improved physical health
**Godfrey (2021)**	US	Cystic fibrosis	- Survey<br>- Focus group	Patients, HCP and caregivers	To determine the educational needs and preferences	- Social support
**Long (2021)**	Australia	Mitochondrial Respiratory Chain Disorders (MRCDs)	- Survey<br>- Focus group	Patients and caregivers	Identifying the perspectives of people and parents of children concerning disease management after Timely and accurate diagnosis	- Integrated, multi-disciplinary care<br>- Information needs<br>- Accessibility of healthcare<br>- Social support

**Depping (2021)**	Europe	neurofibromatosis type 1; primary sclerosing cholangitis; pulmonary arterial hypertension; and Marfan syndrome	- Survey<br>- Focus group	Patients/patient organisations/patient representatives	The development of a self-care intervention for patients with rare diseases	- Improved physical health<br>- Accessibility of healthcare<br>- Social support
**Applebaum (2020)**	US	Erdheim–Chester disease	- Survey<br>- Interviews	Caregivers and parents	Understanding the burden of caregivers	- Improved physical health<br>- Financial stability
**McMullan (2020)**	Europe	Orphan but non disease specific	- Survey<br>- Interviews	Patients/patient organisations/patient representatives	Identifying which improvements are required to further support people living with a rare disease	- Improved physical health<br>- Information needs<br>- Social support
**Porter (2020)**	US	Mucopolysaccharidosis type III (MPS III, Sanfilippo syndrome)	- Survey<br>- Focus group	Patient and caregivers	Identifying the unmet Availability of treatments needs	- Improved physical health<br>- Availability of treatments<br>- Information needs<br>- Accessibility of healthcare<br>- Social support
**Inhestern (2020)**	Europe	Orphan but non disease specific	-		Developing best practice recommendations on management concepts in healthcare for rare diseases	
**Ragusa (2020)**	Europe	Prader-willi	- Survey<br>- Interviews	Patients and caregivers	Gaining insights in the needs of patients and caregivers	- Improved physical health<br>- Timely and accurate diagnosis
**Kocher (2019)**	Europe	Systemic sclerosis	- Survey<br>- Interviews	Patient, HCP and caregivers	Gaining insights into the chronic illness care and disease management	- Integrated, multi-disciplinary care
**Noël (2019)**	Europe	Fabry Disease	- Survey<br>- Interviews	Patients/patient organisations/patient representatives	Identification of Availability of treatments needs and expectations	- Availability of treatments
**Svernling (2019)**	Europe	Locked-in syndrome	- Database analysis<br>- Interviews	Patients/patient organisations/patient representatives	Assessment of the Improved physical health	- Improved physical health<br>- Integrated, multi-disciplinary care<br>- Social support
**Erbis (2018)**	Europe	Autoinflammatory diseases	- Survey<br>- Focus group	Patients and caregivers	Evaluating the patient perspective of the unmet needs in AID	- Improved physical health<br>- Financial stability<br>- Improved physical health<br>- Information needs<br>- Accessibility of healthcare<br>- Timely and accurate diagnosis<br>- Social support
**Hiremath (2018)**	US	Eosinophilic gastrointestinal disorders (EGID)	- Survey<br>- Focus group	Patients and caregivers	Identification of the unmet medical needs and barriers in patients with EGID	- Improved physical health<br>- Integrated, multi-disciplinary care<br>- Accessibility of healthcare

						- Social support
**Tejada-Ortigosaa (2017)**	Europe	Rare metabolic diseases	- Survey<br>- Interviews	Patients and caregivers	Identification of the health and socio-educational needs	- Improved physical health<br>- Financial stability<br>- Improved physical health<br>- Integrated, multi-disciplinary care<br>- Accessibility of healthcare<br>- Timely and accurate diagnosis<br>- Social support
**Kasparian (2015)**	Australia	Von Hippel–Lindau disease	- Survey<br>- Interviews	Patients and caregivers	Eliciting the experiences and unmet medical needs of patients their families and caregivers	- Improved physical health<br>- Integrated, multi-disciplinary care<br>- Information needs<br>- Timely and accurate diagnosis<br>- Social support
**Van Groenendael (2015)**	Europe	Alstrom syndrome	- Survey<br>- Interviews	Patients and caregivers	Analyzing he effectiveness of the MDC	- Integrated, multi-disciplinary care
**Behan (2016)**	Europe	Primary ciliary dyskinesia	- Survey<br>- Interviews	Patients/patient organisations/patient representatives	To investigate the patients' perspectives on Timely and accurate diagnosis between	- Timely and accurate diagnosis<br>- Information needs<br>- Social support
**Dwyer (2014)**	Europe	Congenital hypogonadotropic hypogonadism	- Survey<br>- Focus group	Patients/patient organisations/patient representatives	Identifying the unmet health needs of patients	- Improved physical health<br>- Integrated, multi-disciplinary care<br>- Information needs<br>- Social support
